# Effect of exercise on functional capacity and body weight for people with hypertension, type 2 diabetes, or cardiovascular disease: a systematic review with meta-analysis and trial sequential analysis

**DOI:** 10.1186/s13102-024-00829-1

**Published:** 2024-02-07

**Authors:** Anupa Rijal, Tara Ballav Adhikari, Sarmila Dhakal, Mathias Maagaard, Reza Piri, Emil Eik Nielsen, Dinesh Neupane, Janus Christian Jakobsen, Michael Hecht Olsen

**Affiliations:** 1 Department of Regional Health Research, University of Southern Denmark, Odense, Denmark; 2 Department of Internal Medicine, Holbaek Hospital, Holbaek, Denmark; 3 Department of Public Health, Research Unit for Environment, Occupation & Health, Aarhus University, Aarhus, Denmark; 4 Center for Research on Environment, Health and Population Activities (CREPHA), Kusunti, Lalitpur, Nepal; 5 Centre for Anaesthesiological Research, Department of Anaesthesiology, Zealand University Hospital, Koge, Denmark; 6 Department of Clinical Research, University of Southern Denmark, Odense, Denmark; 7 Department of Nuclear Medicine, Odense University Hospital, Odense, Denmark; 8 Department of Cardiology, Odense University Hospital, Odense, Denmark; 9 Department of International Health, Johns Hopkins Bloomberg School of Public Health, Johns Hopkins University Baltimore, Maryland, USA; 10 Copenhagen Trial Unit, Centre for Clinical Intervention Research, Copenhagen University Hospital – Rigshospitalet, Copenhagen, Denmark

**Keywords:** Exercise, Hypertension, Type 2 diabetes, Cardiovascular disease, Functional capacity, Vo_2_max, 6mwt, Body weight

## Abstract

**Background:**

Hypertension, type 2 diabetes, and cardiovascular disease affect the activities of daily living at varying degree. While the effects of aerobic exercise on functional capacity are well-documented, the extent of change for different types of exercise in these chronic conditions remains unexplored. Additionally, there is conflicting evidence regarding the role of exercise in reducing body weight.

**Methods:**

We conducted systematic review with meta-analysis and trial sequential analysis and searched various databases from inception to July 2020. We included randomised clinical trials adding any form of trialist defined exercise to usual care versus usual care in people with either hypertension, type 2 diabetes, and/or cardiovascular disease irrespective of setting, publication status, year, and language. The outcomes assessed were i) functional capacity assessed through different scales separately i.e., Maximal Oxygen Uptake (VO_2_max), 6-min walk test (6MWT), 10-m walk test (10MWT), and ii) body weight.

**Results:**

We included 950 studies out of which 444 trials randomising 20,098 participants reported on various functional outcomes (355 trials) and body weight (169 trials). The median follow-up was 3 months (Interquartile ranges (IQR): 2.25 to 6). Exercise added to the usual care, improved VO_2_max (Mean Difference (MD):2.72 ml/kg/min; 95% Confidence Interval (CI) 2.38 to 3.06; *p* < 0.01; I^2^ = 96%), 6MWT (MD: 42.5 m; 95%CI 34.95 to 50.06; *p* < 0.01; I^2^ = 96%), and 10MWT (MD: 0.06 m/s; 95%CI 0.03 to 0.10; *p* < 0.01; I^2^ = 93%). Dynamic aerobic and resistance exercise showed a consistent improvement across various functional outcomes, whereas body-mind therapies (MD: 3.23 ml/kg/min; 95%CI 1.97 to 4.49, *p* < 0.01) seemed especially beneficial for VO_2_max and inspiratory muscle training (MD: 59.32 m; 95%CI 33.84 to 84.80; *p* < 0.01) for 6MWT. Exercise yielded significant reduction in body weight for people with hypertension (MD: -1.45 kg; 95%CI -2.47 to -0.43; *p* < 0.01), and type 2 diabetes (MD: -1.53 kg; 95%CI -2.19 to -0.87; *p* < 0.01) but not for cardiovascular disease with most pronounced for combined exercise (MD: -1.73 kg; 95%CI -3.08 to -0.39; *p* < 0.05). The very low certainty of evidence warrants cautious interpretations of the results.

**Conclusion:**

Exercise seemed to improve functional capacity for people with hypertension, type 2 diabetes, and/or cardiovascular disease but the effectiveness seems to vary with different forms of exercise. The potentially superior improvement in VO_2_max and 6MWT by body-mind therapies and inspiratory muscle training calls for further exploration. Additionally, prescribing exercise for the sole purpose of losing weight may be a potential strategy for people with hypertension and type 2 diabetes. The extent of improvement in functional capacity and body weight reduction differed with different exercise regimens hence personalised exercise prescriptions tailored to individual needs may be of importance.

**PROSPERO registration:**

PROSPERO registration number: CRD42019142313.

**Supplementary Information:**

The online version contains supplementary material available at 10.1186/s13102-024-00829-1.

## Background

Functional capacity in broad terms refers to an individual’s ability to perform physical tasks such as walking, climbing, and other daily activities without experiencing undue fatigue or physical stress [1]. Hypertension, type 2 diabetes, and cardiovascular disease are the leading non-communicable disease globally that affect the activities of daily living at varying degree [2]. For instance, patients with cardiovascular disease, especially for conditions like coronary artery disease, heart failure, or cardiomyopathy is characterized by reduced cardiac output leading to shortness of breath, fatigue and muscle weakness depending on severity of the condition [1, 3]. Likewise, the most common consequence of stroke leads to hemiparesis or spasticity which limits individual’s mobility and may have severe cognitive impairment affecting the autonomy in activities of daily living [4]. Hypertension may lead to hypertension-related structural and functional changes in target-organs like heart, kidneys and brain [5]. impacting an individual’s stamina and mobility. In case of type 2 diabetes, complications like neuropathy can hamper neuromuscular function leading to difficulties in walking and other fine motor skills [6].

Evidence have shown that impaired functional capacity is an effective predictor of cardiovascular disease risk and even mortality [7, 8]. Functional capacity is objectively measured through maximal/peak oxygen uptake (VO_2_max). VO_2_max is the uptake or consumption of maximal oxygen during exercise and is considered gold standard to evaluate individuals’ cardiovascular fitness level. Walk tests such as six-minute walk test (6MWT), or ten-meter walk test (10MWT) [8–10]. are comparatively simple, inexpensive, safe, and reproducible tools for assessing aerobic fitness [8, 11]. Apart from this, muscular strength and balance are other important elements of functional capacity [12].

Regular exercise is considered an important element in enhancing functional capacity for individuals with hypertension, type 2 diabetes, and cardiovascular disease. These three conditions, while distinct, share a common underlying pathophysiology in how exercise influences overall wellbeing [13]. Previous reviews have reported the beneficial effect of exercise in increasing functional capacity for hypertension [14]. type 2 diabetes [15]. or cardiovascular disease [1, 8, 16]. However, such findings are often limited to common forms of exercise like aerobic or resistance exercise, and the effect of diverged forms of exercise remains inconsistent. Thus, an umbrella summary of the effect of different types of exercise on functional capacity in these cardiometabolic conditions seems necessary. We have not identified any systematic reviews that have included all forms of exercise and comprehensively assessing the effect on different functional capacity outcomes.

Additionally, individuals with hypertension, type 2 diabetes, and cardiovascular disease are recommended to engage in exercise not only for maintaining a healthy body weight but also for weight reduction [17–19]. particularly for overweight and obese individual [20, 21]. There is evidence both supporting and contradicting [20, 22–24]. the effectiveness of exercise as a standalone strategy for reducing body weight. Thus, an overview of different forms of exercise on body weight for these leading cardiometabolic conditions could significantly contribute to the existing pool of evidence.

Therefore, we aimed to perform a systematic review with meta-analysis and trial sequential analysis to assess the effect of different forms of exercise on functional capacity and body weight for people with hypertension, type 2 diabetes or cardiovascular disease when added to their usual care. Additionally, we also wanted to investigate if the exercise induced changes in functional capacity can explain the reduction in all-cause mortality reported in our previously published paper [25].

## Methods

We described our methodology in detail in our protocol registered and published prior to the systematic literature search [26]. We reported this systematic review according to the Preferred Reporting Items for Systematic Reviews and Meta-Analysis (PRISMA) guidelines [27]. We included all randomised clinical trials assessing the effect of adding any of form exercise (as defined by trialists) to usual care (as defined by trialists-any routine care received by the patients) versus usual care (same usual care as in the intervention group). We included any form of co-interventions, if the co-intervention is intended to be delivered similarly to the intervention and control groups. We included people with either hypertension, type 2 diabetes, and/or cardiovascular disease irrespective setting, trial duration, publication status, publication year, and language.

We searched the database Cochrane Central Register of Controlled Trials (CENTRAL), Medical Literature Analysis and Retrieval System Online (MEDLINE), Excerpta Medica database (EMBASE), Science Citation Index Expanded on Web of Science, BIOSIS, google scholar and clinicaltrials.gov from inception till July 2020. Additionally, we also manually searched reference lists of previously published reviews for relevant publications.

The detailed search strategy can be found in (text S1).

### Data extraction strategy

We extracted data using standardised data extraction sheet. Five authors (AR, TBA, SD, MM, RP) extracted information on trials’ characteristics (gender, country, number of participants in intervention and control, length of intervention, follow-up period, baseline information- age, body mass index, medication) and characteristics of exercise intervention such as type of exercise, volume of exercise (hours/week), intensity of exercise.

Information on exercise intensity if not explicitly mentioned in the trials were categorised to low, moderate or vigorous based on Oxygen uptake Reserve (VO_2_R%), Heart Rate Reserve (HRR%), Age- predicted maximal heart rate (HRmax%), Ratings of perceived exertion (RPE) parameters as per guidelines presented in General Principles of Exercise Prescription [28]. which has been adapted from American College of Sports Medicine Guidelines for Exercise Testing, 8th edition [29]. and Prescription and Physical Activity Guidelines Advisory Committee Report, USA [30]. We resolved disagreements through discussion or consulting with a third author (JCJ or EEN). If data were missing or unclear, we attempted to contact authors through email.

### Risk of bias

We assessed risk of bias using the Cochrane Risk of Bias- version 1 (RoB1) [31]. and assessed the following bias domains: random sequence generation, allocation concealment, blinding of people and personnel, blinding of outcome assessment, incomplete outcome data, selective outcome reporting, for profit bias, and other risks of bias. We classified trials as being at overall “high risk of bias”, if any of the bias domains are classified as “unclear” or “high risk of bias”.

### Outcomes and subgroup analyses

Our outcomes were i) functional capacity assessed through VO_2_max (ml/kg/min), 6MWT (m), 10MWT/Gait velocity (m/s), Berg balance scale, Timed Up and Go Test (TUGT,seconds), Exercise Capacity (measured in Watt and MET) and ii) body weight (kg) reported at maximum follow-up.

Primary outcomes (all-cause mortality, serious adverse events, quality of life) and other secondary outcomes from this review has been published elsewhere [25].

We prespecified several subgroup analyses (see Results): 1) different types of exercise, 2) different disease groups (hypertension, type 2 diabetes, or cardiovascular disease as defined by trialists or cardiovascular disease as defined by WHO that includes cerebrovascular disease, rheumatic heart disease, deep vein thrombosis, pulmonary thrombosis, coronary artery disease such as myocardial infarction, and heart failure), 3) High Income countries (HICs) vs. Low-middle income countries (LMICs), 4) trials at high risk of bias compared to trials at low risk of bias.

In addition, we have added further post-hoc subgroup analyses: 1) trials including biological male compared to biological female compared to trials including both biological sexes, 2) short term follow up (≤ median follow-up) compared to long term follow up (> median follow-up). We also additionally conducted subgroup analysis for VO_2_max, 6 MWT, 10 MWT, and body weight based on 3) age in years (≤ median age compared to > median age) 4) baseline Body Mass Index (BMI) (normal < 25 kg/m^2^; overweight ≥ 25 to ≤ 29.9 kg/m^2^; obese ≥ 30 kg/m^2^) 5) size of trials (trials with ≤ 100 people compared to trials with > 100 people) 6) type of control (usual care compared to no intervention compared to co-intervention).

### Data Analysis

We used STATA 17 (StataCorp) for all statistical analyses [32]. We considered a p value of 0.05 as the threshold for statistical significance for functional capacity and body weight due to the hypothesis generating nature of analyzing predefined exploratory outcomes [26]. We conducted both fixed-effect and random-effects meta-analysis and primarily reported the most conservative result and considered the less conservative result as sensitivity analysis [26, 33]. We analyzed different functional capacity measures separately to avoid the methodological problems with using standardized mean difference [34]. The predetermined minimal important difference for functional capacity and body weight was calculated as the mean difference of the observed Standard Deviation (SD) divided by two in the control group [33, 35]. We investigated possible heterogeneity by visual inspection of forest plots, by calculating inconsistency (I^2^), and by performing subgroup analysis (test of interaction).

In order to further assess the potential sources of heterogeneity we performed random effect stepwise meta-regression [36]. with forward selection. We regressed intervention/exercise specific co-variates (length of exercise program, volume of exercise) and patient specific co-variates (type of participants, age of participants and body mass index) separately (univariate regression) against the functional capacity measures and body weight to select variables for inclusion in meta-regression models. We used a significance level of 10% to select variables for the multivariable models; however only those with a *p* < 0.05 were considered significant in the final model. If one of the categories of categorical variable was statistically significant, all the categories of the variable were kept in the model.

Additionally, a random effect model regression analysis was performed to evaluate the association between change in VO_2_max and all-cause mortality, reported previously. The logarithm of relative risk of each trial was regressed against the difference in mean VO_2_max for participants assigned to exercise intervention and control group at the end of maximum follow-up and the statistically significant was assessed using the Wald test [36].

We assessed small study bias through funnel plots and regression asymmetry test (Egger’s test) [37]. We performed trial sequential analysis to control for the risks of type I errors and type II errors [38]. We used Grading of Recommendations Assessment, Development and Evaluation (GRADE) to assess the certainty of evidence [39, 40].

## Result

### Characteristics of study

**Fig. 1:**
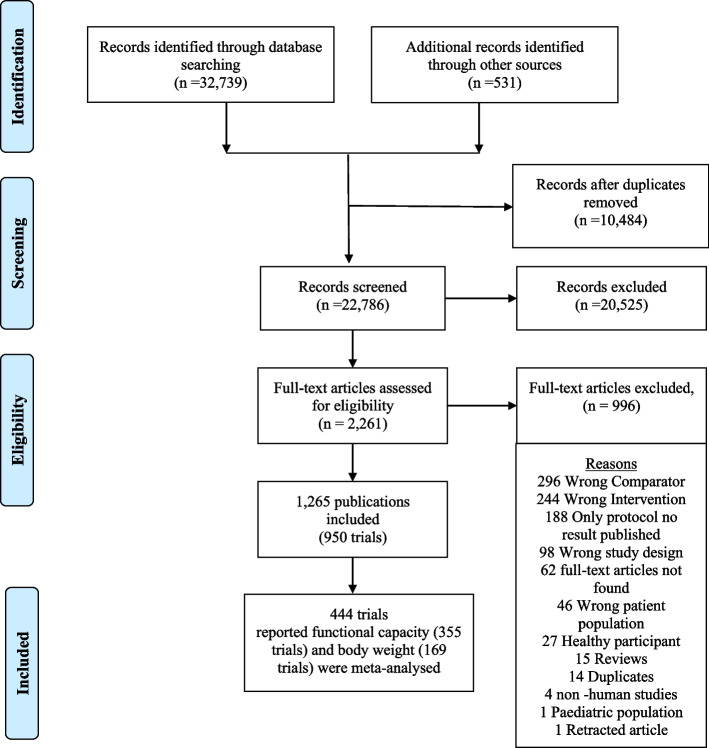
Preferred Reporting Items for Systematic Reviews and Meta-Analyses flow diagram

We identified 32,739 potentially relevant references through our literature search conducted on July 6, 2020. We included 950 studies out of which 444 unique studies randomising 20,098 people reporting on functional outcomes (355 trials) and body weight (169 trials) were meta-analysed (Fig. 1) in this review.

Most trials included both male and female participants. The number of people in each trial ranged from 10 [41]. to 380 [42]. The median intervention follow-up period was 3 months (IQR: 2.5 to 6 months). Most trials (60%) included people with cardiovascular diseases. The most frequently reported exercise intervention was dynamic aerobic exercise in (60%) trials. The majority of included trials (76%) were conducted in HICs (Table 1). The median duration of the exercise interventions was 135 min/week (IQR: 90 to 180 min/week) and intensity varied from low to vigorous**.** The mean age of participants in intervention group was 58.6 (± 8.3) years and they were overweight with a BMI of 28.7 (± 5.4) kg/m^2^. The baseline characteristics of included studies is presented in Table 2. Further information on included trials is described in Table S1.

**Table 1:** Summary of characteristics of included studies

Study Characteristics	*n*(%)
**Sex (*n* = 444)**	
Trials with male people only	38(8.6)
Trials with female people only	39(8.8)
Trials with both male and female people	367(82.6)
**Type of People (*n* = 444)**	
Cardiovascular Disease	267(60.1)
Type 2 Diabetes	118(26.6)
Hypertension	47(10.6)
Cardiovascular Disease/ + Type 2 Diabetes + /Hypertension	12(2.7)
**Trials from Economic Region (*n* = 444)**	
High Income Countries	340(76.6)
Low-and Middle- Income Countries	104(23.4)
**Type of Exercise Intervention (*n* = 444)**	
Dynamic Aerobic Exercise	270(60.8)
Dynamic Resistance Exercise	55(12.4)
Combined Exercise	76(17.1)
Body Mind Therapies	21(4.7)
Isometric Resistance Exercise	4(0.9)
Inspiratory Muscle Training	6(1.4)
Stroke Functional Exercise	12(2.7)
**Median Exercise Intervention Period (*n* = 444)**	3 months (IQR: 2 to 4.5 months)
**Median Follow-up Period (*n* = 444)**	3 months (IQR: 2.5 to 6 months)
**Median Volume of Exercise (*n* = 172)**	135 min/week (IQR: 90 to 180 min/week)

**Table 2:** Baseline characteristics of included studies

	**Trials providing information**	**Intervention**	**No· Analysed (Intervention)**	**Trials providing information**	**Usual Care**	**No Analysed (Usual Care)**
**Age-years (SD)**	377	58.6(8.3)	8826	351	58.7(8.5)	6247
**Male sex- n(%)**	319	5401 (63.7)	8485	278	4262(62.2)	6852
**Female sex-n(%)**	319	3084(36.3)	8485	278	2590(37.8)	6852
**BMI**	162	28.7(5.4)	3335	144	29.2(6.5)	2527
**Baseline Medications n(%)**						
**Anti-hypertensive drugs(not classified)**	35	709(73.5)	965	24	545(69.7)	782
**Beta-Blockers**	144	2457(64.2)	3830	123	2006(66.1)	3034
**Diuretics**	111	1902(61.6)	3086	93	1522(62.0)	2453
**ACEI**	140	2769 (77.2)	3589	120	2201(81.5)	2698
**Calcium Channel Blockers**	51	405(26.1)	1551	40	337(26.7)	1262
**Nitrates**	39	468(44.4)	1055	35	412(47.5)	868
**ARB**	19	174(31.2)	557	17	166(34.9)	476
**Digitalis**	20	174(41.6)	308	19	157(56.5)	278
**Diagoxin**	34	359(44.5)	806	32	349(46.4)	752
**Aspirin (Anti-coagulant)**	40	947(87.8)	1079	33	741(94.9)	785
**Acetylsalycylic acid**	7	136(98.5)	138	6	143(93.4)	153
**Lipid Lowering Drugs (Statin, fibrate, omega)**	79	1527(69.3)	2203	60	1232(75.4)	1634
**Glycaemic Control**						
**Metformin**	28	577(61.6)	937	21	411(55.6)	739
**Insulin**	18	68(16.7)	408	14	74(23.5)	315
**Oral Hypoglycaemic Agents(OHA)**	44	771(78.1)	987	36	563(77.5)	726
**Insulin + OHA**	20	239(43.7)	547	14	212(49.8)	426

*SD* Standard Deviation, *BMI* Body Mass Index, *ACEI* Angiotensin-Converting Enzyme Inhibitor, *ARB* Angiotensin Receptor Blockers

### Effect of exercise on VO_2_max

**Fig. 2:**
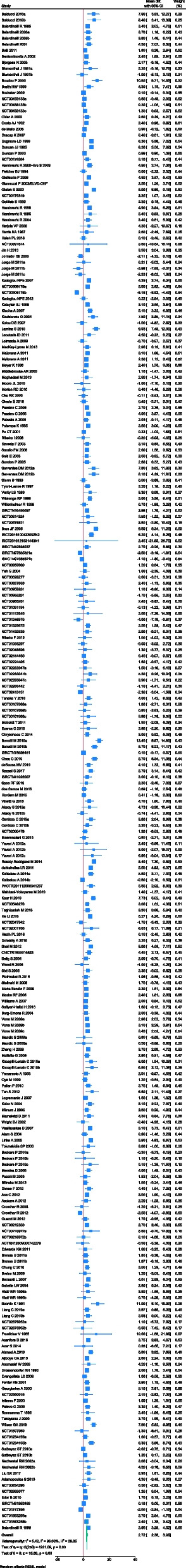
Forest plot on trials reporting VO_2_max

**Fig. 3:**
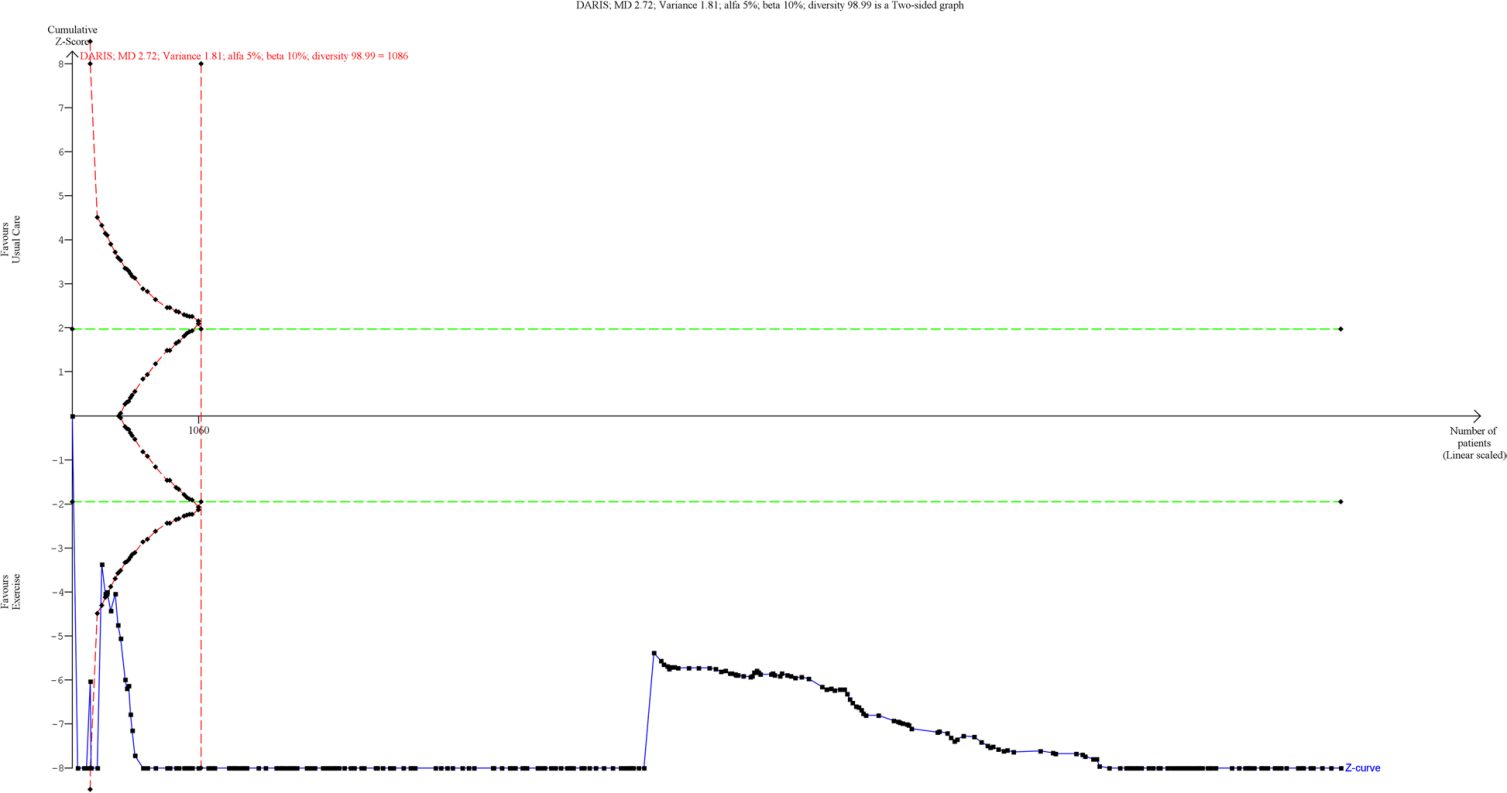
Trial sequential analysis of participants on VO_2_max

A total of 251 studies randomising 11,075 people reported on VO_2_max with median follow-up of 3 months (IQR: 2.5 to 7.5). Meta-analysis showed that exercise significantly improve VO_2_max (MD: 2.72 ml/kg/min; 95%CI 2.38 to 3.06; *p* < 0.01) and the effect is higher than predetermined level of minimal importance (2 ml/kg/min). Visual inspection of forest plot (Fig. 2) and I^2^ statistics indicated substantial signs of heterogeneity which could not be resolved (I^2^ = 96.6%). Trial sequential analysis showed that there was enough information to confirm that exercise improved VO_2_max (Fig. 3). Funnel plot and egger’s test (*p* = 0.49) indicated no small study bias (Figure S1). We assessed this outcome result as high risk of bias (Figures S2 and S3) and the certainty of evidence as very low (Table 3).

**Table 3:** Summary of Findings

Adding exercise to usual care for functional capacity and body weight for people with hypertension, type 2 diabetes and cardiovascular diseases				
**Patient or population: **People with hypertension, type 2 diabetes, or cardiovascular disease **Intervention: **exercise **Comparison: **usual care				
**Outcomes**	**No of people (studies) Follow-up**	**Certainty of the evidence (GRADE)**	Anticipated absolute effects*	
			**Risk with usual care**	**Risk difference with exercise**
Maximal Oxygen Uptake (VO2max) assessed with ml/kg/min follow-up: median 3 months	11075 (251 RCTs)	**⨁**◯◯◯ Very low^ab^	The mean VO2 max was **20.78** ml/kg/min	MD **2.72 ml/kg/min higher** (2.38 higher to 3.06 higher)
6 min walk test (6MWT) assessed with m follow-up: median 3 months	6301 (117 RCTs)	**⨁**◯◯◯ Very low^ab^	The mean 6MWT was **371.52** m	MD **42.5m higher** (34.95 higher to 50.06 higher)
10 meter walk test (10MWT) assessed with m/s follow-up: median 3 months	2646 (39 RCTs)	**⨁**◯◯◯ Very low^abc^	The mean 10MWT was **0.29** m/s	MD **0.064 m/s higher** (0.026 higher to 0.103 higher)
Body weight assessed with kg follow-up: median 3 months	7535 (169 RCTs)	**⨁**◯◯◯ Very low^ab^	The mean body weight was **77.62** kg	MD **1.42 kg** fewer (1.91 fewer to 0.92 more)

*The risk in the intervention group (and its 95% confidence interval) is based on the assumed risk in the comparison group and the relative effect of the intervention (and its 95% CI). ***CI*** confidence interval, ***MD*** mean difference, ***m*** meter, ***ml/kg/min***, milliliter/kilogram/minute, ***m/s*** meter/second, ***kg***: kilogram

GRADE Working Group grades of evidence High certainty: we are very confident that the true effect lies close to that of the estimate of the effect. Moderate certainty: we are moderately confident in the effect estimate: the true effect is likely to be close to the estimate of the effect, but there is a possibility that it is substantially different. Low certainty: our confidence in the effect estimate is limited: the true effect may be substantially different from the estimate of the effect. Very low certainty: we have very little confidence in the effect estimate: the true effect is likely to be substantially different from the estimate of effect

a. Downgraded one for risk of bias, as most of the domains were unclear in risk of bias assessment

b. Downgraded two for inconsistency, as test for heterogeneity (I^2^) was substantial (>90%)

c. Downgraded one for imprecision, as trial sequential analysis reported there was not enough information to confirm the effect of exercise

**Fig. 4:**
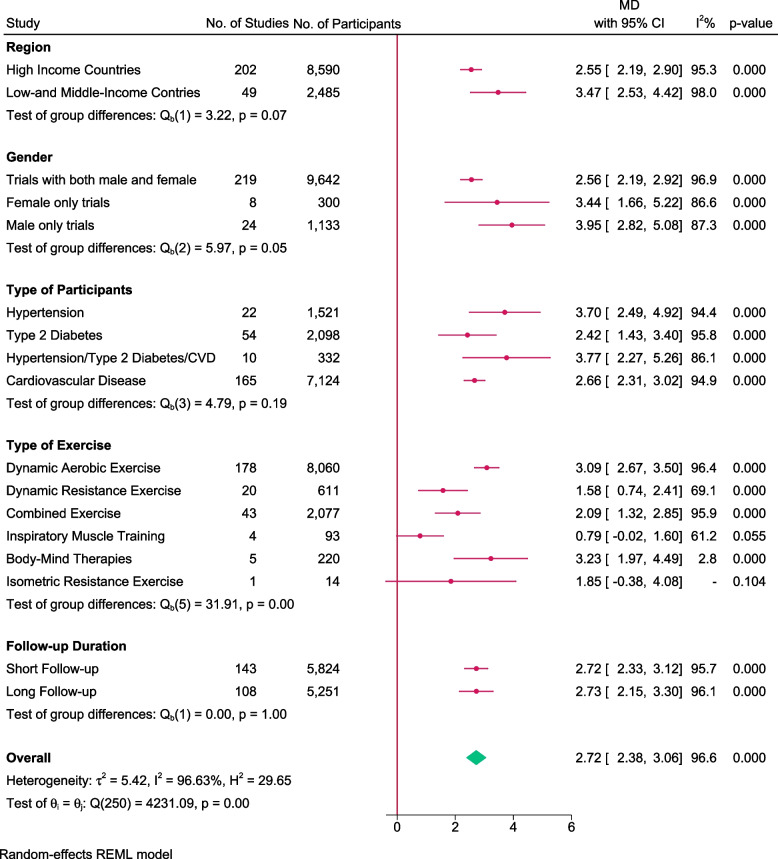
Forest plot of subgroup analysis on VO_2_max

Test of interaction showed evidence of difference when comparing trials randomising different types of exercise (Q = 31.91; *p* < 0.05) (Fig. 4). When analysed separately, the meta-analysis showed that exercise improved VO_2_max for participants following body mind therapies(MD: 3.23 ml/kg/min; 95%CI 1.97 to 4.49, *p* < 0.01), dynamic aerobic exercise (MD: 3.09 ml/kg/min; 95%CI 2.67 to 3.50; *p* < 0.01), dynamic resistance exercise (MD: 1.58 ml/kg/min; 95%CI 0.74 to 2.41; *p* < 0.01) and combined exercise(MD: 2.09 ml/kg/min; 95%CI 1.34 to 2.84; *p* < 0.01), but not for inspiratory muscle training (MD: 0.79 ml/kg/min; 95%CI -0.02 to 1.59; *p* = 0.05) and isometric resistance exercise (MD: 1.85 ml/kg/min; 95%CI -0.38 to 4.08; *p* = 0.10).

None of the remaining planned subgroup analysis showed evidence of a difference (Fig. 4 and Table S2).

### Effect of exercise on 6MWT

**Fig. 5:**
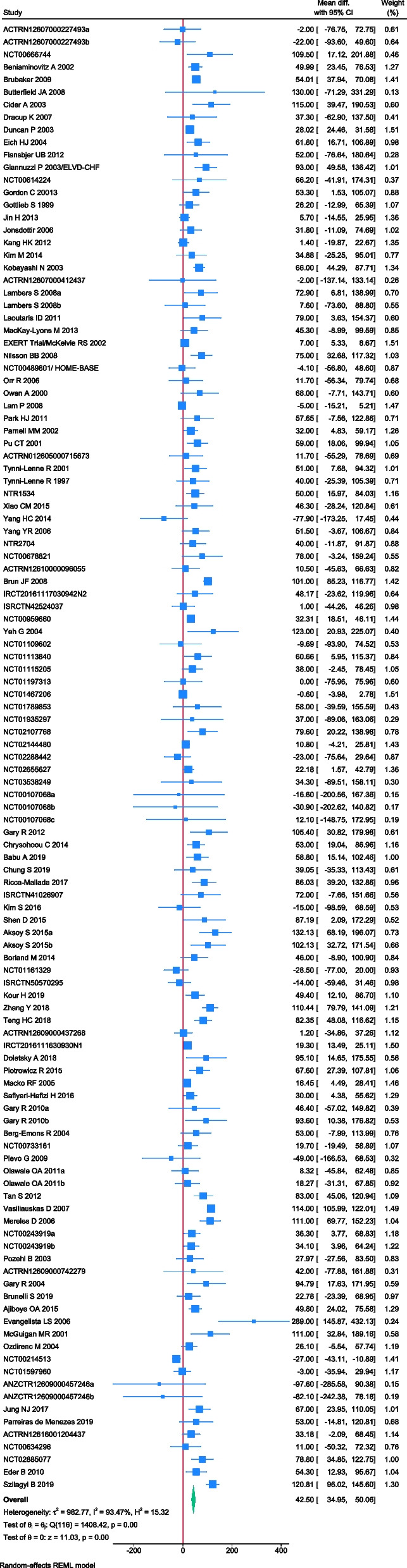
Forest plot on trials reporting 6MWT

**Fig. 6:**
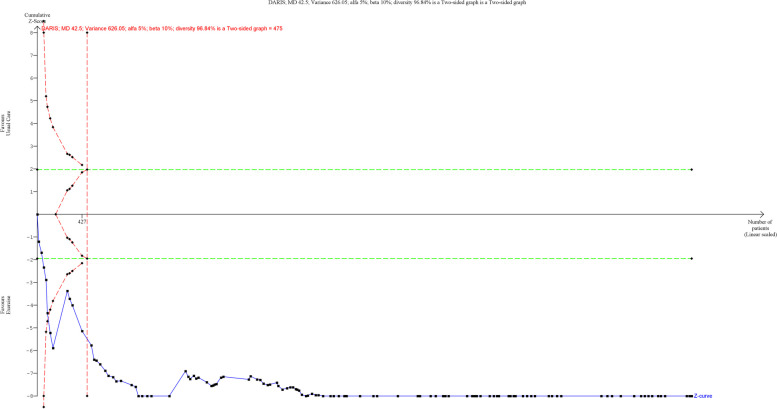
Trial sequential analysis of participants on 6MWT

A total of 117 trials randomising 6,301 people reported on 6MWT with median follow up of 3 months (IQR: 2.5 to 6 months). Meta-analysis showed that exercise significantly improve 6MWT (MD: 42.5 m; 95%CI 34.95 to 50.06; *p* < 0.01). The pre-determined minimal important difference 45 m lies within the CI of the effect estimate. Visual inspection of forest plot (Fig. 5) and I^2^ statistics indicated substantial heterogeneity which could not be resolved (I^2^ = 93.5%). Trial sequential analysis showed that there was enough information to confirm that exercise improved 6MWT (Fig. 6). Funnel plot and egger’s test (*p* = 0.36) indicated no small study bias (Figure S4). We assessed this outcome result as high risk of bias (Figures S5-S6) and the certainty of evidence as very low (Table 3).

**Fig. 7:**
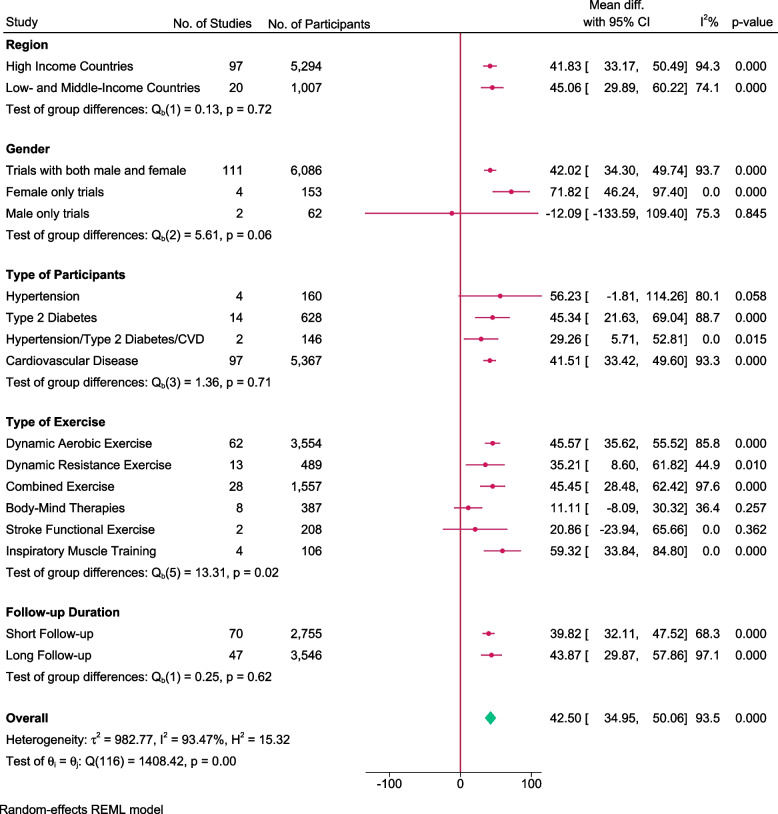
Forest plot of subgroup analysis on 6MWT

Test of interaction showed evidence of difference when comparing trials randomising different types of exercise (Q = 13.31; *p* < 0.05) (Fig. 7) When analysed separately, the meta-analysis showed that inspiratory muscle training (MD: 59.32 m; 95%CI 33.84 to 84.80; *p* < 0.01), dynamic aerobic exercise (MD: 45.57 m; 95% CI 35.62 to 55.52; *p* < 0.01), combined exercise (MD: 45.45 m; 95%CI 28.48 to 62.42; *p* < 0.01), dynamic resistance exercise (MD: 35.21 m; 95%CI 8.60 to 61.82; *p* < 0.05) improved 6MWT, but body mind therapies (MD: 11.11 m; 95%CI -8.09 to 30.32;*p* = 0.25) and stroke functional exercise (MD: 20.86 m; 95%CI -23.94 to 65.66; *p* = 0.36) did not.

Test of interaction showed evidence of difference when comparing trials randomising baseline BMI category (117 trials; Q = 6.44, *p* < 0.05). When analyzed separately, the meta-analysis showed greater improvement in 6MWT for people with obesity (MD = 57.4 m; 95%CI 29.74 to 85.06; *p* < 0.01) and overweight (MD: 50.09 m; 95% CI 32.34 to 67.86; *p* < 0.01) compared to people with normal BMI (MD: 20.96 m; 95%CI 1.52 to 40.4; *p* < 0.05).

None of the remaining planned subgroup analysis showed evidence of a difference (Fig. 7 and Table S3).

### Effect of exercise on 10MWT

**Fig. 8:**
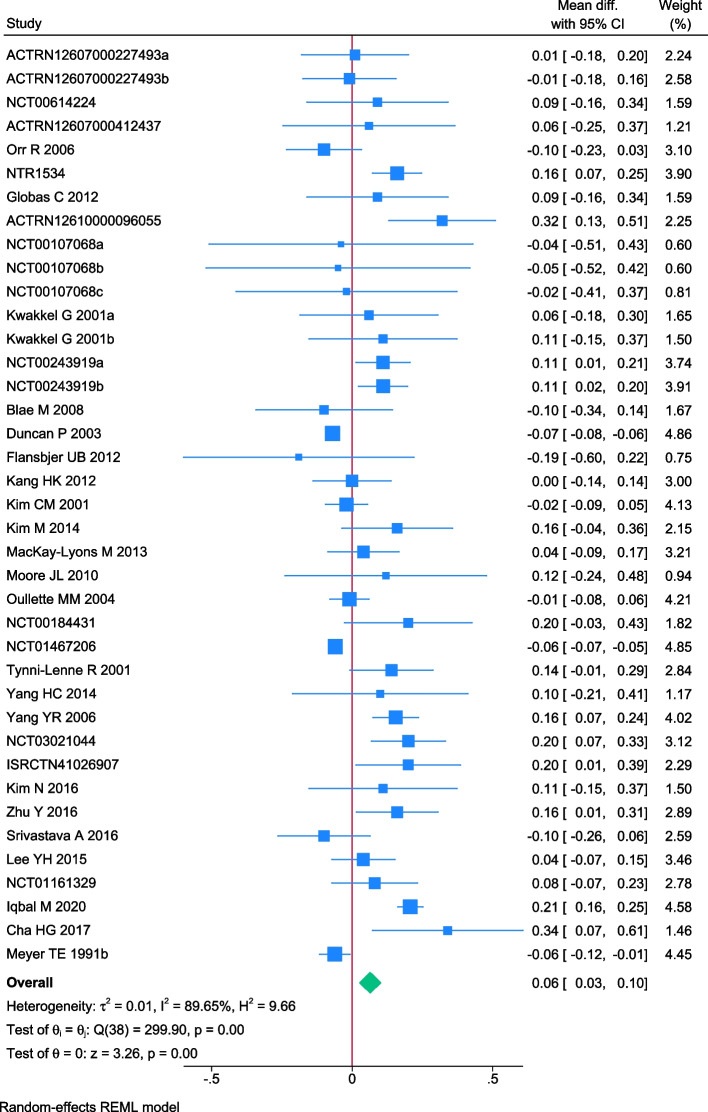
Forest plot on trials reporting 10MWT

**Fig. 9:**
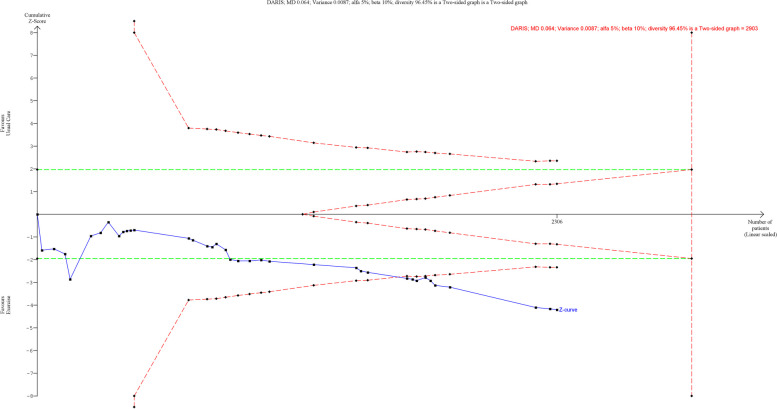
Trial sequential analysis of participants on 10MWT

A total of 39 trials randomising 2646 people reported on 10 MWT with median follow up of 3 months (IQR: 1 to 6.5 months). Meta-analysis showed that exercise significantly improved 10MWT (MD: 0.06 m/s; 95%CI 0.03 to 0.10; *p* < 0.01), but the effect was lower than the predetermined minimal clinical important difference (0.14 m/s). Visual inspection of forest plot (Fig. 8) and I^2 ^statistics indicated substantial signs of heterogeneity which could not be resolved (I^2^ = 89.6%). Trial sequential analysis showed that there was not enough information to confirm that exercise improved 10MWT (Fig. 9). Funnel plot and egger’s test (*p* = 0.05) indicated no small study bias (Figure S7). We assessed this outcome result as high risk of bias (Figures S8 and S9) and the certainty of evidence as very low (Table 3).

**Fig. 10:**
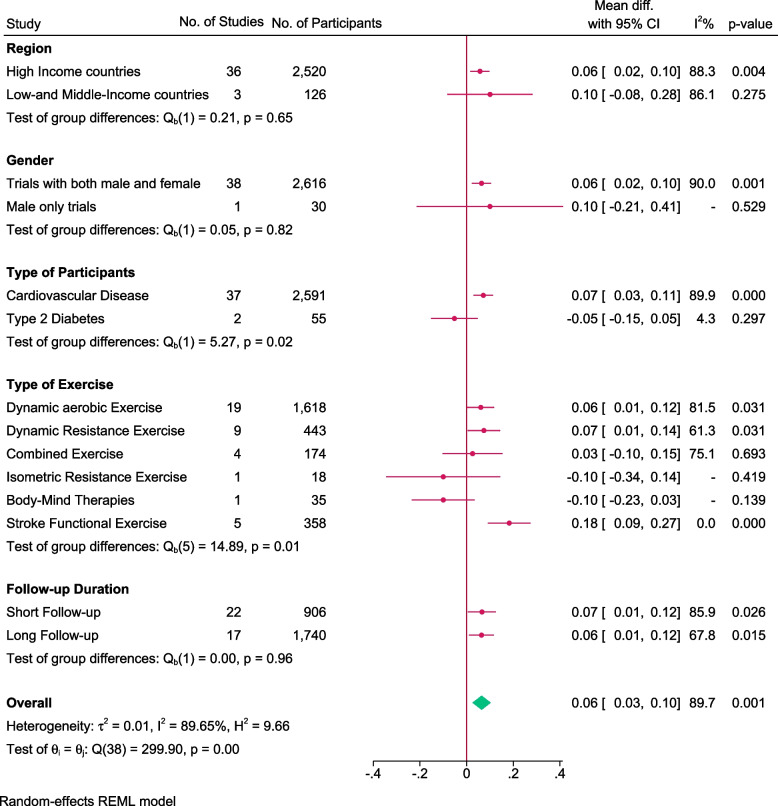
Forest plot of subgroup analysis on 10MWT

Test of interaction showed evidence of difference when comparing trials randomising different types of exercise Q = 14.89; *p* < 0.05 (Fig. 10). When analysed separately, the meta-analysis showed that exercise improved 10MWT for participants following stroke functional exercise (MD: 0.18 m/s; 95% CI 0.09 to 0.27; *p* < 0.01), dynamic resistance exercise (MD: 0.07 m/s; 95%CI 0.006 to 0.14; *p* < 0.05), dynamic aerobic exercise (MD: 0.06 m/s; 95%CI 0.01 to 0.12; *p* < 0 0.05) but not for combined exercise (MD: 0.03 m/s; 95%CI -0.01 to 0.15; *p* = 0.69), body mind therapies (MD: -0.1 m/s; 95%CI -0.23 to 0.03;*p* = 0.14) nor isometric resistance exercise(MD: -0.1 m/s; 95%CI -0.34to 0.14; *p* = 0.42).

Test of interaction showed evidence of difference when comparing trials randomising different types of participants Q = 5.27; *p* < 0.05 (Fig. 10). When analysed separately, the meta-analysis showed that exercise improved 10MWT for people with cardiovascular disease (MD: 0.07 m/s; 95% 0.03 to 0.11; *p* < 0.01), but not for people with type 2 diabetes (MD: -0.05 m/s; 95% CI -0.15 to 0.05; *p* = 0.29). There were no trials including people with hypertension.

None of the remaining planned subgroup analysis showed evidence of a difference (Fig. 10 and Table S4).

### Effect of exercise on other functional outcomes

Meta-analyses of other scales namely Berg Balance Scale (MD: 2.90; 95%CI 2.01 to 3.79; p < 0.01; I^2^ = 86.3%; 36 trials), Exercise Capacity(Watt) (MD: 23.76 W; 95%CI 16.87 to 30.64; *p* < 0.01; I^2^ = 90%; 8 trials), and Exercise Capacity(Metabolic Equivalent of Task(MET)) (MD: 1.24 MET; 95%CI 0.67 to 1.82; *p* < 0.05; I^2^ = 57.2%; 6 trials) reported statistically significant improvements in functional capacity after exercise intervention, but not for TUGT scale (MD: -1.88 s; 95%CI -3.86 to 0.09; p = 0.06; I^2^ = 97.9%; 15 trials).

All meta-analyses and the corresponding figures are included in Figures S10-S13.

### Effect of exercise on body weight

**Fig. 11:**
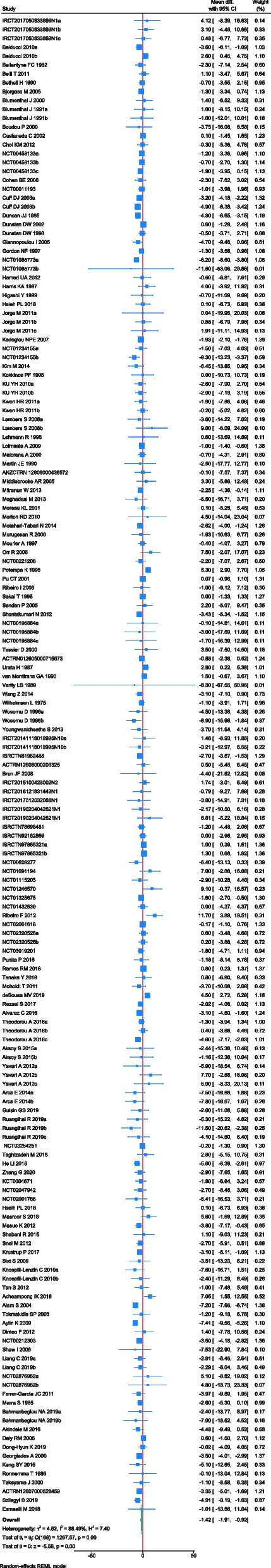
Forest plot on trials reporting body weight

**Fig. 12:**
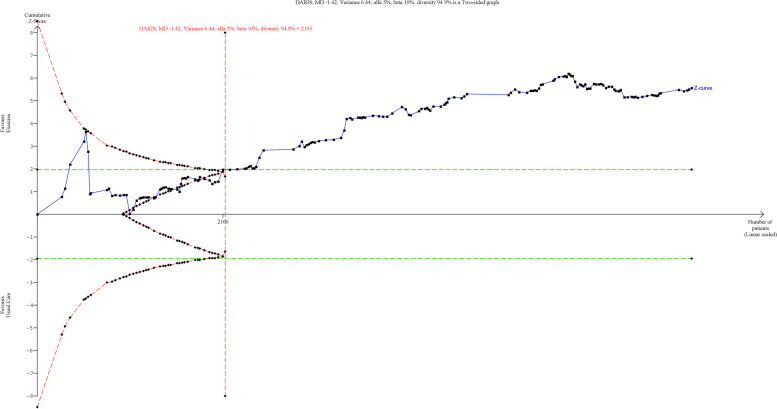
Trial sequential analysis of participants on body weight

One hundred sixty-nine trials randomising 7,535 people reported on body weight with median follow up of 3 months (IQR: 3 to 6 months). Meta-analysis showed that exercise did not significantly reduce the body weight (MD: -1.42 kg; 95%CI -1.91 to -0.92; *p* < 0.01) and the estimate was far below the pre-determined minimal clinical important difference (-5 kg). Visual inspection of forest plot (Fig. 11) and I^2^ statistics indicated substantial signs of heterogeneity which could not be resolved (I^2^ = 86.5%). Trial sequential analysis showed that there was enough information to confirm that exercise reduced body weight (Figs. 12). Funnel plot and egger’s test (*p* = 0.30) indicated no small study bias (Figure S14). We assessed this outcome result as high risk of bias (Figures S15 and S16) and the certainty of evidence as very low (Table 3).

**Fig. 13:**
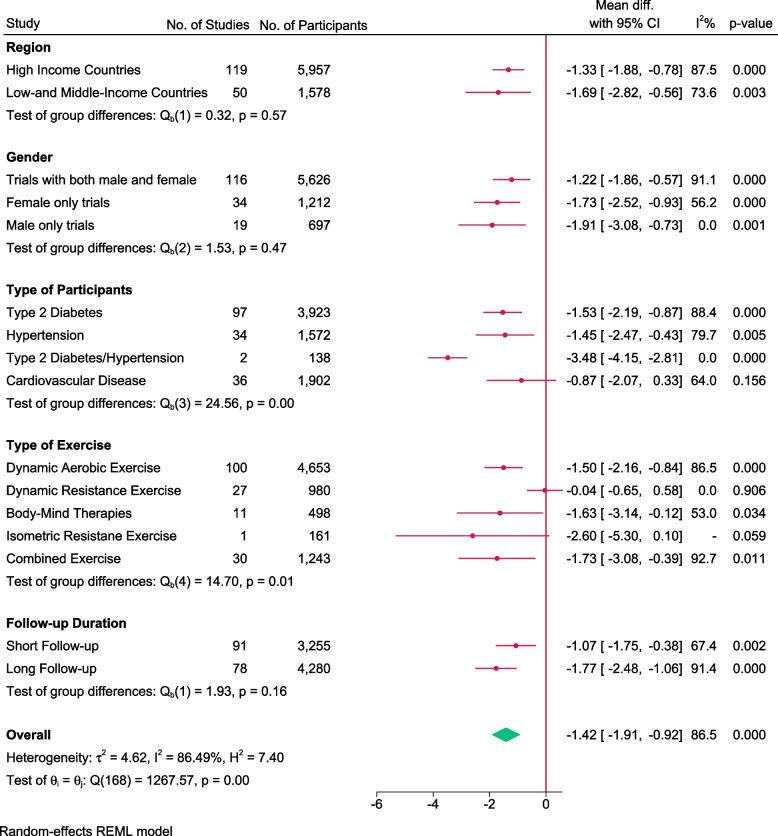
Forest plot of subgroup analysis on body weight

Test of interaction showed evidence of difference when comparing trials randomising different type of people (Q = 24.56; *p* < 0.05) (Fig. 13). When analysed separately, the meta-analysis showed exercise significantly reduced body weight for people with hypertension (MD: -1.45 kg; 95%CI -2.47 to -0.43; *p* < 0.01), people with type 2 diabetes (MD: -1.53 kg; 95%CI -2.19 to -0.87; *p* < 0.01) and people with hypertension and type 2 diabetes (MD: -3.48 kg; 95%CI -4.15 to -2.81; *p* < 0.01), but not for people with cardiovascular diseases (MD: -0.87 kg; 95%CI -2.20 to 0.35; *p* = 0.15).

Test of interaction showed evidence of difference when comparing trials randomising different type of exercise (Q = 14.70; *p* < 0.05) (Fig. 13). When analysed separately, the meta-analyses showed exercise significantly reduced body weight following combined exercise (MD: -1.73 kg; 95%CI -3.08 to -0.39; *p* < 0.05), body mind therapies (MD: -1.63 kg; 95%CI -3.14 to -0.12; *p* < 0.05), and dynamic aerobic exercise (MD: -1.50 kg; 95%CI -2.16 to -0.84; *p* < 0.01) but not for dynamic resistance exercise (MD: -0.04 kg; 95%CI -0.65 to 0.58; *p* = 0.91) and isometric resistance exercise (MD: -2.60 kg; 95% CI -5.30 to 0.10; *p* = 0.06).

Test of interaction showed evidence of difference when comparing trials randomising people with different baseline BMI category (58 trials; Q = 12.96, *p* < 0.05) (Table S5). When analysed separately, the meta-analysis showed exercise significantly reduced body weight for people with normal BMI (MD: -3.09 kg; 95%CI -5.53 to -0.64; *p* < 0.05) and people with overweight BMI (MD: -2.86 kg; 95%CI -4.24 to -1.48; *p* < 0.01) but not for obese people (MD: 0.35 kg; 95%CI -0.96 to1.65; *p* = 0.60).

None of the remaining planned subgroup analysis showed evidence of a difference (Fig. 13 and Table S5).

### Meta-regression

For the outcomes VO2max, 6MWT, 10MWT, and body weight the meta-regression on exercise led change in effect estimate, we did not observe statistically significant regression coefficients using intervention specific co-variates (length of exercise program, volume of exercise) and patient specific co-variates (type of participants, age, body mass index) (Tables S6-S9).

Additionally, A total of 27 out of 251 trials reporting VO_2_max also reported on all-cause mortality. Meta regression showed that the exercise-induced change in VO_2_max was not significantly associated with decrease in risk of all-cause mortality (RR: 0.94; 0.82 to 1.09; *p* = 0.423).

## Discussion

In this review, we analysed 355 trials assessing the effects of exercise on functional capacity and 169 trials assessing the effects of exercise on body weight. Our meta-analyses showed that exercise added to the usual care improved functional capacity as measured by VO_2_max, 6MWT and 10MWT for people with hypertension, type 2 diabetes, and/or cardiovascular disease. The effect estimates for VO_2_max and 6MWT was higher than the pre-determined minimal important difference but not for 10MWT and body weight as the effect sizes were small and may be clinically minimal. The effectiveness of improvement in functional outcomes varied with different modalities of exercise but it was notable that dynamic aerobic exercise, dynamic resistance exercise was found to consistently improve various functional capacity outcomes. Body mind therapies and inspiratory muscle training reported greater improvement for VO_2_max and 6MWT respectively compared to other forms of exercise. The observed estimates for functional capacity outcomes were independent of follow up duration, economic region, age of participants, size of trials and baseline BMI. Additionally, exercise added to usual care seemed to reduce body weight for people with hypertension and type 2 diabetes but not for people with cardiovascular disease and the reduction was higher for combined exercise and people with normal or overweight BMI but not for obese individual. However, it is important to acknowledge that the very low certainty of evidence underscores careful interpretation of the summarised evidence.

### Effect of exercise in functional outcomes

Exercise-induced improvement in VO_2_max and 6MWT was higher than the predetermined level of minimal important difference indicating clinical significance of the reported estimate. The VO_2_max reported here is lower than previous meta-analysis that reported increment in cardiorespiratory fitness of 3.5 ml/kg/min (≈1 MET) lowered the risk of all-cause mortality and cardiovascular disease by 13% and 15% among healthy people [43]. However, even the modest improvement in 1–2 ml/kg/min VO_2_max has been associated with lowering clinical outcomes and better cardio-respiratory fitness among people with cardiovascular disease [44, 45]. and hypertension [7].

Likewise, our reported estimate on exercise-led improvement of 6MWT is similar to another meta-analysis assessing exercise-based rehabilitation for heart failure [46]. In case of 10MWT, though statistically significant was lower than minimal important difference predetermined in this review so it may have minimal clinical relevance. However, this could also be due to the inherent limitation of minimal clinical importance difference used in our review which is based on distributional method- Cohen’s D definition i.e., SD/2 in the control group [35]. Nevertheless, the estimates reported here is still much lower than minimal clinical importance difference obtained through distributional and discriminative methods for 0.16 m/s for the 10-m walk test in stroke patients [47].

### Exercise-specific considerations

The subgroup analysis demonstrated evidence of a difference in different type of exercise on cardiorespiratory fitness measured by VO_2_max, 10MWT, and 6MWT. Higher improvement in VO_2_max was reported for body mind therapies and dynamic aerobic exercise. It is surprising that exercises like yoga and tai chi which includes both psychological and physical mechanisms may be as effective in improving cardiorespiratory fitness as dynamic aerobic exercise and even more effective than other prominent types of exercise. Though it should be noted that there were only five trials involving body mind therapies and information on the intensities of such kind of exercise were usually not mentioned in the trials hence comparison with other types of exercise could be futile. Studies have shown that dynamic aerobic exercise in general augment 10–30% of VO_2_max by increasing maximal stroke volume and arteriovenous oxygen difference [48].

Likewise, for 6MWT higher improvement was reported for inspiratory muscle training followed by dynamic aerobic exercise. However, there were only four studies assessing inspiratory muscle training so emergence of trials involving such exercise intervention will further add to our understanding of the effects of such trainings on functional capacity outcomes. Our result reported that exercise yielded greater improvement in 6MWT for obese and overweight individuals as compared to people with normal BMI. Thus, tailoring exercise interventions addressing the unique needs of obese and overweight individuals with coexisting cardiometabolic conditions can be particularly beneficial for improving functional capacity.

For 10MWT, larger improvement was reported for stroke functional exercise and patients with cardiovascular disease with majority being stroke patients. This specialized exercise regimen is specifically tailored to address the distinct functional limitations often severely experienced by stroke patient [49]. Common forms of exercise like aerobic exercise, resistance exercise and combination of both consistently reported improvement in functional capacity measures. Hence, the results reiterate that different forms of exercise can be recommended to individuals with hypertension, type 2 diabetes, and cardiovascular disease, but it should be prescribed with consideration of patient goals, preferences, and capabilities. Our results are concurrent with another meta-analysis that also showed that different forms of exercise have beneficial effect on improving functional capacity and reducing disability in patient with non-communicable diseases [50]. assessed through various functional capacity measures.

As the improvement of functional capacity differs according to type of exercise it is likely that the characteristics of exercise- frequency, intensity and duration may also explain the magnitude of change in functional capacity. However, in this review, the information on frequency, volume and intensity on exercise was sparse and our meta-regression among subset of trials where information was available did not show significant effect of length of intervention or volume of exercise on effect estimate. Existing evidence suggest that higher intensity exercise [51–53]. as compared to moderate intensity or traditional endurance training [54]. may elicit greater changes in cardiorespiratory fitness. A gradual progression to higher intensity may be more beneficial in reducing discomfort, maximizing safety and increasing adherence [53].

### Functional Outcomes and all-cause mortality

We did not find significant association between exercise induced VO_2_ max increment and all-cause mortality published by us [25]. One of the reasons could be that only 27 trials reported both VO_2_max and all-cause mortality thus making the meta-analysis underpowered to detect any association between exercise induced VO_2_max changes and all-cause mortality. Moreover, a long term change in cardiorespiratory fitness may provide us with a more significant assessment of its association with all-cause mortality [55].

### Effect of exercise on body weight

The meta-analysis showed that exercise when added to usual care seemed to reduce body weight minimally for people with hypertension and type 2 diabetes with normal or overweight BMI but not for cardiovascular disease and obese people. Empirical evidence suggests that even the modest reduction of 5–10% of initial body weight or weight loss of < -5 kg has been associated with clinically significant improvement in CVD risk factors for individuals with type 2 diabetes [21]. and adults with overweight and obese respectively [56]. while the extent of cardiovascular benefit of weight loss varies [57, 58]. This result further highlights the assertion that reducing body weight is often complex and strongly influenced by diet and genetics [23]. and exercise alone may not be sufficient [59]. One of the reasons for absence of reduced body weight especially for obese individuals with cardiovascular disease after exercise intervention could be attributed to the fact that exercise may reduce visceral body fat but at the same time increase muscle mass leading to no or insignificant loss in overall body weight [60]. Thus, prescribing exercise as a sole purpose of losing weight may not be an optimal strategy for obese patients with cardiovascular disease and may be a discouraging element in adherence to exercise regimen [61].

Like previous reviews, our result showed that the body weight reduction was more pronounced for combined exercise, body mind therapies and aerobic exercise and not for resistance and isometric exercise previous review [56]. While the comparable effect of body mind therapies presents a potential adjunct therapy to lose weight for people with hypertension, type 2 diabetes, and cardiovascular disease but the effect of such exercise as weight management strategy remains insufficiently investigated [62].

### Implications for low- and middle-income countries

Our result did not suggest evidence of difference between trials from low- and middle-income countries compared to trials from high income countries for effect of exercise on functional capacity and body weight. The consistent beneficial impact of exercise on functional capacity across different economic contexts is promising; however, it is essential to acknowledge that this finding may be influenced by the limited statistical power of the analysis. It was important to note that the evidence generated from these regions were disproportionately fewer (25%) compared to high income countries. Specifically, for VO_2_max, a mere 20% (49 out of 251) of the included trials originated from low- and middle-income countries. This review like other studies [1, 63, 64]. reiterates the need of more representative data from low-and middle-income economic region to have a better understanding of the role of exercise in different socio-economic context.

### Strengths of this review

The present review has various strengths. We followed the pre-published protocol, which was registered and published before the literature search ended [26]. We assessed the risk of bias using the Cochrane Risk of Bias version 1 tool and trial sequential analysis to control the risks of random errors. We included trials irrespective of any language, setting, or publication status. To minimise inaaccuracis in data extraction a team of five authors were involved in data extraction using standardized data extraction sheet and risk of bias assessment. We also did not identify signs of small study bias in our review. To our knowledge, the review is the first of its kind to assess the effect of all forms of exercise in people with hypertension, type 2 diabetes, or cardiovascular disease- the leading non-communicable disease on various functional capacity measures and body weight.

### Limitations of this review

The limitation of this review needs to be considered. All the trials included in this review were assessed as having high risk of bias. For instance, in many of the trials, there was a lack of sufficient description regarding the random allocation of people and the concealment procedure. Additionally, participants in most trials were aware of their allocation to the exercise or control group, or the descriptions were unclear, which could have influenced the overall impact of the intervention. Furthermore, information on lost to follow-up and reporting bias due to selective outcome reporting was generally unavailable. Most of the trials were small, predominantly with less than 100 participants. Our post-hoc subgroup analysis did not show evidence of difference for functional capacity measures and body weight based on size of trials. It was also surprising to observe that the majority of the trials did not report on baseline characteristics, especially age, medication use, and details on exercise frequency, intensity, and adherence which further limits the discussion of the results. Though our meta-regression did not show significant effect of exercise-specific and disease-specific characteristics on functional capacity and body weight, but it has to be considered that inferences was limited due to lack of trials reporting those co-variates.

Furthermore, we have pooled different types of exercise and different people which may lead to clinical heterogeneity and hence needs to be considered while interpreting the results. Our results reported high statistical heterogeneity, but this phenomenon is inevitable for meta-analyses of continuous outcomes with a large number of trials [65]. We reported the results primarily based on the random-effects model while results from the fixed effect model have been presented as sensitivity analysis. The results for VO_2_max, 6MWT and body weight for both models were comparably similar however, the results varied for 10MWT indicating significant heterogeneity (text S3). Some of the heterogeneity was explained by several planned and post-hoc subgroup analyses, however, the heterogeneity could not be fully resolved even after multivariate meta-regression. We also acknowledge that the variation of usual care between trials may have impacted the estimates of functional capacity measures and body weight reported in this review. However, our post-hoc subgroup analysis did not suggest evidence of difference for different variation in control reported in this review (usual care/ no intervention /with co-interventions).

We did not find significant differences in exercise induced improvement in functional capacity between the different groups of participants (hypertension, type 2 diabetes, cardiovascular disease). However, it is possible that duration of illness, variation in medication use, and severity of these conditions could potentially play an important role in effect of exercise on functional capacity which could not be comprehensively explored in this review and calls for further exploration in future research.

## Conclusion

In conclusion, our meta-analysis and trial sequential analysis further substantiated that adding exercise to usual care seemed to improve functional capacity and may potentially be recommended for people with hypertension, type 2 diabetes, or cardiovascular disease. Notably, dynamic aerobic and resistance exercise consistently enhanced various functional capacity outcomes while the superiority of body-mind therapies for VO_2_max and inspiratory muscle training for 6MWT calls for further investigation. Furthermore, prescribing exercise for the sole purpose of losing weight could be a potential strategy for people with hypertension and type 2 diabetes but not for cardiovascular disease. The extent of improvement in functional capacity and reduction of body weight varied with the specific exercise regimen employed thus highlighting the importance of personalised exercise prescriptions tailored to individual needs.

### Supplementary Information

**Additional file 1:** **Text S1.** Detail Search strategy.** Text S2.** Other functional capacity Berg Balance Scal.** Table S1.** Characteristics of included studies. **Text S3.** Sensitivity Analysis (Fixed Model; Inverse Variance).** Table S2.** Subgroup analysis for VO2 max for age and baseline BMI. **Table S4.** Subgroup analysis for 10MWT for age, baseline BMI and size of trials. **Table S5.** Subgroup analysis for body weight for age and baseline BMI. **Table S6.** Meta-regression on effect of exercise on VO2max. **Table S7.** Meta-regression on effect of exercise on 6MWT. **Table S8**. Meta-regression on effect of exercise on 10MWT. **Table S9.** Meta-regression on effect of exercise on body weight.

**Additional file 2:** **figure S1.** Funnel plot for trials reporting VO2max, **figure S2.** Risk of bias graph for VO2max: review authors’ judgements about each risk of bias item presented as percentages across all included studies, **figure S3.** Risk of bias summary for VO2max. **figure S4.** Funnel plot on trials reporting 6MWT, **figure S5.** Risk of bias graph for 6MWT. **figure S6.** Risk of bias summary for 6MWT. **figure S7. **Funnel plot on trials reporting 10MWT. **figure S8.** Risk of bias graph for 10MWT, **figure S9.** Risk of bias summary for 10MWT. **figure S10.** Forest plot on trials reporting berg balance scale. **figure S11.** Forest plot on trials reporting TUGT. **figure S12.** Forest plot on trials reporting exercise capacity (watt). **figure S13.** Forest plot on trials reporting exercise capacity(MET). **figure S14.** Funnel plot on trials reporting body weight. **figure S15.** Risk of bias graph for body weight. **figure S16.** Risk of bias summary for body weight

## Data Availability

All data generated or analysed during this study are included in this published article and its supplementary information files.

## Abbreviations

10MWT: 10-Meter walk test
6MWT: 6-Minute walk test
BMI: Body Mass Index
GRADE: Grading of Recommendations Assessment, Development and Evaluation
IQR: Inter-Quartile Range
Kg: Kilo gram
M: Meter
MET: Metabolic Equivalent of Task
MD: Mean Difference
ml/kg/min: Milliliter/kilogram/minute
PRISMA: Preferred Reporting Items for Systematic Reviews and Meta-Analysis
SD: Standard deviation
Sec: Seconds
VO_2_max: Maximal Oxygen Uptake
