# Supplementary material for: Effect of exercise on functional capacity and body weight for people with hypertension, type 2 diabetes, or cardiovascular disease: a systematic review with meta-analysis and trial sequential analysis
Source: BMC Sports Sci Med Rehabil. 2024 Feb 7;16:38. doi: 10.1186/s13102-024-00829-1 (PMC10848448; doi:10.1186/s13102-024-00829-1)
Supplement: Supplementary file 1 — Additional file 1: Text S1. Detail Search strategy. Text S2. Other functional capacity Berg Balance Scal. Table S1. Characteristics of included studies. Text S3. Sensitivity Analysis (Fixed Model; Inverse Variance). Table S2. Subgroup analysis for VO2 max for age and baseline BMI. Table S4. Subgroup analysis for 10MWT for age, baseline BMI and size of trials. Table S5. Subgroup analysis for body weight for age and baseline BMI. Table S6. Meta-regression on effect of exercise on VO2max. Table S7. Meta-regression on effect of exercise on 6MWT. Table S8. Meta-regression on effect of exercise on 10MWT. Table S9. Meta-regression on effect of exercise on body weight. [file 13102_2024_829_MOESM1_ESM.pdf]

Appendix 1

Effects of adding exercise to usual care on functional capacity and body weight in people with hypertension, type 2 diabetes or cardiovascular disease: a systematic review with meta-analysis and trial sequential analysis

Text S1: Detail Search strategy

MEDLINE assessed via OVID

Cardiovascular diseases

#1 exp Cardiovascular Disease/
#2 exp Coronary Disease/
#3 exp Myocardial Ischemia/
#4 exp Heart Disease/
#5 exp Acute Coronary Syndrome/
#6 exp Angina Pectoris/
#7 exp Myocardial Infarction/
#8 (isch?emi* adj heart).tw.
#9 (myocard* adj (infarct* or re?vascular* or ischemi* or ischaem*)).tw.
#10 (coronary adj disease*).tw.
#11 ((coronary or cardiovascular or ischemic) adj event*).tw.
#12 (heart adj (disease* or attack* or infarct*)).tw.
#13 (cardiac adj disease).tw.
#14 (morbid adj (heart* or cardiovascula* or coronary* or isch?em* or myocard*)).tw.
#15 angina or MI.tw.
#16 chd or cad.tw.
#17 exp Stroke/
#18 (stroke or stokes or cerebrovasc* or cerebral vascular or apoplexy or (brain adj accident*)).tw.
#19 (brain* or cerebral or lacunar) adj infarct*).tw.
#20 or/1-19

Hypertension
#21 exp Hypertension/
#22 ((systolic or diastolic) adj blood pressure).tw
#23 21 or 22

Type 2 Diabetes
#24 exp Diabetes Mellitus, Type 2/
#25 (('adult onset' or 'type 2' or 'type ii' or 'non-insulin dependent' or 'noninsulin dependent' or 'insulin independent') adj diabet*).tw
#26 24 or 25
#27 20 or 23 or 26

Exercise
#28 exp Exercise/ or exp Exercise Therapy/
#29 exp Sports/
#30 exp Physical Exertion/
#31 exp Physical Fitness/
#32 exp Yoga/
#33 exp Tai Ji/
#34 (physical adj (fit* or train* or therap* or activit* or exert*)).tw.
#35 (exercise adj (intervention or program* or rehabilitation)).tw.
#36 ((aerobic or endurance or combined or resistance or isometric) adj exercise).tw.
#37 (sports or 'weight lifting').tw.
#38 or/28-37
#39 27 and 38
#40 (random* or blind* or placebo*).mp.
#41 exp animals/ not humans.sh.
#42 39 and 40

#43 42 not 41

EMBASE via OVID

Cardiovascular Disease

#1exp cardiovascular disease/

#2exp heart disease/

#3exp Coronary Disease/

#4exp heart infarction/

#5exp Myocardial Ischemia/

#6exp angina pectoris/

#7(isch?emi* adj heart).tw.

#8(myocard* adj (infarct* or re?vascular* or ischemi* or ischaem*)).tw.

#9(coronary adj disease*).tw.

#10((coronary or cardiovascular or ischemic) adj event*).tw.

#11(heart adj (disease* or attack* or infarct*)).tw.

#12(cardiac adj disease).tw.

#13(morbid adj (heart* or cardiovascula* or coronary* or isch?em* or myocard*)).tw.

#14angina.tw.

#15MI.tw.

#16CHD or CAD.tw.

#17exp Stroke/

#18(stroke or stokes or cerebrovasc* or cerebral vascular or apoplexy or (brain adj accident*)).tw.

#19(brain* or cerebral or lacunar) adj infarct*).tw.

#20or/1-19

Hypertension

#21exp hypertension/

#22(blood pressure or systolic blood pressure or diastolic blood pressure).sh

#2321 or 22

Type 2 Diabetes

#24exp non insulin dependent diabetes mellitus/

#25(('adult onset' or 'type 2' or 'type ii' or 'non-insulin dependent' or 'noninsulin dependent' or 'insulin independent') adj diabet*).tw.

#2624 or 25

#2720 or 23 or 26

Exercise

#28exp exercise/

#29exp training/

#30exp fitness/

#31exp sport/

#32exp yoga/

#33exp Tai Chi/

#34((aerobic or endurance or combined or resistance or strength or circuit weight) adj exercise).tw.

#35(physical adj (fit* or train* or therap* or activit* or exert*)).tw.

#36(sports or ‘weight lifting’).tw.

#3728 or 29 or 30 or 31 or 32 or 33 or 35 or 36

#3827 and 37

#39 limit 38 to human

#40(random* or blind* or placebo*).mp.

#41 39 and 40

CENTRAL

#1 MeSH descriptor: [Cardiovascular Diseases] explode all trees
#2 MeSH descriptor: [Myocardial Ischemia] explode all trees
#3 MeSH descriptor: [Heart Diseases] explode all trees
#4 MeSH descriptor: [Acute Coronary Syndrome] explode all trees
#5 MeSH descriptor: [Angina Pectoris] explode all trees
#6 MeSH descriptor: [Coronary Disease] explode all trees
#7 MeSH descriptor: [Myocardial Infarction] explode all trees
#8 MeSH descriptor: [Diabetes Mellitus, Type 2] explode all trees
#9 MeSH descriptor: [Hypertension] explode all trees
#10 (myocard* near (infarct* or ischemi* or revascular*)):ti,ab,kw
#11 (coronary near disease*):ti,ab,kw
#12 ((coronary or cardiovascular or ischemic) near event*):ti,ab,kw
#13 (heart near (disease* or attack or infarct*)):ti,ab,kw
#14 (cardiac near disease*):ti,ab,kw
#15 (chd or cad):ti,ab,kw
#16 (angina or MI):ti,ab,kw
#17 MeSH descriptor: [Stroke] explode all trees
#18 ((stroke or cerebrovasc* or cerebral vascular or apoplexy or brain) near accident*):ti,ab,kw
#19 ((systolic or diastolic) near blood pressure):ti,ab,kw
#20 (("adult onset" or "type 2" or "type ii" or "non-insulin dependent" or "noninsulin dependent" or "insulin independent") near diabet*):ti,ab,kw
#21 #1 or #2 or #3 or #4 or #5 or #6 or #7 or #8 or #9 or #10 or #11 or #12 or #13 or #14 or #15 or #16 or #17 or #18 or #19 or #20
#22 MeSH descriptor: [Exercise] explode all trees
#23 MeSH descriptor: [Physical Fitness] 1 tree(s) exploded
#24 MeSH descriptor: [Physical Exertion] explode all trees
#25 MeSH descriptor: [Yoga] explode all trees
#26 MeSH descriptor: [Sports] explode all trees
#27 MeSH descriptor: [Tai Ji] explode all trees
#28 (physical near (fit* or train* or therap* or activit* or exert*)):ti,ab,kw

#29 (exercise adj (intervention or program* or rehabilitation)):ti,ab,kw
#30 ((aerobic or endurance or combined or resistance or isometric) near exercise):ti,ab,kw
#31 (sports or "weight lifting"):ti,ab,kw
#32 #22 or #23 or #24 or #25 or #26 or #27 or #28 or #29 or #30 or #31

#33 #21 and #32 in Trials

BIOSIS/ Science Citation Index Expanded on Web of Science

#1 (TI= ("Cardiovascular disease*" OR "myocard* infarct*" OR "heart disease" OR "heart attack" OR "heart attack" OR "cardiac disease*" OR "coronary disease*" OR stroke OR

angina OR MI OR chd OR cad OR "myocard* ischemi*" OR "acute coronary syndrome" OR hypertension OR "systolic blood pressure" OR "diastolic blood pressure" OR "type II diabetes" OR "type 2 diabetes" OR "non-insulin dependent diabetes" OR "adult onset diabetes")) AND DOCUMENT TYPES: (Article)

Indexes=SCI-EXPANDED, SSCI, A&HCI, CPCI-S, CPCI-SSH, ESCI Timespan=All years

#2 (TI=(exercise OR "physical fit*" OR "physical exertion" OR sports OR yoga OR "tai chi" OR "tai-chi" OR training OR "physical therapy" OR " physical activit*" OR "aerobic exercise" OR "resistance exercise" OR "endurance exercise" OR "combined exercise" OR "circuit weight exercise" OR "exercise therapy" OR "exercise rehabilitation" OR "exercise program")) AND DOCUMENT TYPES: (Article)

Indexes=SCI-EXPANDED, SSCI, A&HCI, CPCI-S, CPCI-SSH, ESCI Timespan=All years

#3 #1 AND #2

#4 TS=(random* OR blind* OR placebo* OR meta-analysis)

Indexes=SCI-EXPANDED, SSCI, A&HCI, CPCI-S, CPCI-SSH, ESCI Timespan=All years

#5 #3 AND #4

Text S2: Other functional capacity

Berg Balance Scale

A total of 36 trials randomising 1739 participants reported on Berg Balance Scale with median follow up of 2.75 months (IQR: 1.125 to 2.75 months). Meta-analysis showed that exercise significantly improve balance (MD=2.90; 95%CI 2.01 3.79; p=0.0000). Visual inspection of forest plot and I^2 statistics indicated substantial signs of heterogeneity which could not be resolve (I^2=86.27%). There was not enough information to conduct trial sequential analysis. Funnel plot indicated no small study bias (figure S10). We assessed this outcome result as high risk of bias.

Timed Up and Go Test (TUGT)

A total of 15 trials randomising 476 participants reported on TUGT with median follow up of 1.5 months (IQR: 1 to 3 months). Meta-analysis showed that exercise did not significantly improve functional capacity as measured by TUGT (MD=-1.88; 95%CI -3.86 0.09; p=0.062). Visual inspection of forest plot and I^2 statistics showed significant signs of heterogeneity which could not be resolve I^2=97.89%. There was not enough information to conduct trial sequential analysis. Funnel plot indicated no small study bias (figure S11). We assessed this outcome result as high risk of bias.

Exercise Capacity, W

A total of 8 trials randomising 495 participants with median follow-up of 5 months (IQR: 2 to 7.5 months). Metanalysis showed that exercise significantly improved exercise capacity (MD= 23.76 W; 95%CI 16.87 30.64; p= 0.00; I^2=90%) (figure S12).

Exercise capacity, MET

A total of 6 trials randomising 457 participants with median follow-up of 2 months (IQR: 1.75 to 2.5 months). Meta-nalaysis showed that exercise significantly improved exercise capacity (MD=1.24; 95%CI 0.67 1.82; p=0.038; I^2=57.16%) (figure S13).

There was not enough information to conduct trial sequential analysis for exercise capacity. We assessed this outcome result as high risk of bias.

Table S1: Characteristics of included studies

Trial id	Year	Region	Gender	Type of exercise	Control	Type of participants	Intervention period	Max follow-up	No. Of participants	Scale	Volinweek	Intensity
**Balducci 2010a**	2010	HICs	Both	Dynamic Aerobic Exercise	No Intervention (sedentary control)	Type II diabetes	12	12	28	VO2max,BW	NA	NA
**Balducci 2010b**	2010	HICs		Combined Exercise	No Intervention (sedentary control)	Type II diabetes	12	12	30	VO2max,BW	NA	NA
**Belardinelli R 1995**	1995	HICs	Both	Dynamic Aerobic Exercise	No Intervention (sedentary control)	Chronic Heart Failure	2	2	27	VO2max	NA	NA
**Belardinelli 2008a**	2008	HICs	Both	Dynamic Aerobic Exercise	No Intervention (sedentary control)	Chronic Heart Failure	2	2	65	VO2max	63	Vigorous
**Belardinelli 2008b**	2008	HICs	Both	Dynamic Aerobic Exercise	No Intervention (sedentary control)	Chronic Heart Failure	2	2	65	VO2max	180	Vigorous
**Belardinelli 2001**	2001	HICs	Both	Dynamic Aerobic Exercise	No Intervention (sedentary control)	Chronic Heart Failure	6	6	118	VO2max	NA	NA
**Belli T 2011**	2011	LMICs	Female	Dynamic Aerobic Exercise	No Intervention (sedentary control)	Type II diabetes	3	3	17	VO2max,BW	NA	NA
**Beniaminovitz A 2002**	2002	HICs	Both	Dynamic Aerobic Exercise	No Intervention (Relaxation)	Chronic Heart Failure	3	3	25	VO2max, 6MWT	NA	NA
**Bjorgass N 2005**	2005	HICs	Male	Dynamic Aerobic Exercise	No Intervention (sedentary control)	Type II diabetes	3	3	22	VO2max,BW	NA	NA
**Blumenthal J 1991a**	1991	HICs	Both	Dynamic Aerobic Exercise	No Intervention (sedentary control)	Mild Hypertension/Stage 1 -2 Hypertension	4	4	50	VO2max,BW	NA	NA
**Blumenthal J 1991b**	1991	HICs	Both	Dynamic Aerobic Exercise	No Intervention (sedentary control)	Mild Hypertension/Stage 1 -2 Hypertension	4	4	42	VO2max,BW	NA	NA
**Boudou P 2000**	2000	HICs	Male	Dynamic Aerobic Exercise	No Intervention (sedentary control)	Type II diabetes	2	2	16	VO2max,BW	NA	NA
**Braith RW 1999**	1999	HICs	Both	Dynamic Aerobic Exercise	No Intervention (sedentary control)	Chronic Heart Failure	4	4	19	VO2max		
**Brubaker 2009**	2009	HICs	Both	Dynamic Aerobic Exercise	Usual Care	Chronic Heart Failure	4	4	44	VO2max, 6MWT	90	Moderate
**NCT00458133 a**	2010	HICs	Both	Dynamic Aerobic Exercise	No Intervention	Type II diabetes	9	9	85	VO2max,BW	150	Moderate
**NCT00458133 b**	2010	HICs	Both	Dynamic Resistance Exercise	No Intervention	Type II diabetes	9	9	87	VO2max,BW	150	Moderate
**NCT00458133 c**	2010	HICs	Both	Combined Exercise	No Intervention	Type II diabetes	9	9	90	VO2max,BW	150	Moderate
**Cider A 2003**	2003	HICs	Both	Combined Exercise	No Intervention	Chronic Heart Failure	2	2	25	VO2max, 6MWT, exercise capacity (W)	NA	NA
**Coats AJ 1992**	1990	HICs	Both	Dynamic Aerobic Exercise	No Intervention	Chronic Heart Failure	2	2	11	VO2max	NA	NA

**de Mello 2006**	2006	LMICs	Both	Combined Exercise	No Intervention (sedentary control)	Heart Failure	4	4	22	VO2max	60	NA
**Dracup K 2007**	2007	HICs	Both	Combined Exercise	No Intervention	Heart Failure	6	6	173	VO2max, 6MWT	180	Low
**Dugmore LD 1999**	1999	HICs	Both	Dynamic Aerobic Exercise	No Intervention	Myocardial Infarction	4	4	51	VO2max		
**Duncan JJ 1985**	1985	HICs	Both	Dynamic Aerobic Exercise	No Intervention	Hypertension	4	4	51	VO2max,BW	NA	NA
**Duncan P 2003**	2003	HICs	Both	Combined Exercise	Usual Care	Stroke	3	3	92	VO2max, 6MWT, 10MWT	180	NA
**NCT00176384**	2010	HICs	Male	Dynamic Aerobic Exercise	No Intervention	Chronic Heart Failure	3	3	34	VO2max	100	Vigorous
**Hambrecht R 2000+Erb S 2003**	2003	HICs	Male	Dynamic Aerobic Exercise	No Intervention	Chronic Heart Failure	6	6	64	VO2max	80	Vigorous
**Fletcher BJ 1994**	1994	HICs	Male	Dynamic Aerobic Exercise	No Intervention (co intervention diet)	Coronary Artery Disease	6	6	35	VO2max	100	NA
**Giallauria F 2008**	2008	HICs	Both	Dynamic Aerobic Exercise	No Intervention	Myocardial Infarction	6	6	61	VO2max	120	Vigorous
**Giannuzzi P 2003/ELVD-CHF**	2003	HICs	Female	Dynamic Aerobic Exercise	No Intervention	Chronic Heart Failure	6	6	89	VO2max, 6MWT, exercise capacity (W)	90	Vigorous
**Gielen S 2003**	2003	HICs	Both	Dynamic Aerobic Exercise	No Intervention	Chronic Heart Failure	6	6	20	VO2max		
**NCT00176319**	2012	HICs	Both	Dynamic Aerobic Exercise	No Intervention	Chronic Heart Failure	1	1	60	VO2max, exercise capacity (W)	NA	NA
**NCT00614224**	2012	HICs	Both	Dynamic Aerobic Exercise	Usual Care(Conventional Physiotherapy)	Stroke	3	3	36	VO2max, 6MWT, 10MWT, BBS	150	High
**Gottlieb S 1999**	1999	HICs	Both	Dynamic Aerobic Exercise	Usual Care	Congestive Heart Failure	6	6	25	VO2max, 6MWT	NA	Moderate
**Hambrecht R 1998**	1998	HICs	Male	Dynamic Aerobic Exercise	No Intervention	Chronic Heart Failure	6	6	18	VO2max	130	Vigorous
**Hambrecht R 1995**	1995	HICs	Male	Dynamic Aerobic Exercise	No Intervention	Chronic Heart Failure	6	6	18	VO2max	130	Vigorous
**Hambrecht R 2004**	2004	HICs	Male	Dynamic Aerobic Exercise	No Intervention	Coronary Artery Disease	12	12	80	VO2max	60	NA
**Harjola VP 2006**	2006	HICs	Both	Dynamic Aerobic Exercise	No Intervention	Chronic Heart Failure	3	3	17	VO2max		
**Harris KA 1987**	1987	HICs	Male	Dynamic Resistance Exercise	No Intervention	Hypertension	2.25	2.25	26	VO2max,BW	NA	NA
**Hsieh PL 2018**	2018	HICs	Both	Dynamic Resistance Exercise	Usual Care	Type II diabetes	3	3	30	VO2max,BW	NA	NA
**NCT00891514**	2010	HICs	Both	Dynamic Aerobic Exercise	Usual Care	Stroke	6	6	53	VO2max, 6MWT, BBS	135	Moderate
**Jin H 2013**	2013	LMICs	Both	Dynamic Aerobic Exercise	No Intervention	Stroke	3	3	128	VO2max, 6MWT	NA	NA

**Jonsdottir 2006**	2006	HICs	Both	Combined Exercise	Usual Care	Chronic Heart Failure	5	5	40	VO2max	100	Moderate
**Jorge M 2011a**	2011	LMICs	Both	Dynamic Aerobic Exercise	No Intervention	Type II diabetes	3	3	16	VO2max,BW	NA	NA
**Jorge M 2011b**	2011	LMICs	Both	Dynamic Resistance Exercise	No Intervention	Type II diabetes	3	3	16	VO2max,BW	NA	NA
**Jorge M 2011c**	2011	LMICs	Both	Combined Exercise	No Intervention	Type II diabetes	3	3	16	VO2max,BW	NA	NA
**Kadoglou NPE 2007**	2007	HICs	Both	Dynamic Aerobic Exercise	No Intervention	Type II diabetes	6	6	56	VO2max,BW	NA	NA
**NCT00306176 a**	2007	HICs	Both	Dynamic Aerobic Exercise	No Intervention	Type II diabetes	8	8	50	VO2max	NA	NA
**NCT00306176 b**	2007	HICs	Both	Dynamic Aerobic Exercise	No Intervention	Type II diabetes	8	8	50	VO2max	NA	NA
**Kadoglou NPE 2012**	2012	HICs	Both	Dynamic Resistance Exercise	No Intervention	Type II diabetes	3	3	47	VO2max	NA	NA
**Keteyian SJ 1999**	1999	HICs	Both	Dynamic Aerobic Exercise	Usual Care	Chronic Heart Failure	6	6	43	VO2max, exercise capacity (W)	NA	NA
**Klecha A 2007**	2007	HICs	Both	Dynamic Aerobic Exercise	No Intervention	Chronic Heart Failure	6	6	50	VO2max	NA	NA
**Koukouvou G 2004**	2004	HICs	Male	Dynamic Aerobic Exercise	No Intervention	Chronic Heart Failure	6	6	26	VO2max	NA	NA
**Kulcu DG 2007**	2007	LMICs	Both	Dynamic Aerobic Exercise	No Intervention	Chronic Heart Failure	2	2	44	VO2max		
**Lamina S 2010**	2010	LMICs	Male	Dynamic Aerobic Exercise	No Intervention	Hypertension	2	2	357	VO2max	NA	NA
**Laoutaris ID 2011**	2011	HICs	Both	Dynamic Aerobic Exercise	No Intervention	Heart Transplantation	2.5	2.5	15	VO2max, 6MWT	135	Moderate to Vigorous
**Loimaala A 2009**	2009	HICs	Both	Combined Exercise	No Intervention	Type II diabetes	12	24	48	VO2max,BW	NA	NA
**MacKay-Lyons M 2013**	2013	HICs	Both	Dynamic Aerobic Exercise	Usual Care	Stroke	3	12	37	VO2max, 6MWT, 10MWT, BBS	300	Low to Moderate
**Maiorana A 2011**	2011	HICs	Both	Dynamic Aerobic Exercise	No Intervention	Chronic Heart Failure	3	3	17	VO2max	NA	NA
**Maiorana A 2011**	2011	HICs	Both	Dynamic Resistance Exercise	No Intervention	Chronic Heart Failure	3	3	15	VO2max	NA	NA
**Meyer K 1996**	1996	HICs	Male	Dynamic Aerobic Exercise	No Intervention	Chronic Heart Failure	0.75	0.75	18	VO2max	NA	NA
**Middlebrooke AR 2005**	2005	HICs	Both	Dynamic Aerobic Exercise	Usual Care	Dynamic Aerobic Exercise	6	6	52	VO2max,BW	NA	NA
**Moghadasi M 2013**	2013	LMICs	Male	Dynamic Aerobic Exercise	No Intervention	Dynamic Aerobic Exercise	3	3	16	VO2max	NA	NA
**Moore JL 2010**	2010	HICs	Both	Dynamic Aerobic Exercise	No Intervention	Stroke	3	3	20	VO2max, 10MWT, BBS	NA	NA
**Morton RD 2010**	2010	HICs	Both	Dynamic Aerobic Exercise	No Intervention	Type II diabetes	1.75	1.75	27	VO2max,BW	NA	NA
**Oka RK 2000**	2000	HICs	Both	Combined Exercise	Usual Care	Chronic Heart Failure	3	3	24	VO2max	NA	NA

**Okada S 2010**	2010	HICs	Both	Combined Exercise	No Intervention	Type II diabetes	3	3	38	VO2max	225	NA
**Passino C 2008**	2008	HICs	Both	Dynamic Aerobic Exercise	Usual Care	Chronic Heart Failure	9	9	90	VO2max, exercise capacity (W)	NA	NA
**Passino C 2006**	2006	HICs	Both	Dynamic Aerobic Exercise	Usual Care	Chronic Heart Failure	9	9	85	VO2max, exercise capacity (W)	90	Vigorous
**Patwala A 2009**	2009	HICs	Both	Dynamic Aerobic Exercise	No Intervention (passive range motion)	Chronic Heart Failure	6	6	50	VO2max	90	Vigorous
**Potempa K 1995**	1995	HICs	Both	Dynamic Aerobic Exercise	No Intervention (passive range motion)	Stroke	2.5	2.5	42	VO2max,BW	NA	NA
**Pu CT 2001**	2001	HICs	Female	Dynamic Resistance Exercise	No Intervention (stretching)	Chronic Heart Failure	2.5	2.5	16	VO2max, 6MWT,BW	NA	NA
**Ribeiro I 2008**	2008	LMICs	Both	Dynamic Aerobic Exercise	No Intervention (stretching)	Type II diabetes	4	4	21	VO2max,BW	NA	NA
**Roveda F 2003**	2003	LMICs	Both	Dynamic Aerobic Exercise	No Intervention (stretching)	Chronic Heart Failure	4	4	16	VO2max	NA	NA
**Sarullo FM 2006**	2006	HICs	Both	Dynamic Aerobic Exercise	No Intervention (stretching)	Chronic Heart Failure	3	3	60	VO2max	90	Vigororus
**Seki E 2003**	2003	HICs	Both	Dynamic Aerobic Exercise	Usual Care	Coronary Artery Disease	6	6	38	VO2max	NA	NA
**Senden P 2005**	2005	HICs	Both	Combined Exercise	No Intervention	Chronic Heart Failure	4.5	6.5	61	VO2max,BW	NA	NA
**Servantes DM 2012a**	2012	LMICs	Both	Dynamic Aerobic Exercise	No Intervention	Chronic Heart Failure	3	3	22	VO2max	135	Low to Moderate
**Servantes DM 2012b**	2012	LMICs	Both	Combined Exercise	No Intervention	Chronic Heart Failure	3	3	23	VO2max	NA	Low to Moderate
**Sturm B 1999**	1999	HICs	Both	Dynamic Aerobic Exercise	No Intervention	Chronic Heart Failure	3	3	23	VO2max		
**Tynni-Lenne R 1997**	1997	HICs	Female	Dynamic Resistance Exercise	No Intervention	Chronic Heart Failure	2	2	16	VO2max,6MWT,Exercise capacity(W)	NA	NA
**Verity LS 1989**	1989	HICs	Female	Dynamic Aerobic Exercise	No Intervention	Type II diabetes	4	4	10	VO2max,BW	NA	NA
**Wielenga RP 1998**	1998	HICs	Male	Dynamic Aerobic Exercise	Usual Care	Chronic Heart Failure	3	3	67	VO2max	NA	NA
**Willenheimer R 1998**	1998	HICs	Both	Dynamic Aerobic Exercise	No Intervention	Chronic Heart Failure	4	4	37	VO2max	135	Moderate
**ISRCTN16466697**	2015	LMICs	Both	Dynamic Aerobic Exercise	Usual Care	Type II diabetes	6.25	6.25	102	VO2max	90	NA
**NCT00614224**	2012	HICs	Both	Dynamic Aerobic Exercise	Usual Care(Conventional Physiotherapy)	Stroke	3	3	36	VO2max,6MWT,10MWT	150	Vigorous
**NCT00678821**	2013	HICs	Both	Dynamic Aerobic Exercise	No Intervention (Education)	Hypertension	2.5	2.5	23	VO2max,6MWT	112	Moderate
**Brun JF 2008**	2008	HICs	Both	Dynamic Aerobic Exercise	Usual Care	Type II diabetes	12	12	25	VO2max,6MWT	NA	NA
**IRCT2015100423002N2**	2020	LMICs	Both	Dynamic Aerobic Exercise	Usual Care	Type II diabetes	3	3	59	VO2max,BW	NA	NA
**IRCT2016121831443N1**	2018	LMICs		Dynamic Aerobic Exercise	Usual Care	Type II diabetes	2	2	28	VO2max,6MWT	NA	NA

**ISRCTN42524037**	2011	HICs	Both	Combined Exercise	Usual Care	Heart Failure	3	3	64	VO2max,6MWT	64	NA
**ISRCTN97865321a**	2017	HICs	Both	Combined Exercise	Usual Care	Type II diabetes	6	6	26	VO2max,BW	NA	NA
**ISRCTN97865321b**	2017	HICs	Both	Combined Exercise	Usual Care	Type II diabetes	6	6	25	VO2max,BW	NA	NA
**NCT00959660**	2016	HICs	Both	Dynamic Aerobic Exercise		Usual Care(Attention control: telephone)	4.5	4.5	92	VO2max,6MWT	180	Moderate
**Yeh G 2004**	2004	HICs	Both	Tai Chi	Usual Care(Waitlist)	Heart Failure	3	3	30	VO2max,6MWT	180	NA
**NCT00628277**	2009	HICs	Both	Dynamic Aerobic Exercise	Usual Care	Coronary Heart Disease	4	5	71	VO2max,BW	NA	NA
**NCT00837603**	2013	HICs	Both	Dynamic Aerobic Exercise	Usual Care	Heart Failure	6	6	40	VO2max	45	Vigorous
**NCT00955201a**	2019	HICs	Both	Dynamic Aerobic Exercise	Usual Care	Type II diabetes	3	3	13	VO2max	NA	Moderate to Vigorous
**NCT00955201b**	2019	HICs	Both	Dynamic Resistance Exercise	Usual Care	Type II diabetes	3	3	11	VO2max	NA	Vigorous
**NCT00955201c**	2019	HICs	Both	Combined Exercise	Usual Care	Type II diabetes	3	3	10	VO2max	NA	Moderate to Vigorous
**NCT01091194**	2012	HICs	Both	Dynamic Aerobic Exercise	Usual Care	Heart Transplant	12	60	41	VO2max,BW	NA	Vigorous
**NCT01113840**	2010	HICs	Both	Dynamic Aerobic Exercise	Usual Care	Heart Failure	4	4	46	VO2max,6MWT	NA	Low to Vigorous
**NCT01246570**	2014	HICs	Both	Dynamic Aerobic Exercise	Usual Care	Coronary Heart Disease	12	12	49	VO2max,BW	NA	Vigorous
**NCT01325675**	2016	HICs	Both	Dynamic Aerobic Exercise	Usual Care	Atrial Fibrillation	3	3	51	VO2max	NA	Vigorous
**NCT01432639**	2014	HICs	Both	Dynamic Aerobic Exercise	Usual Care	Myocardial Infarction	2	2	92	VO2max	NA	NA
**Ribeiro F 2012**	2012	HICs	Both	Dynamic Aerobic Exercise	Usual Care	Myocardial Infarction	2	2	38	VO2max	165	Moderate
**NCT01935297**	2016	HICs	Both	Combined Exercise	Usual Care	Aortic stenosis	2	12	17	VO2max,6MWT	NA	NA
**NCT02048696**	2019	HICs	Both	Combined Exercise	Usual Care	Myocardial Infarction	3	3	19	VO2max	NA	NA
**NCT02144480**	2017	HICs	Both	Dynamic Aerobic Exercise	Usual Care	Coronary Artery Disease	1	1	40	VO2max,6MWT	NA	NA
**NCT02224495**	2015	HICs	Both	Combined Exercise	Usual Care	Myocardial Infarction	2	2	175	VO2max	210	Vigorous
**NCT02283047a**	2020	HICs	Both	Dynamic Aerobic Exercise	Usual Care(Attention control: simple recommendation)	Hypertension	4	4	76	VO2max	90	Moderate
**NCT02283047b**	2020	HICs	Both	Dynamic Aerobic Exercise	Usual Care(Attention control: simple recommendation)	Hypertension	4	4	77	VO2max	90	Vigorous
**NCT02283047c**	2020	HICs	Both	Dynamic Aerobic Exercise	Usual Care(Attention control: simple recommendation)	Hypertension	4	4	78	VO2max	40	Vigorous

**NCT02288442**	2017	HICs	Both	Combined Exercise	Usual Care	Hypertension	2	2	39	VO2max,6MWT	NA	NA
**NCT02413151**	2019	HICs	Both	Dynamic Aerobic Exercise	Usual Care	Type II diabetes	6	6	37	VO2max	NA	Vigorous
**Tanaka Y 2018**	2018	HICs	Male	Dynamic Aerobic Exercise	Usual Care	Chronic Heart Failure	6	6	30	VO2max,BW		
**NCT00107068 a**	2009	HICs	Both	Dynamic Aerobic Exercise	Usual Care(sham exercise)	Stroke	3	3	16	VO2max,6MWT,10MWT	NA	NA
**NCT00107068 b**	2009	HICs	Both	Dynamic Resistance Exercise	Usual Care(sham exercise)	Stroke	3	3	16	VO2max,6MWT,10MWT	NA	NA
**NCT00107068 c**	2009	HICs	Both	Combined Exercise	Usual Care(sham exercise)	Stroke	3	3	16	VO2max,6MWT,10MWT	NA	NA
**Moholdt T 2011**	2011	HICs	Both	Dynamic Aerobic Exercise	Usual Care	Myocardial Infarction	3	3	89	VO2max,BW		
**Ezema C 2015**	2015	LMICs	Both	Dynamic Aerobic Exercise	Usual Care	Type II diabetes	2	2	54	VO2max	158	Moderate to Vigorous
**Chrysohoou C 2014**	2014	HICs	Both	Dynamic Aerobic Exercise	Usual Care	Chronic Heart Failure	3	3	72	VO2max,6MWT	135	Vigorous
**Benetti M 2010a**	2010	LMICs	Both	Dynamic Aerobic Exercise	Usual Care	Myocardial Infarction	3	3	43	VO2max	NA	NA
**Benetti M 2010b**	2010	LMICs	Both	Dynamic Aerobic Exercise	Usual Care	Myocardial Infarction	3	3	44	VO2max	NA	NA
**ISRCTN78698481**	2016	HICs	Both	Dynamic Aerobic Exercise	Usual Care	Type II diabetes	3	3	26	VO2max,BW	NA	NA
**Chou C 2019**	2019	HICs	Both	Dynamic Aerobic Exercise	Usual Care	Chronic Heart Failure	3	3	30	VO2max	36	Vigorous
**deSousa MV 2019**	2019	LMICs	Both	Dynamic Aerobic Exercise	Usual Care(Cointervention:Diet)	Type II diabetes	3	3	41	VO2max,BW	120	NA
**Rezaei S 2017**	2017	LMICs	Female	Dynamic Aerobic Exercise	No Intervention	Type II diabetes	2.5	2.5	20	VO2max	NA	NA
**ISRCTN41026907**	2012	HICs	Both	Combined Exercise	Usual Care(Home stretching)	Stroke	4.75	4.75	40	VO2max,6MWT,10MWT,BBS, TUGT	158	Moderate
**Spee RF 2016**	2016	HICs	Both	Dynamic Aerobic Exercise	Usual Care(Conventional Therapy)	Chronic Heart Failure	3	3	26	VO2max	51	Vigorous
**dos Santos M 2016**	2016	LMICs	Male	Combined Exercise	No Intervention (cointervention: Testosterone)	Chronic Heart Failure	4	4	26	VO2max	60	Moderate to Vigorous
**Hordern M 2015**	2015	HICs	Both	Dynamic Aerobic Exercise	No Intervention (cointervention: Testosterone)	Type II diabetes	36	36	223	VO2max	NA	NA
**Vinetti G 2015**	2015	HICs	Male	Combined Exercise	Usual Care	Type II diabetes	12	12	20	VO2max	140	Moderate to Vigorous
**Aksoy S 2015a**	2015	HICs	Both	Dynamic Aerobic Exercise	Usual Care	Chronic Heart Failure	2.5	2.5	22	VO2max,6MWT,BW	105	Moderate to Vigorous
**Aksoy S 2015b**	2016	HICs	Both	Dynamic Aerobic Exercise	Usual Care	Chronic Heart Failure	2.5	2.5	23	VO2max,6MWT,BW	105	Moderate to Vigorous
**Cardozo C 2015a**	2015	LMICs	Both	Dynamic Aerobic Exercise	Usual Care	Coronary Heart Disease	4	4	47	VO2max	120	Vigorous
**Cardozo C 2015b**	2015	LMICs	Both	Dynamic Aerobic Exercise	Usual Care	Coronary Heart Disease	4	4	47	VO2max	120	Moderate

**NCT02000479**	2015	HICs	Both	Combined Exercise	Usual Care	Chronic Heart Failure	3	3	22	VO2max	NA	NA
**Emerenziani G 2015**	2015	HICs	Both	Dynamic Aerobic Exercise	No Intervention	Type II diabetes	3	3	30	VO2max	NA	NA
**Yavari A 2012a**	2012	LMICs	Both	Dynamic Aerobic Exercise	Usual Care	Type II diabetes	12	12	20	VO2max,BW	45	Moderate
**Yavari A 2012b**	2012	LMICs	Both	Dynamic Resistance Exercise	Usual Care	Type II diabetes	12	12	20	VO2max,BW	NA	NA
**Yavari A 2012c**	2012	LMICs	Both	Combined Exercise	Usual Care	Type II diabetes	12	12	20	VO2max,BW	NA	NA
**Rosety-Rodriguez M 2014**	2014	HICs	Both	Dynamic Aerobic Exercise	Usual Care	Type II diabetes	3.5	3.5	60	VO2max	NA	NA
**deMeirelles LR 2014**	2014	LMICs	Both	Combined Exercise	Usual Care	Chronic Heart Failure	6	6	30	VO2max	270	Moderate
**Kaltsatou A 2014a**	2014	HICs	Male	Dynamic Aerobic Exercise	No Intervention	Chronic Heart Failure	8	8	26	VO2max,BBS	50	Moderate
**Kaltsatou A 2014b**	2014	HICs	Male	Dynamic Aerobic Exercise	No Intervention	Chronic Heart Failure	8	8	25	VO2max,BBS	180	Moderate
**PACTR201112000341237**	2013	LMICs	Both	Dynamic Aerobic Exercise	No Intervention	Hypertension	2	2	217	VO2max	158	Vigorous
**Nishitani-Yokoyama M 2019**	2019	HICs	Both	Dynamic Aerobic Exercise	Usual Care	Acute Coronary Syndrome	8	8	32	VO2max	NA	NA
**Kour H 2019**	2019	LMICs	Both	Combined Exercise	Usual Care	Type II diabetes	6	6	136	VO2max		
**NCT03546270**	2019	HICs	Female	Dynamic Aerobic Exercise	Usual Care	Hypertension	4.5	4.5	100	VO2max	120	Moderate
**Taghizadeh M 2018**	2018	HICs	Both	Dynamic Aerobic Exercise	Usual Care	Type II diabetes	2	2	20	VO2max	NA	NA
**He Li 2018**	2018	LMICs	Both	Dynamic Aerobic Exercise	Usual Care	Hypertension	3	3	40	VO2max	180	Moderate
**NCT02047942**	2019	HICs	Both	Dynamic Aerobic Exercise	Usual Care	Coronary Artery Disease	3	12	54	VO2max	135	Vigorous
**NCT02001766**	2018	HICs	Both	Dynamic Aerobic Exercise	Usual Care	Type II diabetes	2.75	2.75	32	VO2max	NA	NA
**Hseih PL 2018**	2018	HICs	Both	Dynamic Resistance Exercise	Usual Care	Type II diabetes	3	3	30	VO2max	NA	NA
**Doletsky A 2018**	2018	LMICs	Both	Dynamic Aerobic Exercise	Usual Care	Chronic Heart Failure		3	35	VO2max,6MWT	NA	Moderate
**Snel M 2012**	2012	HICs	Both	Dynamic Aerobic Exercise	Usual Care(Cointervention:very low caloric diet)	Type II diabetes	4	4	27	VO2max	180	Vigorous
**ChiCTR1800015823**	2018	LMICs	Both	Qi gong	Usual Care	Coronary Artery Disease	3	3	59	VO2max	150	Moderate
**Selig S 2004**	2004	HICs	Both	Dynamic Resistance Exercise	Usual Care	Chronic Heart Failure	3	3	33	VO2max	NA	NA
**Wood R 2006**	2006	HICs	Both	Dynamic Aerobic Exercise	No Intervention	Peripheral Artery Disease	1.5	1.5	13	VO2max	NA	NA
**Sixt S 2008**	2008	HICs	Both	Dynamic Aerobic Exercise	Usual Care	Coronary Disease	1	1	23	VO2max	NA	NA

**Piotrowicz R 2015**	2015	HICs	Both	Dynamic Aerobic Exercise	Usual Care	Chronic Heart Failure	2	2	51	VO2max, 6MWT	NA	NA
**Stolinski M 2008**	2008	HICs	Both	Dynamic Aerobic Exercise	Usual Care(Unsupervised)	Type II diabetes	6	6	17	VO2max	NA	NA
**Maria Sarullo F 2006**	2006	HICs	Both	Dynamic Aerobic Exercise	No Intervention	Chronic Heart Failure	3	3	60	VO2max	90	Vigorous
**Macko RF 2005**	2005	HICs	Both	Dynamic Aerobic Exercise	Usual Care(Conventional Therapy)	Stroke	6	6	45	VO2max, 6MWT	NA	NA
**Williams A 2007**	2007	HICs	Both	Dynamic Resistance Exercise	Usual Care	Chronic Heart Failure	2.75	2.75	11	VO2max	NA	NA
**Safiyari-Hafizi H 2016**	2016	HICs	Both	Combined Exercise	Usual Care	Chronic Heart Failure	3	3	29	VO2max, 6MWT	NA	Vigorous
**Berg-Emons R 2004**	2004	HICs	Both	Dynamic Aerobic Exercise	Usual Care	Chronic Heart Failure	3	3	34	VO2max, 6MWT	120	Moderate
**Vona M 2009a**	2009	HICs	Both	Dynamic Aerobic Exercise	No intervention	Myocardial Infarction	31	1	69	VO2max	240	Vigorous
**Vona M 2009b**	2009	HICs	Both	Dynamic Resistance Exercise	No intervention	Myocardial Infarction	1	1	70	VO2max	44	Moderate to Vigorous
**Vona M 2009c**	2009	HICs	Both	Combined Exercise	No intervention	Myocardial Infarction	1	1	70	VO2max	120	NA
**Mandic S 2008a**	2008	HICs	Both	Dynamic Aerobic Exercise	Usual Care	Chronic Heart Failure	3	3	18	VO2max	NA	Moderate
**Mandic S 2008a**	2009	HICs	Both	Dynamic Aerobic Exercise	Usual Care	Chronic Heart Failure	3	3	19	VO2max	NA	Moderate
**Zheng H 2008**	2008	LMICs	Both	Dynamic Aerobic Exercise	Usual Care	Myocardial Infarction	6	6	57	VO2max	NA	NA
**Malfatto G 2009**	2009	HICs	Both	Dynamic Aerobic Exercise	Usual Care	Chronic Heart Failure	3	3	54	VO2max	NA	NA
**Knoepfli-Lenzin C 2010a**	2010	HICs	Male	Dynamic Aerobic Exercise	No intervention	Hypertension	3	3	23	VO2max	NA	NA
**Knoepfli-Lenzin C 2010b**	2010	HICs	Male	Dynamic Aerobic Exercise	No intervention	Hypertension	3	3	24	VO2max	NA	NA
**Yamamoto A 1998**	1998	HICs	Both	Dynamic Aerobic Exercise	No intervention	Myocardial Infarction	3	3	70	VO2max	NA	NA
**Oya M 1999**	1999	HICs	Both	Dynamic Aerobic Exercise	No intervention	Myocardial Infarction	1	1	28	VO2max	NA	NA
**Pullen P 2010**	2010	HICs	Both	Yoga	Usual Care	Chronic Heart Failure	2	2	34	VO2max	120	NA
**Tan S 2012**	2012	LMICs	Both	Combined Exercise	Usual Care	Type II diabetes	6	6	30	VO2max, 6MWT	150	Moderate to Vigorous
**Legramante J 2007**	2007	HICs	Both	Dynamic Aerobic Exercise	No intervention	Myocardial Infarction	0.5	0.5	82	VO2max	180	Vigorous
**Kubo N 2004**	2004	HICs	Both	Dynamic Aerobic Exercise	Usual Care	Myocardial Infarction	3	3	44	VO2max	120	Moderate
**Mimura J 2005**	2005	HICs	Both	Dynamic Aerobic Exercise	Usual Care	Myocardial Infarction	1	1	30	VO2max	160	Vigorous
**Mameletzi D 2011**	2011	HICs	Both	Dynamic Aerobic Exercise	Usual Care	Coronary Artery Disease	7	7	20	VO2max	150	Vigorous

**Wright DJ 2002**	2002	HICs	Both	Dynamic Aerobic Exercise	Usual Care	Coronay Bypass Surgery	1.5	1.5	22	VO2max	NA	Vigorous
**Vasiliauskas D 2007**	2007	HICs	Both	Dynamic Aerobic Exercise	Usual Care	Coronary Heart Disease	6	12	154	VO2max, 6MWT,Exercise capacity (MET)	NA	NA
**Alam S 2004**	2004	HICs	Both	Dynamic Aerobic Exercise	Usual care(Unupervised)	Type II diabetes	6	6	18	VO2max	NA	NA
**Linke A 2005**	2005	HICs	Both	Dynamic Aerobic Exercise	No intervention	Chronic Heart Failure	6	6	23	VO2max	NA	NA
**Tokmakidis SP 2003**	2003	HICs	Both	Dynamic Aerobic Exercise	Usual care(Unupervised)	Coronary Artery Disease	4	8	27	VO2max	NA	NA
**Beckers P 2010a**	2010	HICs	Both	Dynamic Aerobic Exercise	Usual Care	Chronic Heart Failure	3	12	16	VO2max	NA	NA
**Beckers P 2010b**	2010	HICs	Both	Dynamic Aerobic Exercise	Usual Care	Chronic Heart Failure	3	12	19	VO2max	NA	NA
**Beckers P 2010c**	2010	HICs	Both	Dynamic Aerobic Exercise	Usual Care	Chronic Heart Failure	3	12	14	VO2max	NA	NA
**Mereles D 2006**	2006	HICs	Both	Combined Exercise	Usual Care	Hypertension	3.75	3.75	30	VO2max	NA	NA
**Pozehl B 2003**	2003	HICs	Both	Combined Exercise	Usual Care	Chronic Heart Failure	3	3	21	VO2max, 6MWT	150	Vigorous
**Bilinska M 2013**	2013	HICs	Male	Dynamic Aerobic Exercise	Usual Care	Coronay Bypass Surgery	1.5	1.5	100	VO2max		
**Dimeo F 2012**	2012	HICs	Both	Dynamic Aerobic Exercise	Usual care(Unupervised)	Hypertension	3	3	47	VO2max,BW	NA	Moderate
**Asa C 2012**	2012	HICs	Both	Dynamic Aerobic Exercise	No intervention	Chronic Heart Failure+Type II diabetes	2	2	17	VO2max, 6MWT	135	NA
**Ascione A 2012**	2012			Combined Exercise	Usual Care	Chronic Heart Failure+Type II diabetes	3	3	44	VO2max	NA	NA
**Crowther R 2008**	2008	HICs	Both	Dynamic Aerobic Exercise	Usual Care	Peripheral Artery Disease	12	12	21	VO2max	NA	NA
**Crowther R 2012**	2012	HICs	Both	Dynamic Aerobic Exercise	Usual Care	Peripheral Artery Disease	6	6	16	VO2max	NA	NA
**Guazzi M 2012**	2012	HICs	Both	Dynamic Aerobic Exercise	Usual Care	Chronic Heart Failure+Type II diabetes	6	6	28	VO2max	NA	NA
**NCT00212303**	2012	HICs	Both	Combined Exercise	Usual Care	Type II diabetes+Hypertension	6	6	114	VO2max	135	Vigorous
**NCT00218972 a**	2012	HICs	Both	Dynamic Aerobic Exercise	Usual Care	Hypertension	3	3	38	VO2max	114	Vigorous
**NCT00218972 b**	2012	HICs	Both	Dynamic Aerobic Exercise	Usual Care	Hypertension	3	3	35	VO2max	141	Vigorous
**ACTRN12609 000742279**	2012	HICs	Both	Dynamic Resistance Exercise	Usual Care	Patient with Left ventricular assist device	2	2	14	VO2max, 6MWT	NA	NA
**Edwards KM 2011**	2011	HICs	Both	Dynamic Aerobic Exercise	Usual Care	Hypertension	3	3	40	VO2max	150	Moderate

**Bronas U 2011a**	2011	HICs	Both	Isometric Resistance Exercise	Usual Care	Peripheral Artery Disease	3	3	14	VO2max	NA	NA
**Bronas U 2011b**	2011	HICs	Both	Dynamic Aerobic Exercise	Usual Care	Peripheral Artery Disease	3	3	14	VO2max	NA	NA
**Chung C 2010**	2010	HICs	Both	Dynamic Aerobic Exercise	Usual Care	Myocardial Infarction	2	2	87	VO2max	150	Low to Moderate
**Brehm M 2009**	2009	HICs	Both	Dynamic Aerobic Exercise	Usual Care	Myocardial Infarction	0.75	0.75	37	VO2max		
**Bernardi L 2007**	2007	HICs	Both	Dynamic Aerobic Exercise	Usual Care	Heart Transplant	6	6	24	VO2max	150	Vigorous
**Sabelis LW 2004**	2004	HICs	Both	Combined Exercise	Usual Care	Chronic Heart Failure	6	6	61	VO2max	NA	NA
**Hiatt WR 1996a**	1996	HICs	Both	Dynamic Aerobic Exercise	No Intervention	Peripheral Artery Disease	3	3	14	VO2max	NA	NA
**Hiatt WR 1996b**	1996	HICs	Both	Dynamic Resistance Exercise	No Intervention	Peripheral Artery Disease	3	3	12	VO2max	NA	NA
**Scordo K 1991**	1991	HICs	Fem ale	Dynamic Aerobic Exercise	Usual Care	Mitral Valve Prolapse	3	3	32	VO2max	NA	NA
**Liang C 2019a**	2019	LMICs	Both	Combined Exercise	Usual Care	Coronary Heart Disease	3	3	56	VO2max,BW	300	Vigorous
**Liang C 2019b**	2019	LMICs	Both	Tai Chi	Usual Care	Coronary Heart Disease	3	3	56	VO2max,BW	300	Vigorous
**NCT02876952 a**	2019	HICs	Both	Dynamic Aerobic Exercise	No Intervention	Myocardial Infarction	4	4	29	VO2max	80	Vigorous
**NCT02876952 b**	2019	HICs	Both	Dynamic Aerobic Exercise	No Intervention	Myocardial Infarction	4	4	26	VO2max	40	Vigorous
**Froelicher V 1985**	**1985**	HICs	Both	Dynamic Aerobic Exercise	Usual Care	Coronay Bypass Surgery	12	12	48	VO2max	135	Vigorous
**Acanfora D 2016**	2016	HICs	Both	Dynamic Resistance Exercise	No Intervention	Chronic Heart Failure	1	1	72	VO2max	360	Vigorous
**Acar S 2014**	2014	LMICs	Both	Combined Exercise	Usual Care	Type II diabetes+Hyperte nsion	3	3	16	VO2max	NA	NA
**Ahmed A 2019**	2019	LMICs	Both	Dynamic Aerobic Exercise	Usual Care	Type II diabetes+Hyperte nsion	3	3	40	VO2max	NA	NA
**Ajiboye OA 2015**	2015	LMICs	Both	Combined Exercise	Usual Care	Chronic Heart Failure	3	3	51	VO2max, 6MWT	240	Vigorous
**Anomasiri W 2005**	2005	LMICs	Male	Dynamic Aerobic Exercise	Usual Care	Coronary Artery Disease	2	2	30	VO2max		
**Dressendorfer RH 1993**	1993	HICs	Male	Dynamic Aerobic Exercise	Usual Care	Myocardial Infarction	2	2	32	VO2max, 6MWT	90	Vigorous
**Evangelista LS 2006**	2006	HICs	Both	Combined Exercise	Usual Care	Chronic Heart Failure	6	6	99	VO2max	NA	NA
**Ferrier KE 2001**	2001	HICs	Both	Dynamic Aerobic Exercise	Usual Care	Hypertension	2	2	20	VO2max, exercise capacity (W)	NA	NA
**Georgiades A 2000**	2000	HICs	Both	Dynamic Aerobic Exercise	Usual Care	Hypertension	6	6	55	VO2max	180	Vigorous
**NCT02550015**	2019	HICs	Both	Dynamic Aerobic Exercise	Usual Care	Stroke	2	12	56	VO2max	114	Vigorous

**Iellamo F 2000**	2000	LMICs	Both	Dynamic Aerobic Exercise	Usual Care	Myocardial Infarction	0.5	0.5	86	VO2max	360	Vigorous
**Palevo G 2009**	2009	HICs	Both	Dynamic Resistance Exercise	Usual Care	Chronic Heart Failure	0.5	0.5	44	VO2max,6MWT		
**Ronnemma T 1986**	1986	HICs	Both	Dynamic Aerobic Exercise	Usual Care	Type II diabetes+Hypertension	4	4	25	VO2max,BW	NA	NA
**Takeyama J 2000**	2000	HICs	Both	Dynamic Aerobic Exercise	Usual Care	Coronay Bypass Surgery	12	12	28	VO2max	NA	NA
**Wilson GA 2019**	2019	HICs	Both	Dynamic Aerobic Exercise	Usual Care	Type II diabetes+Hypertension	3	3	16	VO2max, exercise capacity (W)	NA	NA
**NCT01597960**	2019	HICs	Both	Yoga	Usual Care	Acute coronary event	3	3	41	VO2max,6MWT	NA	NA
**NCT01234155 a**	2013	HICs	Both	Dynamic Aerobic Exercise	Usual Care	Type II diabetes+Hypertension	4	4	16	VO2max,BW	NA	NA
**NCT01234155 b**	2013	HICs	Both	Dynamic Aerobic Exercise	Usual Care	Type II diabetes+Hypertension	4	4	16	VO2max,BW	NA	NA
**Sutbeyaz ST 2010a**	2010	LMICs	Both	Breathing Retraining	Usual Care	Stroke	1.5	1.5	22	VO2max	NA	NA
**Sutbeyaz ST 2010b**	2010	LMICs	Both	Inspiratory Muscle Training	Usual Care	Stroke	1.5	1.5	23	VO2max	NA	NA
**Nechwatal RM 2002a**	2002	HICs	Both	Steady state exercise	Usual Care	Chronic Heart Failure	0.75	0.75	25	VO2max,BBS	NA	NA
**Nechwatal RM 2002b**	2002	HICs	Both	Interval exercise	Usual Care	Chronic Heart Failure	0.75	0.75	25	VO2max,BBS	NA	NA
**Liu SX 2017**	2017	LMICs	Both	Combined Exercise	Usual Care	Coronary Artery Disease	3	3	63	VO2max	NA	NA
**Adamopoulos S 2013**	2013	HICs	Both	Dynamic Aerobic Exercise	Usual Care	Chronic Heart Failure	3	3	22	VO2max	NA	NA
**NCT00634296**	2009	LMICs	Both	Inspiratory Muscle Training	No Intervention(Aerobic Exercise)	Chronic Heart Failure	3	3	24	VO2max,6MWT	210	Moderate
**NCT02885077**	2019	LMICs	Both	Inspiratory Muscle Training	Usual Care(Aerobic+Resitance CPT)	Coronay Bypass Surgery	3	3	24	VO2max	60	Moderate to Vigorous
**Ascione A 2012**	2012	HICs	Both	Combined Exercise	Usual Care	Type II diabetes	3	3	44	VO2max	NA	NA
**Eder B 2010**	2010	HICs	Both	Dynamic Aerobic Exercise	Usual Care(Standard cardiac rehabilitation)	Heart Surgery	1	1	60	VO2max	90	Vigorous
**ISRCTN81952488**	2016	LMICs	Both	Dynamic Aerobic Exercise	Usual Care	Hypertension	3	3	83	VO2max,BW	135	Moderate to Vigorous
**NCT01747395**	2020	LMICs	Both	Dynamic Aerobic Exercise	Usual Care	Heart Failure	4	4	22	VO2max	180	NA
**NCT01883258 a**	2018	HICs	**Both**	Combined Exercise	Usual Care	Type II diabetes	2	2	26	VO2max	NA	NA
**NCT01883258 b**	2019	HICs	**Both**	Dynamic Aerobic Exercise	Usual Care	Type II diabetes	2	2	24	VO2max	NA	NA
**Belardinelli R 1999**	1999	HICs	Both	Dynamic Aerobic Exercise	No Intervention	Chronic Heart Failure	14	14	99	VO2max	120	Vigorous

**ACTRN12607 000227493a**	2013	HICs	Both	Dynamic Aerobic Exercise	No Intervention	Stroke	4	12	51	6MWT,10MW T	90	NA
**ACTRN12607 000227493b**	2013	HICs	Both	Dynamic Aerobic Exercise	No Intervention	Stroke	4	12	51	6MWT,10MW T	90	NA
**NCT00666744**	2011	HICs	Both	Dynamic Resistance Exercise	Usual Care	Stroke	3	3	40	6MWT,BBS	245	NA
**Butterfield JA 2008**	2008	HICs	Both	Combined Exercise	Usual Care	Chronic Heart Failure	4	4	19	6MWT	315	Moderate to Vigorous
**Eich HJ 2004**	2004	HICs	Both	Dynamic Aerobic Exercise	Usual Care	Stroke	4.5	4.5	49	6MWT,10MWT		
**Flansbjer UB 2012**	2012	HICs	Both	Dynamic Resistance Exercise	No Intervention	Chronic Stroke	2.5	48	18	6MWT,10MWT		
**Kang HK 2012**	2012	HICs	Both	Dynamic Aerobic Exercise	No Intervention	Type II diabetes	1	1	20	6MWT,10MW T	180	Vigorous
**Kim M 2014**	2014	HICs	Both	Dynamic Aerobic Exercise	Usual Care	Stroke	1	1	22	6MWT,10MWT,BW		NA
**Kobayashi N 2003**	2003	HICs	Both	Dynamic Aerobic Exercise	No Intervention	Chronic Heart Failure	3	3	28	6MWT		
**ACTRN12607 000412437**	2011	HICs	Both	Dynamic Aerobic Exercise	Usual Care	Stroke	4.5	4.5	24	6MWT,10MWT		
**Lambers S 2008a**	2008	HICs	Both	Combined Exercise	No Intervention	Type II diabetes	3	3	22	6MWT,BW	180	Moderate to Vigorous
**Lambers S 2008b**	2008	HICs	Both	Dynamic Aerobic Exercise	No Intervention	Type II diabetes	3	3	24	6MWT,BW	180	Moderate to Vigorous
**EXERT Trial/NCT008 95635**	2002	HICs	Both	Combined Exercise	No Intervention	Chronic Heart Failure	12	12	139	6MWT		Vigorous
**Nilsson BB 2008**	2008	HICs	Both	Dynamic Aerobic Exercise	Usual Care	Chronic Heart Failure	4	4	80	6MWT,Exercis e capacity(W)	120	Vigorous
**NCT00489801 / HOME-BASE**	2012	HICs	Both	Dynamic Aerobic Exercise	Usual Care	Chronic Heart Failure	12	12	36	6MWT	180	Moderate
**Orr R 2006**	2006	HICs	Both	TaiChi	No Intervention	Type II diabetes	4	4	35	6MWT,10MWT,BW		NA
**Owen A 2000**	2000	HICs	Both	Dynamic Aerobic Exercise	No Intervention	Chronic Heart Failure	3	3	24	6MWT	25	NA
**Lam P 2008**	2008	HICs	Both	TaiChi	No Intervention	Type II diabetes	6	6	46	6MWT	120	NA
**Park HJ 2011**	2011	HICs	Both	Dynamic Aerobic Exercise	No Intervention(CPT)	Stroke	1	1	25	6MWT,10MWT		
**Parnell MM 2002**	2002	HICs	Both	Dynamic Aerobic Exercise	No Intervention	Congestive Heart Failure	2	2	21	6MWT	NA	NA
**ACTRN01260 5000715673**	2007	HICs	Both	TaiChi	No Intervention	Type II diabetes	4	4	37	6MWT,10MW T,BW	120	NA
**Tynni-Lenne R 2001**	2001	HICs	Both	Dynamic Resistance Exercise	No Intervention	Chronic Heart Failure	2	2	24	6MWT,10MW T	NA	NA
**NTR1534**	2009	HICs	Both	Dynamic Aerobic Exercise	Usual Care	Stroke	6	6	242	6MWT,10MW T	180	Low to Moderate
**Xiao CM 2015**	2015	LMICs	Male	TaiChi	No Intervention	Type II diabetes	3	3	32	6MWT,BBS		NA
**Yang HC 2014**	2014	HICs	Male	Dynamic Aerobic Exercise	Usual Care (conventional Therapy)	Stroke	3	3	30	6MWT,10MWT		NA

**Yang YR 2006**	2006	HICs	Both	Dynamic Resistance Exercise	No Intervention	Stroke	1	1	48	6MWT,10MWT		NA
**NTR2704**	2012	HICs	Both	Combined Exercise	Usual Care (Cointervention: Cognitive therapy)	Stroke	6	6	68	6MWT	240	Low to Moderate
**ACTRN12610 000096055**	2015	HICs	Both	Circuit Class Therapy	Usual Care(Physiotherapy 5 days)	Stroke	1	1	173	6MWT,10MW T	900	Low to Moderate
**IRCT2016111 7030942N2**	2020	LMICs	Both	Combined Exercise	No Intervention	Type II diabetes	2	2	28	6MWT	360	Moderate
**NCT01109602**	2012	HICs	Both	Yoga	Usual Care(Waitlist)	Stroke	2	2	47	6MWT	NA	NA
**NCT01115205**	2010	HICs	Both	Dynamic Aerobic Exercise	Usual Care	Type II diabetes	4	4	41	6MWT,BW	NA	NA
**NCT01197313**	2011	HICs	Both	Dynamic Aerobic Exercise	Usual Care	Chronic Heart Failure	2	2	41	6MWT	90	Low
**NCT01467206**	2018	HICs	Both	Dynamic Aerobic Exercise	Usual Care	Stroke	18	18	380	6MWT,10MW T,BBS	45	Vigorous
**NCT01789853**	2015	HICs	Both	Dynamic Aerobic Exercise	Usual Care	Stroke	2	2	32	6MWT,10MW T	240	Moderate
**NCT02107768**	2018	HICs	Both	Dynamic Aerobic Exercise	Usual Care	Stroke	3	3	56	6MWT,10MW T,BBS	NA	NA
**NCT02655627**	2018	LMICs	Both	Combined Exercise	Usual Care(Broucher for PA)	Type II diabetes	2	2	44	6MWT	NA	NA
**NCT03538249**	2020	LMICs	Both	Dynamic Aerobic Exercise	Usual Care	Chronic Heart Failure	3	3	20	6MWT	90	Vigorous
**Gary R 2012**	2012	HICs	Both	Combined Exercise	Usual Care(Attention Control)	Chronic Heart Failure	3	3	24	6MWT		Low to Moderate
**Babu A 2019**	2019	LMICs	Both	Combined Exercise	Usual Care	Hypertension	3	3	67	6MWT	NA	NA
**Chung S 2019**	2019	HICs	Both	Task-specific lower extremity training+conventional therapy	Usual Care(Conventional therapy)	Stroke	1.25	1.25	35	6MWT,10MW T,BBS,TUGT	300	NA
**Ricca-Mallada 2017**	2017	HICs	Both	Dynamic Aerobic Exercise	Usual Care	Chronic Heart Failure	6	6	34	6MWT	180	Low to Moderate
**Kim S 2016**	2016	HICs	Both	Dynamic Aerobic Exercise	Usual Care	Stroke	1	1	20	6MWT,BBS		
**Shen D 2015**	2015	LMICs	Both	Dynamic Aerobic Exercise	Usual Care	Chronic Heart Failure	1.25	1.25	40	6MWT,BBS	NA	NA
**Borland M 2014**	2014	HICs	Both	Dynamic Aerobic Exercise	Usual Care	Chronic Heart Failure	3	3	48	6MWT	120	NA
**NCT01161329**	2017	HICs	Both	Dynamic Resistance Exercise	Usual Care	Stroke	3	15	67	6MWT,10MW T,BBS	90	Low to Moderate
**ISRCTN5057 0295**	2018	HICs	Both	Dynamic Aerobic Exercise	Usual Care(normal gait re-education)	Stroke	6	6	40	6MWT,10MW T,TUGT	NA	NA
**Zhang Y 2018**	2018	LMICs	Both	Dynamic Aerobic Exercise	Usual Care	Myocardial Infarction	12	12	130	6MWT	90	Moderate
**Teng HC 2018**	2018	HICs	Both	Dynamic Aerobic Exercise	Usual Care	Chronic Heart Failure	3	3	84	6MWT	NA	NA
**ACTRN12609 000437268**	2018	HICs	Both	Dynamic Aerobic Exercise	Usual Care	Chronic Heart Failure	6	6	132	6MWT	NA	NA

**IRCT2016111 630930N1**	2018	LMICs	Both	Combined Exercise	Usual Care	Chronic Heart Failure	3	3	48	6MWT	90	Low
**Gary R 2010a**	2010	HICs	Both	Dynamic Aerobic Exercise	Usual care	Chronic Heart Failure	6	6	31	6MWT	180	Moderate
**Gary R 2010b**	2010	HICs	Both	Dynamic Aerobic Exercise	No intervention(Cognitive Therapy)	Chronic Heart Failure	6	6	31	6MWT	180	Moderate
**NCT00733161**	2012	HICs	Both	Dynamic Aerobic Exercise	Usual care	Chronic Heart Failure	0.5	0.5	27	6MWT	105	Low
**Plevo G 2009**	2009	HICs	Both	Dynamic Resistance Exercise	Usual care	Chronic Heart Failure	2	2	16	6MWT	NA	NA
**Olawale OA 2011a**	2011	LMICs	Both	Dynamic Aerobic Exercise	Usual care	Stroke	3	3	30	6MWT,10MWT		NA
**Olawale OA 2011b**	2011	LMICs	Both	Dynamic Aerobic Exercise	Usual care	Stroke	3	3	30	6MWT,10MWT		NA
**NCT00243919 a**	2013	HICs	Both	Dynamic Aerobic Exercise	Usual care	Stroke	6	6	196	6MWT,10MW T,BBS	270	NA
**NCT00243919 b**	2013	HICs	Both	Dynamic Resistance Exercise	Usual care	Stroke	6	6	188	6MWT,10MW T,BBS	270	NA
**Gary R 2004**	2004	HICs	Fem ale	Dynamic Aerobic Exercise	Usual care	Chronic Heart Failure	3	3	32	6MWT,10MWT,BBS		NA
**Brunelli S 2019**	2019	HICs	Both	Dynamic Aerobic Exercise	Usual care	Stroke	1	1	34	6MWT	200	NA
**McGuigan MR 2001**	2001	HICs	Both	Dynamic Resistance Exercise	Usual care	Peripheral Artery Disease	4	4	20	6MWT	NA	NA
**Ozdirenc M 2004**	2004	LMICs	Both	Combined Exercise	Usual care	Type II diabetes+Hyperte nsion	0.5	0.5	44	6MWT	100	Vigorous
**NCT00214513**	2009	HICs	Both	Combined Exercise	Usual care	Chronic Heart Failure	12	14	43	6MWT,Exercis e capacity(W)	180	Vigorous
**NCT01597960**	2019	HICs	Both	Yoga	Usual care	Acute coronary event	3	3	58	6MWT	NA	NA
**ANZCTR1260 9000457246a**	2013	HICs	Both	Dynamic Resistance Exercise	Usual care	Peripheral Arterial Disease	6	6	11	6MWT	90	Low
**ANZCTR1260 9000457246b**	2013	HICs	Both	Dynamic Resistance Exercise	Usual care	Peripheral Arterial Disease	6	6	11	6MWT	90	Low
**Jung NJ 2017**	2017	HICs	Both	Inspiratory Muscle Training	Usual care	Stroke	1.5	1.5	20	6MWT	NA	NA
**Parreiras de Menezes 2019**	2019	LMICs	Both	Respiratory Muscle Training	Usual care	Stroke	3	3	38	6MWT	NA	NA
**ACTRN12616 001204437**	found a thesis not a paper	HICs	Both	Tai Chi	Usual care	Coronary Heart Disease/Hyperten sion	6	6	102	6MWT	240	Moderate
**Szilagyi B 2019**	2019	HICs	Both	Combined Exercise	Usual care	Type II diabetes	6	6	208	6MWT,BW		NA
**Kwakkel G 2001a**	2001	HICs	Both	Upper limb training	Usual care	Stroke	5	13	52	10MWT	150	NA
**Kwakkel G 2001b**	2001	HICs	Both	Upper limb training	Usual care	Stroke	5	13	49	10MWT	150	NA
**Blae M 2008**	2008	HICs	Both	Isometric Resistance Exercise	Usual care	Stroke	1	1	18	10MWT	NA	NA

**Kim CM 2001**	2001	HICs	Both	Dynamic Resistance Exercise	Usual care	Stroke	1.5	1.5	20	10MWT	NA	NA
**Oullette MM 2004**	2004	HICs	Both	Dynamic Resistance Exercise	Usual care	Stroke	3	3	42	10MWT	NA	NA
**NCT00184431**	2010	HICs	Both	Intensive motor training	Usual care	Stroke	1	6.5	62	10MWT	37.5	Moderate
**NCT03021044**	2020	HICs	Both	Dynamic Aerobic Exercise	Usual care	Myocardial Infarction	4	12	222	10MWT	60	Moderate
**Kim N 2016**	2016	HICs	Both	Dynamic Aerobic Exercise	Usual care	Stroke	1	1	17	10MWT	NA	NA
**Zhu Y 2016**	2016	LMICs	Both	modified constrained induced movement therapy	Usual care	Stroke	1	1	22	10MWT	NA	NA
**Srivastava A 2016**	2016	LMICs	Both	Dynamic Aerobic Exercise	Usual care	Stroke	1	1	40	10MWT	NA	NA
**Lee YH 2015**	2015	HICs	Both	Combined Exercise	Usual care	Stroke	4	4	26	10MWT	180	Low
**Iqbal M 2020**	2020	LMICs	Both	Dynamic Aerobic Exercise	Usual care	Stroke	1	1	64	10MWT	NA	NA
**Cha HG 2017**	2017	HICs	Both	Dynamic Resistance Exercise	Usual care	Stroke	2	2	20	10MWT	NA	NA
**Langhammer B 2007**	2007	HICs	Both	Combined Exercise	Usual care	Stroke	12	12	63	BBS	80	Vigorous
**ACTRN12610 000902099**	2014	HICs	Both	Yoga	Usual care	Stroke	2.5	2.5	22	BBS	120	Vigorous
**Shin Ji 2016**	2016	HICs	Both	Bilateral upper extremity activities	Usual care	Stroke	1	1	30	BBS	NA	NA
**Choi J 2015**	2015	HICs	Both	Task Oriented Training	Usual care	Stroke	2	2	24	BBS	150	NA
**Son S 2014**	2014	HICs	Both	Dynamic Resistance Exercise	Usual care	Stroke	1.5	1.5	28	BBS	NA	NA
**Shenoy S 2009a**	2009	LMICs	Both	Dynamic Resistance Exercise	Usual care	Type II diabetes	4	4	15	BBS	NA	Vigorous
**Shenoy S 2009b**	2010	LMICs	Both	Dynamic Aerobic Exercise	Usual care	Type II diabetes	4	4	15	BBS	90	Low
**Kunkel D 2013**	2013	HICs	Both	Dynamic Resistance Exercise	Usual care	Stroke	1	1	10	BBS	NA	NA
**Chen X 2020**	2020	LMICs	Both	Dynamic Resistance Exercise	Usual care	Stroke	2	2	180	BBS	NA	NA
**IRCT2018051 3039626N1**	2018	LMICs	Male	Dynamic Aerobic Exercise	Usual care	Stroke	1.5	1.5	36	BBS	90	Moderate
**Tripp F 2014**	2014	HICs	Both	Dynamic Aerobic Exercise	Usual care	Stroke	0.5	0.5	27	BBS	NA	NA
**Tung FL 2010**	2010	HICs	Both	Sit to stand training	Usual care	Stroke	1	1	32	BBS	NA	NA
**Yen CL 2008**	2008	HICs	Both	Dynamic Aerobic Exercise	Usual care	Stroke	1	1	14	BBS	NA	NA
**Kim Y 2015**	2015	HICs	Both	Isometric Resistance Exercise	Usual care	Stroke	1.5	1.5	20	BBS	150	NA
**Shin W 2011**	2011	HICs	Both	Combined Exercise	Usual care	Stroke	1	1	21	BBS	NA	NA
**Madhuranga P 2019**	2019	LMICs	Both	Wobble board exercise	Usual care	Stroke	1.5	1.5	29	BBS	NA	NA

**Dong-Hyun K 2019**	2019	HICs	Female	Dynamic Aerobic Exercise	Usual care	Hypertension	3	3	36	TUGT	180	Vigorous
**Qurat ul Ain 2018**	2018	LMICs	Both	Combined Exercise	Usual care	Stroke	1.5	1.5	30	TUGT	NA	NA
**Luk TH 2012**	2012	LMICs	Both	Dynamic Aerobic Exercise	Usual care	Stroke	2	2	64	Exercise Capacity(MET)	180	Vigorous
**Murugesan R 2000**	2000	LMICs	Both	Yoga	Usual Care	Hypertension	2.75	2.75	22	BW	NA	NA
**Mourier A 1997**	1997	HICs	Both	Dynamic Aerobic Exercise	No Intervention	Type II diabetes	2	2	21	BW	NA	NA
**IRCT2017050833869N1a**	2017	LMICs	Female	Aerobic exercise	No Intervention	Type II diabetes	3	3	17	BW	135	Moderate
**IRCT2017050833869N1b**	2017	LMICs	Female	Dynamic Resistance Exercise	No Intervention	Type II diabetes	3	3	17	BW	NA	Moderate
**IRCT2017050833869N1c**	2017	LMICs	Female	Combined Exercise	No Intervention	Type II diabetes	3	3	18	BW	NA	Moderate to Vigorous
**Ballantyne FC 1982**	1982	HICs	Male	Aerobic exercise	No Intervention	Myocardial Infarcation	6	6	42	BW	NA	NA
**Castaneda C 2002**	2002	HICs	Both	Dynamic Resistance Exercise	Usual Care	Type II diabetes	4	4	62	BW	135	Vigorous
**Choi KM 2012**	2012	HICs	Female	Aerobic Exercise	No Intervention	Type II diabetes	3	3	75	BW	NA	NA
**Cohen BE 2008**	2008	HICs	Both	Yoga	No Intervention	Hypertension, Diabetes	2.5	2.5	24	BW	NA	NA
**Dunstan DW 2002**	2002	HICs	Both	Dynamic Resistance Exercise	No Intervention (co intervention Weight Loss)	Type II diabetes	6	6	29	BW	NA	NA
**Dunstan DW 1998**	1998	HICs	Both	Dynamic Resistance Exercise	No Intervention	Type II diabetes	2	2	21	BW	NA	NA
**Hameed UA 2012**	2012	LMICs	Both	Dynamic Resistance Exercise	No Intervention	Type II diabetes		2	48	BW	NA	NA
**Higashi Y 1999**	1999	HICs	Both	Dynamic Aerobic Exercise	No Intervention (sedentary control)	Hypertension	3	3	27	BW	NA	NA
**KU YH 2010a**	2010	HICs	Female	Dynamic Aerobic Exercise	No Intervention	Type II diabetes	3	3	23	BW	NA	NA
**KU YH 2010b**	2010	HICs	Female	Dynamic Resistance Exercise	No Intervention	Type II diabetes	3	3	21	BW	NA	NA
**Lehmann R 1995**	1995	HICs	Both	Dynamic Aerobic Exercise	No Intervention	Type II diabetes	3	3	29	BW	NA	NA
**Martin JE 1990**	1990	HICs	Male	Dynamic Aerobic Exercise	No Intervention	Hypertension	3	3	43	BW	NA	NA
**ANZCTRN 12606000436572**	2013	HICs	Both	Dynamic Resistance Exercise	No Intervention	Type II diabetes	12	12	86	BW	NA	Vigorous
**Mitranun W 2013**	2013	LMICs	Both	Dynamic Aerobic Exercise	No Intervention	Type II diabetes	3	3	43	BW	NA	NA
**Moreau KL 2001**	2001	HICs	Female	Dynamic Aerobic Exercise	No Intervention	Hypertension	6	6	24	BW	NA	NA
**Motahari-Tabari N 2014**	2014	LMICs	Female	Dynamic Aerobic Exercise	No Intervention	Type II diabetes	2	2	50	BW	NA	NA
**NCT00221208**	2010	HICs	Both	Dynamic Resistance Exercise	No Intervention	Type II diabetes	4	4	48	BW	NA	Vigorous

**Sakai T 1998**	1998	HICs	Both	Dynamic Aerobic Exercise	No Intervention	Hypertension	1	1	35	BW	NA	NA
**NCT00195884 a**	2007	HICs	Both	Dynamic Aerobic Exercise	No Intervention	Type II diabetes	5.5	6	81	BW	135	Moderate
**NCT00195884 b**	2007	HICs	Both	Dynamic Resistance Exercise	No Intervention	Type II diabetes	5.5	6	85	BW	NA	NA
**NCT00195884 c**	2007	HICs	Both	Combined Exercise	No Intervention	Type II diabetes	5.5	6	85	BW	NA	NA
**Tessier D 2000**	2000	HICs	Both	Dynamic Aerobic Exercise	No Intervention	Type II diabetes	4	4	33	BW	NA	NA
**van Montfrans GA 1990**	1990	HICs	Both	Yoga	Usual Care	Hypertension	12	12	35	BW	NA	NA
**Wosornu D 1996a**	1996	HICs	Male	Dynamic Aerobic Exercise	No Intervention	Coronary artery bypass surgery	6	6	41	BW	NA	NA
**Wosornu D 1996b**	1996	HICs	Male	Dynamic Resistance Exercise	No Intervention	Coronary artery bypass surgery	6	6	40	BW	NA	NA
**Youngwanichsetha S 2013**	2013	LMICs	Female	Tai Chi	No Intervention	Type II diabetes	3	3	64	BW	NA	NA
**IRCT20141118019995N10a**	2019	LMICs	Female	Dynamic Aerobic Exercise	Usual Care	Type II diabetes	2.5	2.5	21	BW	75	Moderate to Vigorous
**IRCT20141118019995N10b**	2019	LMICs	Female	Comined Exercise	Usual Care	Type II diabetes	2.5	2.5	21	BW	39	NA
**ACTRN12608000206325**	2014	HICs	Both	Dynamic Resistance Exercise	No Intervention	Type II diabetes	4	4	84	BW	90	Moderate
**IRCT2017012032066N1**	2018	LMICs	Both	Dynamic Aerobic Exercise	No Intervention	Type II diabetes	3	3	24	BW	90	Moderate
**IRCT20190204042621N1a**	2019	LMICs	Both	Dynamic Resistance Exercise	No Intervention	Type II diabetes	3	3	24	BW	180	Moderate
**IRCT20190204042621N1b**	2019	LMICs	Both	Dynamic Resistance Exercise	No Intervention(cointervention :Vit D)	Type II diabetes	3	3	24	BW	180	Moderate
**Theodorou A 2016a**	2016	HICs	Both	Dynamic Aerobic Exercise	Usual Care	Coronary Artery Disease	8	8	20	BW	NA	NA
**Theodorou A 2016b**	2016	HICs	Both	Dynamic Resistance Exercise	Usual Care	Coronary Artery Disease	8	8	16	BW	NA	NA
**Theodorou A 2016c**	2016	HICs	Both	Combined Exercise	Usual Care	Coronary Artery Disease	8	8	20	BW	NA	NA
**Arca E 2014a**	2014	LMICs	Female	Dynamic Aerobic Exercise	No Intervention	Hypertension	3	3	33	BW	150	Moderate
**Arca E 2014b**	2014	LMICs	Female	Dynamic Aerobic Exercise	No Intervention	Hypertension	3	3	33	BW	150	Moderate
**Gulsin GS 2019**	2019			Dynamic Aerobic Exercise	Usual Care	Type II diabetes	3	3	52	BW	150	Moderate
**Ruangthai R 2019a**	2019	LMICs	Both	Dynamic Aerobic Exercise	Usual Care	Type II diabetes	3	6	16	BW	180	Moderate to Vigorous
**Ruangthai R 2019b**	2019	LMICs	Both	Dynamic Resistance Exercise	Usual Care	Type II diabetes	3	6	17	BW	180	Moderate to Vigorous

**Ruangthai R 2019c**	2019	LMICs	Both	Combined Exercise	Usual Care	Hypertension	3	6	17	BW	180	Moderate to Vigorous
**Zhang G 2020**	2020	LMICs	Both	Tai Chi	Usual Care	Coronary Heart Disease	3	3	30	BW	90	Moderate
**KCT0004671**	2020	HICs	Fem ale	Combined Exercise	Usual Care	Type II diabetes	3	3	35	BW	NA	Low to Moderate
**Masroor S 2018**	2018	LMICs	Fem ale	Combined Exercise	Usual Care	Hypertension	3	1	28	BW	90	Moderate to Vigorous
**Masuo K 2012**	2012	HICs	Male	Dynamic Aerobic Exercise	Usual Care	Hypertension		6	60	BW	NA	NA
**Shabani R 2015**	2015		Fem ale	Dynamic Resistance Exercise	Usual Care	Type II diabetes	1.5	1.5	20	BW		
**Krustrup P 2017**	2017	HICs	Fem ale	Dynamic Aerobic Exercise	No Intervention	Hypertension	12	12	31	BW	60	Vigorous
**Acheampong IK 2018**	2018	LMICs	Both	Combined Exercise	Usual Care	Stroke	2.5	2.5	21	BW	NA	NA
**Aylin K 2009**	2009	LMICs	Both	Combined Exercise	No Intervention	Type II diabetes	2	2	36	BW	NA	NA
**Shaw I 2006**	2006	LMICs	Male	Dynamic Resistance Exercise	No Intervention	Coronary Artery Disease	2	2	28	BW	NA	NA
**Ferrer-García JC 2011**	2011	HICs	Both	Combined Exercise	Usual Care	Type II diabetes	2	6	74	BW	NA	NA
**Bahmanbeglo u NA 2019a**	2019	LMICs	Male	Dynamic Aerobic Exercise	Usual Care	Hypertension	2	2	15	BW	NA	NA
**Bahmanbeglo u NA 2019b**	2019	LMICs	Male	Dynamic Aerobic Exercise	Usual Care	Hypertension	2	2	15	BW	NA	NA
**Akindele M 2016**	2016	LMICs	Both	Dynamic Aerobic Exercise	Usual Care	Type II diabetes	1.5	1.5	27	BW	60	NA
**Daly RM 2005**	2005	HICs	Both	Dynamic Resistance Exercise	Usual Care	Type II diabetes	6	6	29	BW	NA	NA
**ACTRN12607 000528459**	2013	HICs	Both	Tai Chi	Usual Care	Type II diabetes	3	3	40	BW	180	Moderate
**Esmaeili M 2018**	2018	LMICs	Fem ale	Combined Exercise	Usual Care	Type II diabetes	2	2	16	BW	NA	NA
**NCT00011193**	2009	HICs	Fem ale	Dynamic Aerobic Exercise	No Intervention	Hypertension	6	6	349	BW	NA	NA
**Cuff DJ 2003a**	2003	HICs	Fem ale	Dynamic Aerobic Exercise	Usual care	Type II diabetes	4	4	14	BW	NA	NA
**Cuff DJ 2003b**	2003	HICs	Fem ale	Combined Exercise	Usual care	Type II diabetes	4	4	14	BW	NA	NA
**Bethell H 1990**	1990	HICs	Male	Dynamic Aerobic Exercise	Usual care	Myocardial Infarction	3	3	193	BW	NA	NA
**Giannopoulou I 2005**	2005	HICs	Fem ale	Dynamic Aerobic Exercise	Usual Care(Cointervention:Diet	Type II diabetes	3.5	3.5	22	BW	NA	NA
**Gordon NF 1997**	1997	HICs	Both	Dynamic Aerobic Exercise	No Intervention (Diet)	Hypertension	3	3	34	BW	NA	NA
**NCT01085773 a**	2010	HICs	Both	Combined Exercise	No Intervention	Type II diabetes	4	12	34	BW	NA	NA
**NCT01085773 b**	2010	HICs	Both	Dynamic Aerobic Exercise	No Intervention	Type II diabetes	4	12	31	BW	NA	NA
**Kokkinos PF 1995**	1995	HICs	Male	Dynamic Aerobic Exercise	Usual care (Drug)	Hypertension	4	8	43	BW	NA	NA

**Kwon HR 2011a**	2011	HICs	Female	Dynamic Aerobic Exercise	No Intervention	Type II diabetes	3	3	20	BW	NA	NA
**Kwon HR 2011b**	2011	HICs	Female	Dynamic Resistance Exercise	No Intervention	Type II diabetes	3	3	19	BW	NA	NA
**Maiorana A 2000**	2000	HICs	Male	Combined Exercise	No Intervention	Chronic Heart Failure	2	2	13	BW	NA	NA
**Shantakumari N 2012**	2012	LMICs	Both	Yoga	No Intervention (medication)	Type II diabetes	3	3	100	BW	NA	NA
**Urata H 1987**	1987	HICs	Both	Dynamic Aerobic Exercise	No Intervention	Hypertension	2.5	2.5	20	BW	NA	NA
**Wang Z 2014**	2014	LMICs	Both	Dynamic Aerobic Exercise	Usual Care	Stroke	1.5	1.5	45	BW	NA	NA
**Wilhelmsen L 1975**	1975	HICs	Both	Dynamic Aerobic Exercise	No Intervention	Myocardial Infarction	3	48	286	BW	90	Vigorous
**ISRCTN92162869**	2014	HICs	Both	Dynamic Aerobic Exercise	Usual Care(Cointervention:Diet	Type II diabetes	6	12	486	BW	NA	NA
**NCT02061618**	2015	HICs	Female	Dynamic Aerobic Exercise	Usual Care(Waitlist)	Type II diabetes	2	2	23	BW	120	Moderate
**NCT02320526a**	2017	HICs	Both	Dynamic Aerobic Exercise	Usual Care	Type II diabetes	0.5	0.5	20	BW	300	NA
**NCT02320526b**	2017	HICs	Both	Dynamic Aerobic Exercise	Usual Care	Type II diabetes	0.5	0.5	20	BW	300	NA
**NCT03919201**	2020	HICs	Female	Combined Exercise	No Intervention	Hypertension	3	3	25	BW	NA	NA
**Punita P 2016**	2016	LMICs	Both	Yoga	Usual Care	Hypertension	3	3	55	BW	NA	NA
**Ramos RM 2018**	2018	LMICs	Both	Dynamic Aerobic Exercise	Usual Care	Hypertension	3	3	24	BW	NA	NA
**Alvarez C 2016**	2016	LMICs	Female	Dynamic Aerobic Exercise	Usual Care	Type II diabetes	4	4	23	BW	NA	NA
**NCT03254251**	2018	HICs	Female	Dynamic Aerobic Exercise	No Intervention	Hypertension	3	3	41	BW	NA	NA
**Marra S 1985**	1985	HICs	Both	Isometric Resistance Exercise	Usual Care	Myocardial Infarction	2	55	161	BW	NA	NA
**Kang SY 2016**	2016	HICs	Female	Combined Exercise	Usual Care	Type II diabetes	3	3	16	BW	NA	NA

BW: Body Weight, LMICs: Low-and Middle-Income Countries, HICs: High Income Countries, 6MWT: Six minute walk test, 10MWT: Ten minute walk test, NA: Not Available, BBS: Berg Balance Scale, TUGT: Time Up and Go Test

Text S3: Sensitivity Analysis (Fixed Model; Inverse Variance)

VO2max: MD:1.46 ml/kg/min; 95%CI 1.41 1.51; p=0.000; I^2 = 94.1%

6MWT: MD: 15.17 m; 95%CI 13.93 16.40; p=0.000; I^2 = 91.8%

10MWT: MD: -0.04 m/s; 95%CI -0.052 -0.037; p= 0.000; I^2 = 90.6%

Body weight: MD: -1.86 kg; 95%CI -1.98 to -1.75; p < 0.01; I^2 = 86.8%

Table S2: Subgroup analysis for VO_2 max for age and baseline BMI

Subgroups	No. of Studies	MD ml/kg/min (95% CI)	I^2 %	p-value
**Age**				
≤median age (≤59 years)	118	2.83 (2.31 to 3.36)	96.6	< 0.01
>Median age (> 59 years)	103	2.49 (2.02 to 1.97)	94.9	< 0.01
Overall: *2.67 ml/kg/min (2.32 to 3.03); Q = 0.35; p = 0.86*				
**Baseline BMI**				
Normal	12	2.86 (2.31 to 3.42)	20.6	< 0.01
Overweight	51	2.29 (1.49 to 3.09)	96.8	< 0.01
Obese	34	2.54 (1.54 to 3.53)	91.7	< 0.01
Overall: *2.41 ml/kg/min (1.85 to 2.97); Q =1.35; p = 0.51*				
**Size of trials**				
>100 participants	11	3.96 (2.25 to 5.66)	97.1	< 0.01
≤ 100 participants	240	2.65 (2.31 to 3.00)	96.5	< 0.01
**Overall:** 2.72 ml/kg/min (2.38 to 3.06); Q = 2.14; p = 0.14				
**Type of control**				
Usual Care	155	2.58 (2.15 to 3.02)	97.3	< 0.01
No Intervention	89	2.99 (2.42 to 3.55)	93.6	< 0.01
Co-intervention (as same to intervention and control group)	7	2.26 (0.40 to 4.13)	86.2	< 0.05
**Overall:** *2.72 ml/kg/min (2.38 to 3.06); Q=1.43; p=0.49*				

Table S3: Subgroup analysis for 6MWT for age and baseline BMI

Subgroups	No. of Studies	MD m (95% CI)	I^2 %	p-value
**Age**				
≤median age (≤59 years)	86	42.46 (33.51 to 51.42)	88.6	< 0.01
>Median age (> 59 years)	19	39.61 (18.97 to 60.24)	84.3	< 0.01
Overall: *2.67 m (2.32 to 3.03); Q=0.35; p=0.86*				
**Baseline BMI**				
Normal	9	20.96 (1.52 to 40.40)	10.5	< 0.05
Overweight	10	50.01 (32.34 to 67.86)	83.2	< 0.01
Obese	25	57.40 (29.74 to 85.06)	88.1	< 0.01
Overall: *46.22 m (33.21 to 59.24); Q = 6.44; p < 0.05*				
**Size of trials**				
>100 participants	14	44.52 (20.24 to 68.80)	99.0	< 0.01
≤ 100 participants	103	41.73 (34.05 to 49.40)	81.1	< 0.01
**Overall:** 42.5 m (34.95 to 50.06) Q = 0.05; p = 0.83				
**Type of control**				
Usual Care	89	43.52 (34.63 to 52.41)	41.43	< 0.01
No Intervention	25	36.39 (21.61 to 51.17)	83.80	< 0.01
Co-intervention (as same to intervention and control group)	3	56.72 (11.47 to 101.97)	41.43	= 0.19
**Overall:** 42.5 m (34.95 to 50.06); Q = 1.07; p=0.59				

Table S4: Subgroup analysis for 10MWT for age, baseline BMI and size of trials

Subgroups	No. of Studies	MD m/s (95% CI)	I^2 %	p-value
**Age**				
≤median age (≤59 years)	16	0.10 (0.05 to 0.15)	61.8	< 0.01
>Median age (> 59 years)	19	0.04 (-0.02 to 0.09)	92.3	= 0.21
Overall: *0.07 m/s (0.03 0.11); Q=2.98; p=0.08*				
**Baseline BMI**	Only 5 trials reported on baseline BMI, so the subgroup analysis was not conducted			

**Size of trials**				
>100 participants	7	0.09(-0.00 to 0.19)	93.3	= 0.06
≤ 100 participants	*32*	0.06(0.01 to 0.10)	72.3	< 0.05
Overall: *0.06 m/s (0.03 to 0.10); Q = 0.50; p = 0.48;*				
**Type of control**				
Usual Care	30	0.08 (0.04 to 0.12)	*91.38*	< 0.01
No Intervention	9	*0.02 (-0.06 to 0.09)*	*63.81*	*=0.66*
Co-intervention (as same to intervention and control group)	0	-	-	-
Overall: *0.06 m/s (0.03 to 0.10); Q = 1.93; p =0.16*				

Table S5: Subgroup analysis for body weight for age and baseline BMI

**Subgroups**	**No. of Studies**	**MD kg (95% CI)**	**I^2 %**	**p-value**
**Age**				
≤median age (≤59 years)	22	-1.88 (-2.99 to -0.77)	25.3	< 0.01
>Median age (> 59 years)	84	-1.41 (-2.14 to -0.69)	90.4	< 0.01
Overall: *-1.54 (-2.17 -0.92); Q = 0.47; p = 0.49*				
**Baseline BMI**				
Normal	8	-3.09 (-5.53 to -0.65)	0.0	< 0.05
Overweight	29	-2.97 (-4.38 to -1.57)	85.0	< 0.01
Obese	21	0.35 (-0.96 to 1.65)	74.1	=0.06
Overall: *-1.65 kg (-2.68 to -0.61); Q=12.96; p < 0.05*				
Size of trials				
>100 participants	8	-2.38 (-3.38 to -1.38)	49.6	< 0.01
≤ 100 participants	161	-1.35 (-1.88 to -0.82)	87.0	< 0.01
Overall*: -1.42 kg (-1.91 to -0.92); Q= 3.20; p = 0.07*				
**Type of control**				
Usual Care	82	-1.71 (-2.41 to -1.00)	87.08	< 0.01
No Intervention	75	-1.54 (-2.37 to -0.71)	82.19	< 0.01

Co-intervention (as same to intervention and control group)	12	-0.15 (-2.12 to 1.81)	70.27	0.88
***Overall**: -1.42 kg (-1.91 to -0.92);Q =2.13; p = 0.35*				

Table S6: Meta-regression on effect of exercise on VO_2max

	**Univariate**			**Adjusted Model**		
**Covariates**	**Coefficient**	**CI**	**Univariable significance level**	**Coefficient**	**CI**	**Significance level in final model[a]**
**Type of Exercise (ref: Aerobic Exercise)**						
Dynamic Resistance Exercise	-1.64	-2.90 to -0.37	0.01	-1.21	-2.51 to -0.08	0.06
Combined Exercise	-0.99	-1.88 to -0.100	0.03	-0.82	-1.78 to 0.13	0.09
Inspiratory Muscle training	-0.231	-4.73 to 0.088	0.06	-2.09	-4.43 to 0.25	0.08
Body-Mind Therapies	-0.38	-2.84 to 2.08	0.76	-0.14	-2.55 to 2.26	0.91
Isometric Resistance Exercise	-1.23	-6.25 to 3.78	0.63	-0.72	-5.61 to 4.16	0.77
**Type of Participants (ref: People with hypertension)**						
Type 2 Diabetes	-1.36	-2.77 to 0.05	0.06	-0.63	-2.22 to 0.96	0.44
Hypertension/Type 2 Diabetes/Cardiovascular Disease	0.04	-2.02 to 2.10	0.97	0.68	-1.57 to 2.93	0.55
Cardiovascular Disease	-1.04	-2.31 to 0.22	0.11	-0.52	-1.94 to 0.89	0.46
**Age (years)**	-0.05	-0.09 to -0.01	0.02	-0.04	-0.09 to 0.00	0.09
**BMI (kg/m^2)**	-0.04	-0.14 to 0.06	0.42			
**Length of exercise (months)**	-0.04	-0.11 to 0.03	0.29			
**Volume of exercise (min/week)**	-0.00	-0.01 to 0.01	0.89			

a= No. of observations:221; I^2: 93.9%; Wald test: 16.38; p:0.06

Table S7: Meta-regression on effect of exercise on 6MWT

	Univariate			Adjusted Model		
**Covariates**	**Coefficient**	**CI**	**Univariable significance level**	**Coefficient**	**CI**	**Significance level in final model[a]**
**Type of Exercise (ref: Aerobic Exercise)**						
Dynamic Resistance Exercise	-10.60	-39.91 to 18.70	0.48	-10.60	-39.91 to 18.70	0.48
Combined Exercise	-1.56	-19.58 to 16.45	0.86	-1.56	-19.58 to 16.45	0.86
Body-Mind Therapies	-28.33	-59.18 to 2.51	0.07	-28.33	-59.18 to 2.51	0.07
Stroke Functional Exercise	-22.90	-86.61 to 40.81	0.48	-22.90	-86.61 to 40.81	0.48
Inspiratory Muscle Training (empty)	9.81	-32.00 to 51.62	0.65	9.81	-32.00 to 51.62	0.65
**Type of Participants (ref: People with hypertension)**						
Type 2 Diabetes	-11.26	-56.93 to 34.41	0.63			
Hypertension/Type 2 Diabetes/Cardiovascular Disease	-27.38	-91.95 to 37.17	0.41			
Cardiovascular Disease	-15.21	-56.97 to 26.54	0.47			
**Age (years)**	-0.15	-0.54 to -0.24	0.45			
**BMI (kg/m^2)**	-0.29	-0.23 to 0.82	0.28			
**Length of exercise (months)**	-0.64	-2.38 to 1.10	0.47			
**Volume of exercise (min/week)**	-0.02	-0.10 to 0.06	0.68			

a=No. of observation:117; I^2:91.47; Wald test; p:0.50

Table S8: Meta-regression on effect of exercise on 10MWT

	Univariate			Adjusted Model		
**Covariates**	**Coefficient**	**CI**	**Univariable significance level**	**Coefficient**	**CI**	**Significance level in final model[a]**
**Type of Exercise (ref: Aerobic Exercise)**						
Dynamic Resistance Exercise	0.01	-0.08 to 0.11	0.75	0.01	-0.08 to 0.11	0.75
Combined Exercise	-0.04	-0.16 to 0.08	0.51	-0.04	-0.16 to 0.08	0.51
Body-Mind Therapies	-0.16	-0.38 to 0.58	0.15	-0.16	-0.38 to 0.58	0.15
Stroke Functional Exercise	0.12	-0.01 to 0.25	0.07	0.12	-0.01 to 0.25	0.07
Isometric Resistance Exercise	-0.16	-0.46 to 0.14	0.29	-0.16	-0.46 to 0.14	0.29
**Type of Participants (ref: People with CVD/stroke)**						
Type 2 Diabetes	-0.12	-0.28 to 0.03	0.13			
**Age (years)**	0.00	-0.00 to -0.00	0.74			
**BMI (kg/m2)**	0.00	-0.00 to 0.00	0.43			
**Length of exercise (months)**	0.00	-0.07 to 0.79	0.98			
**Volume of exercise (min/week)**	10 obervations only					

a=No. of observation:39; I^2: 74.7%; Wald test:7.85; p:0.16

Table S9: Meta-regression on effect of exercise on body weight

	Univariate			Adjusted Model		
**Covariates**	**Coefficient**	**CI**	**Univariable significance level**	**Coefficient**	**CI**	**Significance level[a]**
**Type of Exercise (ref: Aerobic Exercise)**						
Dynamic Resistance Exercise	1.32	-0.24 to 2.88	0.09	1.32	-0.24 to 2.88	0.09
Combined Exercise	-0.12	-1.49 to 1.25	0.86	-0.12	-1.49 to 1.25	0.86
Body-Mind Therapies	0.08	-1.97 to 2.13	0.94	0.08	-1.97 to 2.13	0.94
Isometric Resistance Exercise	-0.93	-6.33 to 4.46	0.73	-0.93	-6.33 to 4.46	0.73
**Type of Participants (ref: People with Type 2 Diabetes)**						
Hypertension	0.24	-1.10 to 1.58	0.73			
Hypertension/Type 2 Diabetes/Cardiovascular Disease	-1.45	-5.41 to 2.52	0.47			
Cardiovascular Disease	0.82	-0.56 to 2.19	0.24			
**Age (years)**	-0.00	-0.02 to -0.02	0.99			
**BMI (kg/m2)**	-0.00	-0.04 to 0.04	0.92			
**Length of exercise (months)**	-0.03	-0.08 to 0.14	0.61			
**Volume of exercise (min/week)**	-0.00	-0.02 to 0.02	0.97			

a=No. of observation:169; I2: 86.9%; Wald test: 3.23; p:0.52
