# Supplementary material for: Effect of exercise on functional capacity and body weight for people with hypertension, type 2 diabetes, or cardiovascular disease: a systematic review with meta-analysis and trial sequential analysis
Source: BMC Sports Sci Med Rehabil. 2024 Feb 7;16:38. doi: 10.1186/s13102-024-00829-1 (PMC10848448; doi:10.1186/s13102-024-00829-1)
Supplement: Supplementary file 2 — Additional file 2: figure S1. Funnel plot for trials reporting VO2max, figure S2. Risk of bias graph for VO2max: review authors’ judgements about each risk of bias item presented as percentages across all included studies, figure S3. Risk of bias summary for VO2max. figure S4. Funnel plot on trials reporting 6MWT, figure S5. Risk of bias graph for 6MWT. figure S6. Risk of bias summary for 6MWT. figure S7. Funnel plot on trials reporting 10MWT. figure S8. Risk of bias graph for 10MWT, figure S9. Risk of bias summary for 10MWT. figure S10. Forest plot on trials reporting berg balance scale. figure S11. Forest plot on trials reporting TUGT. figure S12. Forest plot on trials reporting exercise capacity (watt). figure S13. Forest plot on trials reporting exercise capacity(MET). figure S14. Funnel plot on trials reporting body weight. figure S15. Risk of bias graph for body weight. figure S16. Risk of bias summary for body weight [file 13102_2024_829_MOESM2_ESM.pdf]

Appendix 2

Supplemental figure list

figure S1: Funnel plot for trials reporting VO_2max.

figure S2: Risk of bias graph for VO_2max: review authors' judgements about each risk of bias item presented as percentages across all included studies.

figure S3: Risk of bias summary for VO_2max

figure S4: Funnel plot on trials reporting 6MWT.

figure S5: Risk of bias graph for 6MWT.

figure S6: Risk of bias summary for 6MWT.

figure S7: Funnel plot on trials reporting 10MWT.

figure S8: Risk of bias graph for 10MWT.

figure S9: Risk of bias summary for 10MWT.

figure S10: Forest plot on trials reporting berg balance scale.

figure S11: Forest plot on trials reporting TUGT

figure S12: Forest plot on trials reporting exercise capacity (watt)

figure S13: Forest plot on trials reporting exercise capacity (MET) .

figure S14: Funnel plot on trials reporting body weight.

figure S15: Risk of bias graph for body weight.

figure S16: Risk of bias summary for body weight.

figure S1: Funnel plot for trials reporting VO_2max

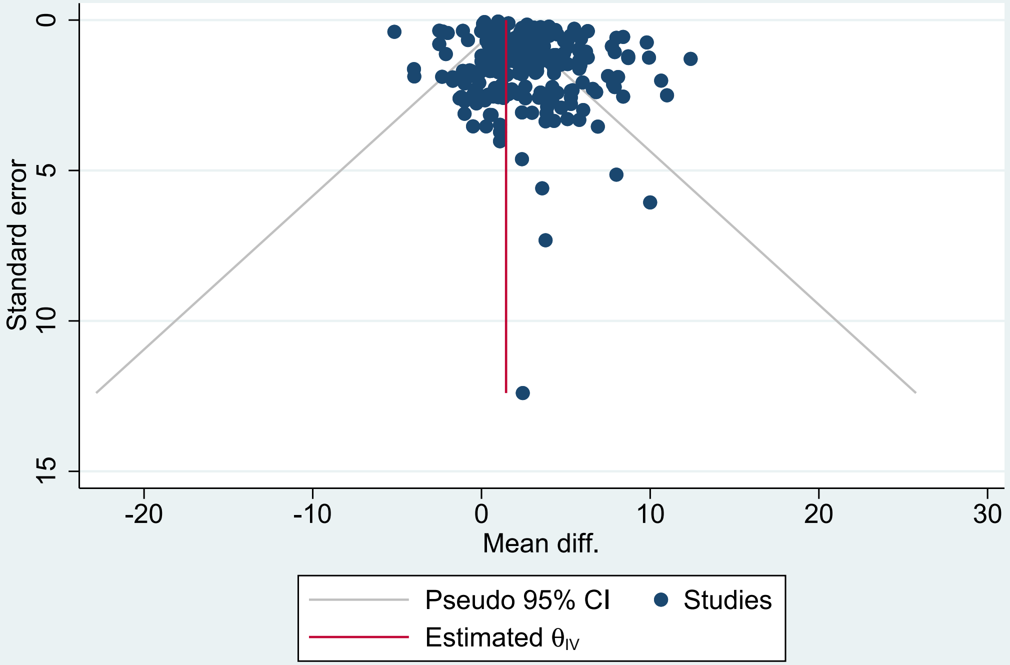

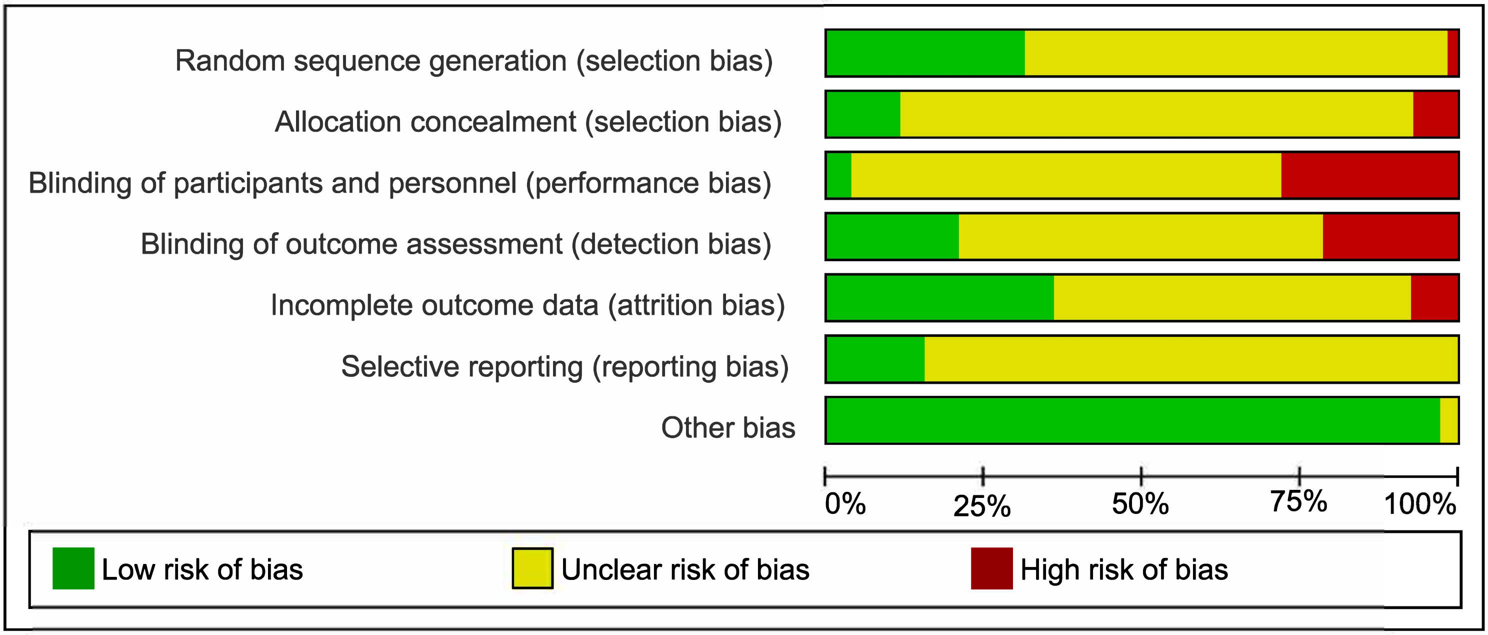

figure S2: Risk of bias graph for VO_2max: review authors' judgements about each risk of bias item presented as percentages across all included studies

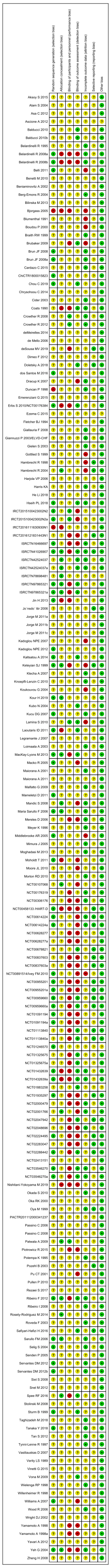

figure S3: Risk of bias summary reporting VO_2max

figure S4: Funnel plot for trials reporting 6MWT

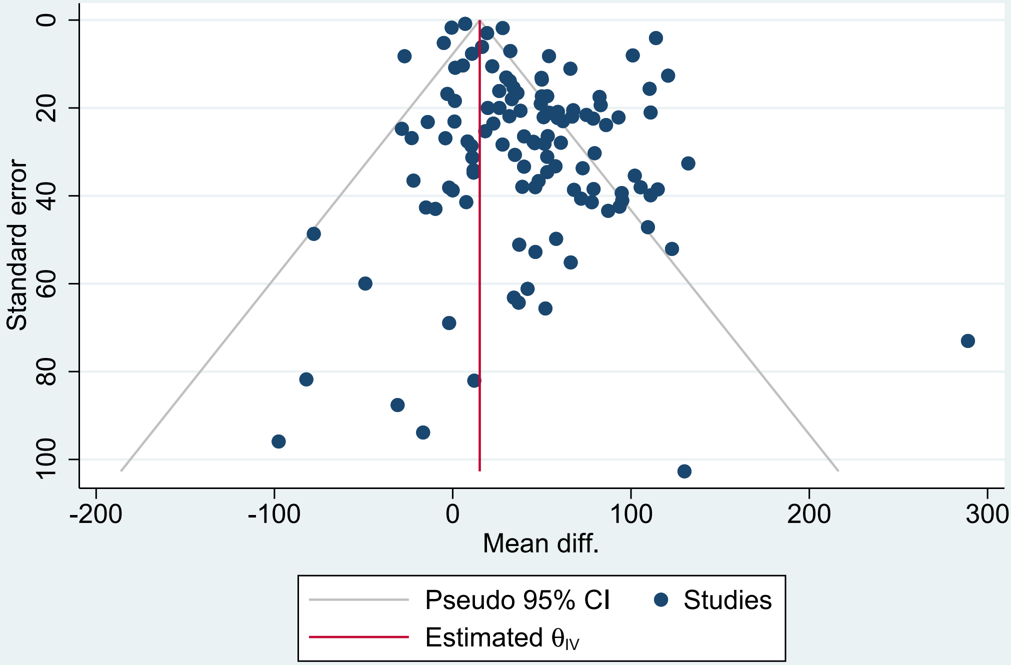

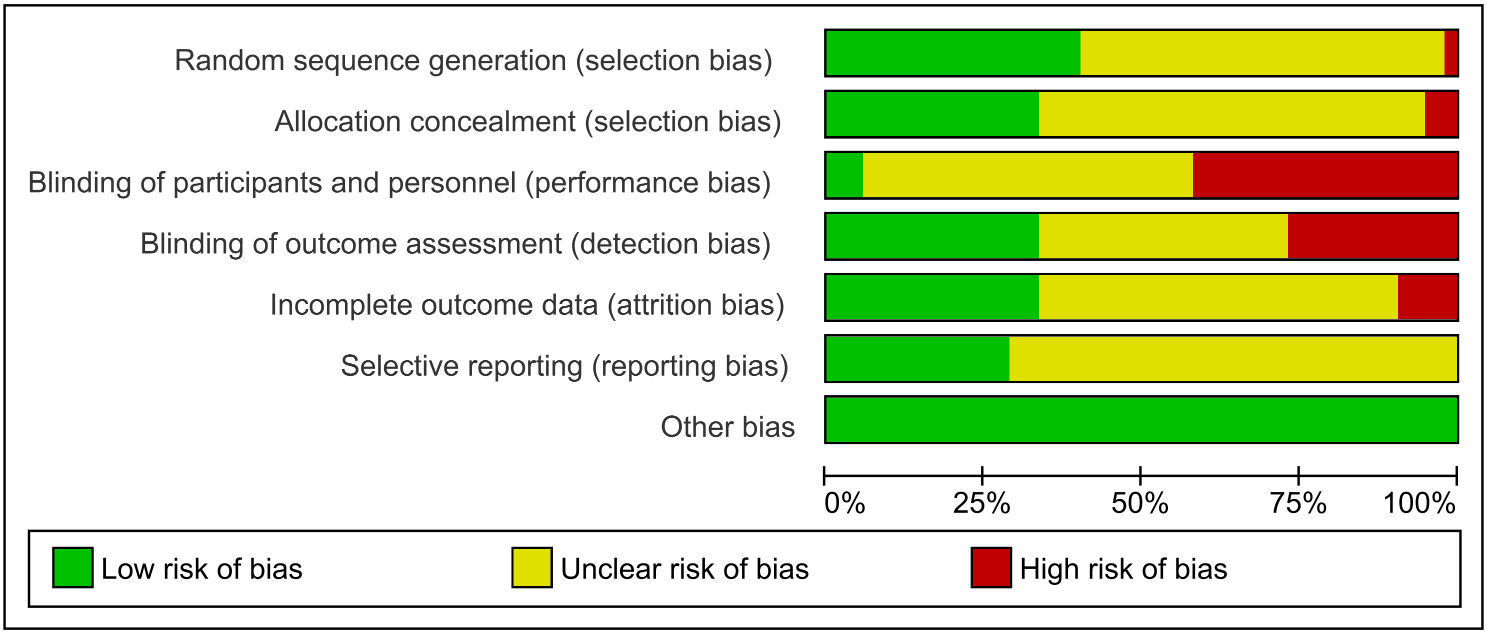

figure S5: Risk of bias graph for 6MWT outcome

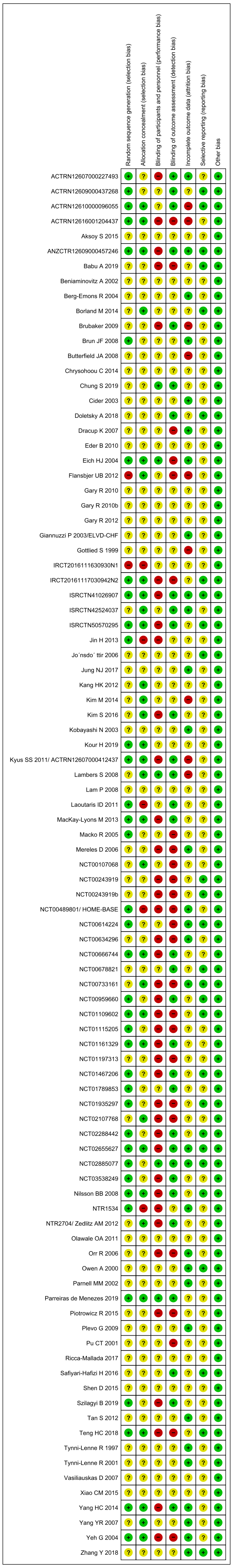

figure S6: Risk of bias summary reporting 6MWT

figure S7: Funnel plot for trials reporting 10MWT

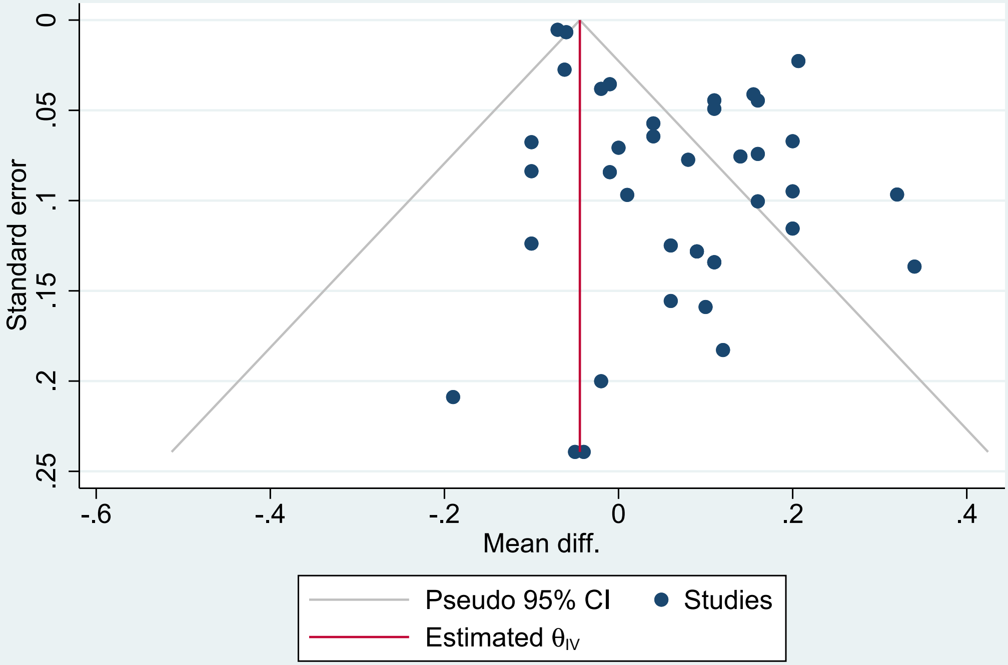

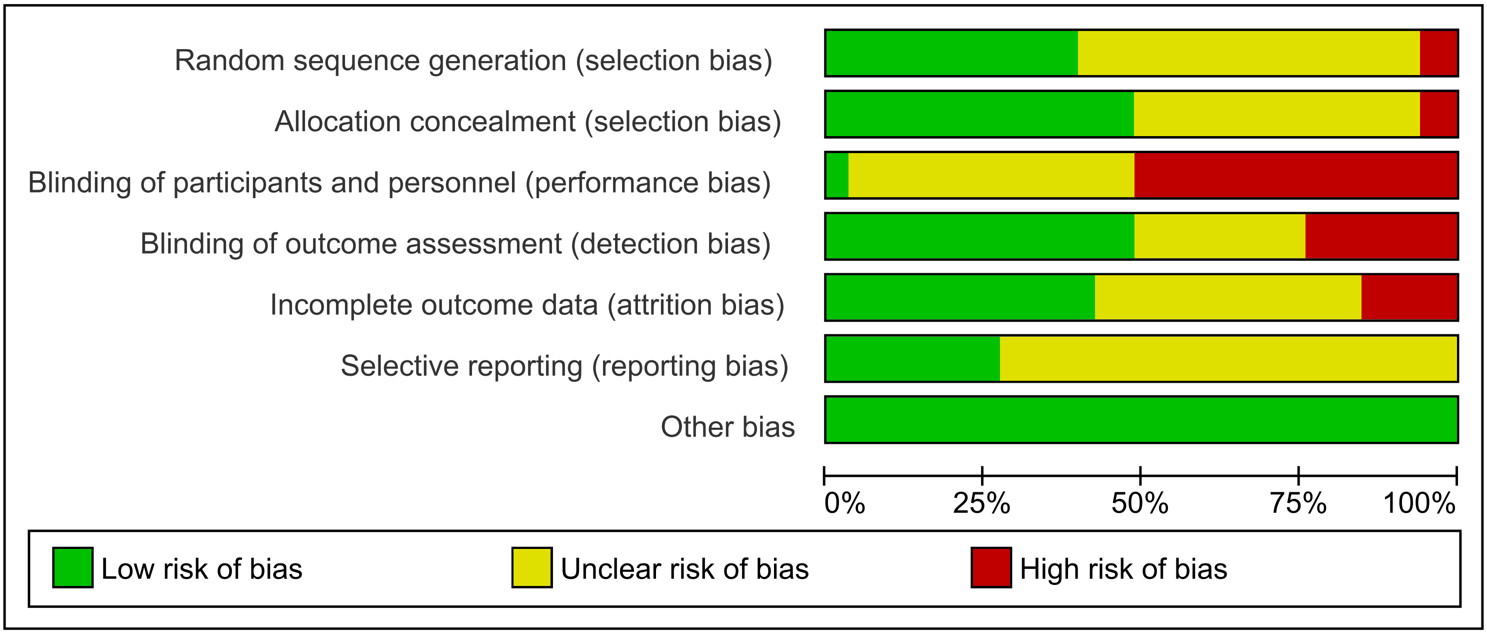

figure S8: Risk of bias graph for 10MWT

	Random sequence generation (selection bias)	Allocation concealment (selection bias)	Blinding of participants and personnel (performance bias)	Blinding of outcome assessment (detection bias)	Incomplete outcome data (attrition bias)	Selective reporting (reporting bias)	Other bias
ACTRN12607000227493	+	?	−	+	+	?	+
ACTRN12610000096055	+	+	?	+	−	+	+
ACTRN12610000096055a	+	+	?	+	−	+	+
Bale M 2008	?	?	−	−	+	?	+
Cha HG 2017	?	+	?	?	+	?	+
Duncan P 1998	−	?	?	?	+	?	+
Flansbjer UB 2012	−	+	?	−	−	?	+
Iqbal M 2020	?	+	?	?	?	?	+
ISRCTN41026907	+	+	−	+	+	+	+
Kang HK 2012	?	+	?	?	?	?	+
Kim CM 2001	?	?	+	+	+	?	+
Kim M 2014	?	+	?	?	−	?	+
Kim N 2016	?	+	?	+	?	?	+
Kwakkel G 2001	?	?	−	−	?	?	+
Kyus SS 2011/ ACTRN12607000412437	+	+	−	+	−	?	+
Lee YH 2015	?	+	−	+	?	?	+
MacKay-Lyons M 2013	+	+	−	+	?	?	+
Moore JL 2010	?	?	?	−	?	?	+
NCT00184431	+	+	−	+	+	+	+
NCT00243919	?	?	−	−	?	+	+
NCT00243919b	?	?	−	−	?	+	+
NCT00614224	+	?	?	−	+	+	+
NCT01161329	+	+	−	+	?	?	+
NCT01467206	+	?	−	+	?	+	+
NCT01789853	+	?	?	+	?	?	+
NCT03021044	?	?	−	−	?	+	+
NTR1534	+	−	−	?	+	?	+
Oullette MM 2004	?	−	−	+	+	?	+
Srivastava A 2016	?	?	−	+	?	?	+
Tynni-Lenne R 1997	?	?	?	?	+	?	+
Yang HC 2014	+	+	−	+	+	?	+
Yang YR 2007	?	+	?	?	+	?	+
Zhu Y 2016	?	?	?	?	+	?	+

figure S9: Risk of bias summary reporting 10MWT

Study	Exercise N	Exercise Mean	Exercise SD	Control N	Control Mean	Control SD	Mean diff. with 95% CI	Weight (%)
NCT00666744	20	46	14.2	20	37.6	16.2	8.40 [-1.04, 17.84]	0.76
Duncan P 2003	44	4.36	.71	48	1.7	.52	2.66 [2.41, 2.91]	5.28
NCT00614224	18	51.1	6.4	18	44.3	11.9	6.80 [0.56, 13.04]	1.47
Jin H 2013	65	47.6	3	63	47.3	4	0.30 [-0.92, 1.52]	4.82
Langhammer B 2007	32	38.3	19.5	31	48.7	11.7	-10.40 [-18.37, -2.43]	1.01
MacKay-Lyons M 2013	18	50.1	5.3	19	49.8	5.1	0.30 [-3.05, 3.65]	3.02
Moore JL 2010	10	46	14	10	47	10	-1.00 [-11.66, 9.66]	0.61
Xiao CM 2015	16	51.9	4.4	16	43.6	11.8	8.30 [2.13, 14.47]	1.49
NCT00614224	18	51.1	6.4	18	44.3	11.9	6.80 [0.56, 13.04]	1.47
ACTRN12610000902099	11	50.7	6.3	11	48.5	8	2.20 [-3.82, 8.22]	1.54
NCT00184431	30	46.9	10.6	32	45.1	11.6	1.80 [-3.74, 7.34]	1.73
NCT01789853	15	41	11	17	40	13	1.00 [-7.41, 9.41]	0.92
NCT01864382	36	34.86	18.03	32	20.62	15.35	14.24 [6.23, 22.25]	1.00
Chung S 2019	18	43.64	7.98	17	36.59	9.57	7.05 [1.23, 12.87]	1.62
ISRCTN41026907	20	55	2	20	52	5	3.00 [0.64, 5.36]	3.86
Shin Ji 2016	15	46.2	4.3	15	43.2	5	3.00 [-0.34, 6.34]	3.03
Kim S 2016	10	46.7	9.4	10	49.8	4.6	-3.10 [-9.59, 3.39]	1.39
Park G 2016	20	45.5	1.7	20	41.6	1.2	3.90 [2.99, 4.81]	5.02
Shen D 2015	10	36.8	5.6	10	34.1	4.3	2.70 [-1.68, 7.08]	2.32
Choi J 2015	12	50.5	3.11	12	47	7.5	3.50 [-1.09, 8.09]	2.19
Son S 2014	14	37.21	2.6	14	33.8	2.5	3.41 [1.52, 5.30]	4.27
Kaltsatou A 2014a	18	55.8	.4	8	52.9	1.6	2.90 [2.13, 3.67]	5.09
Kaltsatou A 2014b	17	55.4	.6	8	52.9	1.6	2.50 [1.65, 3.35]	5.05
Shenoy S 2009a	10	54	.7	5	50	2	4.00 [2.65, 5.35]	4.72
Shenoy S 2009b	10	53	1.3	5	50	2	3.00 [1.34, 4.66]	4.47
Kunkel D 2013	4	40.3	7.9	6	50	5.6	-9.70 [-18.00, -1.40]	0.94
Chen X 2020	90	37.98	3.42	90	32.68	3.015	5.30 [4.36, 6.24]	5.00
IRCT20180513039626N1	24	51.46	3.86	12	42.17	2.76	9.29 [6.84, 11.74]	3.77
Tripp F 2014	12	38.08	15.95	15	37.6	11.01	0.48 [-9.70, 10.66]	0.67
Tung FL 2010	16	48.3	4.6	16	49.4	4.4	-1.10 [-4.22, 2.02]	3.21
Yen CL 2008	7	52.43	2.88	7	51.57	3.1	0.86 [-2.27, 3.99]	3.19
NCT00243919a	128	43.3	11.3	68	41.5	12.8	1.80 [-1.68, 5.28]	2.92
NCT00243919b	120	44.5	11.3	68	41.5	12.8	3.00 [-0.53, 6.53]	2.89
Kim Y 2015	10	44.1	1.2	10	42.2	1.6	1.90 [0.66, 3.14]	4.80
Shin W 2011	11	45.6	7.5	10	43.4	8.5	2.20 [-4.64, 9.04]	1.28
Madhuranga P 2019	14	40.8	4.81	15	37.26	3.76	3.54 [0.41, 6.67]	3.20
**Overall**							2.90 [2.01, 3.79]	

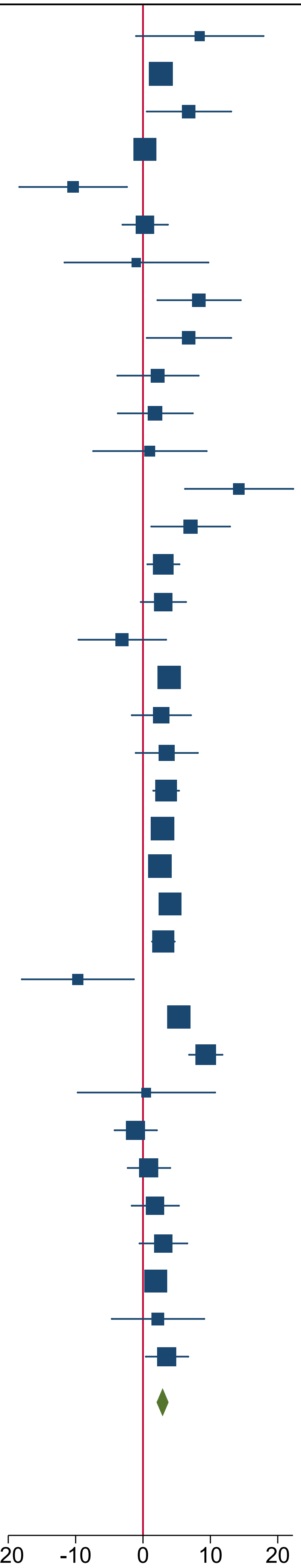

Heterogeneity: $\tau^2 = 3.88$, $I^2 = 86.27\%$, $H^2 = 7.28$

Test of $\theta_i = \theta_j$: $Q(35) = 133.96$, $p = 0.00$

Test of $\theta = 0$: $z = 6.40$, $p = 0.00$

Random-effects REML model

figure S10: Forest plot on trials reporting berg balance scale

Study	Exercise N	Exercise Mean	Exercise SD	Control N	Control Mean	Control SD	Mean diff. with 95% CI	Weight (%)
Chung S 2019	18	22.47	16.3	17	26.88	18.75	-4.41 [-16.03, 7.21]	2.24
Kim J 2017	14	33.26	26.91	15	51.6	51.87	-18.34 [-48.75, 12.07]	0.41
Kim N 2016	10	25.9	10.51	7	21.81	8.94	4.09 [-5.48, 13.66]	2.98
ISRCTN41026907	20	8.4	6	20	9	5	-0.60 [-4.02, 2.82]	7.61
Park G 2016	20	21.5	3.4	20	23.5	2.9	-2.00 [-3.96, -0.04]	8.99
Choi J 2015	12	14.42	2.85	12	15.12	7.42	-0.70 [-5.20, 3.80]	6.53
Lee YH 2015	14	.75	.1	12	.68	.11	0.07 [-0.01, 0.15]	9.86
Son S 2014	14	18.6	.5	14	25.1	1.9	-6.50 [-7.53, -5.47]	9.61
Chen C 2014	21	11.5	1	15	13.5	.7	-2.00 [-2.59, -1.41]	9.78
Iqbal M 2020	32	10.81	2.37	32	19.18	2.96	-8.37 [-9.68, -7.06]	9.45
Qurat ul Ain 2018	15	26.22	12.3	15	16.57	7.1	9.65 [2.46, 16.84]	4.28
IRCT20180513039626N1	24	8.38	1.6	12	10.66	1.59	-2.28 [-3.39, -1.17]	9.57
Kim J 2018	7	23.59	19.41	6	24.52	11.08	-0.93 [-18.56, 16.70]	1.12
Kim H 2015	11	19.91	3.53	11	21.18	4.21	-1.27 [-4.52, 1.98]	7.79
Dong-Hyun K 2019	18	6.45	1.09	18	6.88	.74	-0.43 [-1.04, 0.18]	9.77
**Overall**							-1.88 [-3.86, 0.09]	

Heterogeneity: $\tau^2 = 10.32$, $I^2 = 97.89\%$, $H^2 = 47.35$

Test of $\theta_i = \theta_j$: Q(14) = 386.78, p = 0.00

Test of $\theta = 0$: z = -1.87, p = 0.06

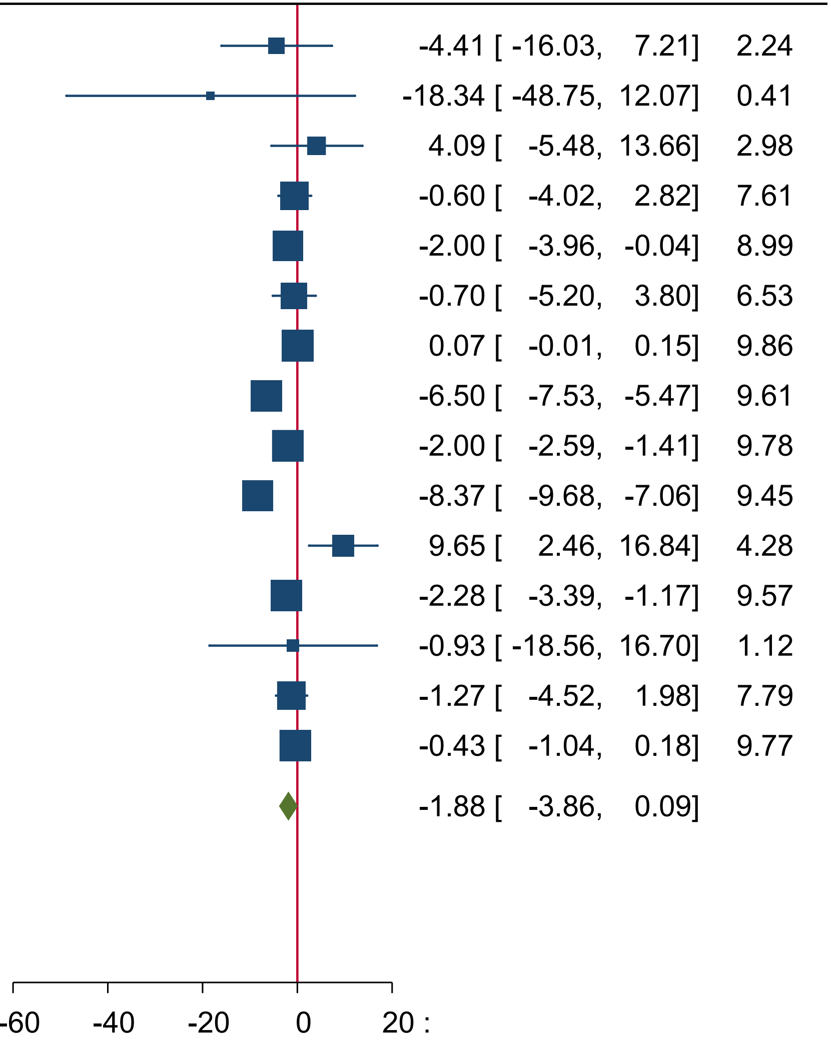

Random-effects REML model

figure S11: Forest plot on trials reporting TUGT

Study	Exercise N	Exercise Mean	Exercise SD	Control N	Control Mean	Control SD	Mean diff. with 95% CI	Weight (%)
Cider A 2003	15	84	23	10	74	25	10.00 [-9.05, 29.05]	7.35
Giannuzzi P 2003/ELVD-CHF	45	93	23	44	78	18	15.00 [6.41, 23.59]	13.30
NCT00176319	30	84	28.82	30	63.5	2.98	20.50 [10.13, 30.87]	12.15
Klecha A 2007	25	143.8	31.7	25	101.8	21.9	42.00 [26.90, 57.10]	9.28
Nilsson BB 2008	40	85	26	40	72	20	13.00 [2.83, 23.17]	12.28
Passino C 2008	71	114	5	19	92	7	22.00 [19.23, 24.77]	16.33
Passino C 2006	44	114	6	41	80	5	34.00 [31.64, 36.36]	16.45
Tynni-Lenne R 1997	8	58	10	8	28	8.9	30.00 [20.72, 39.28]	12.86
**Overall**							23.76 [16.87, 30.64]	

Heterogeneity: $\tau^2 = 73.56$, $I^2 = 90.03\%$, $H^2 = 10.03$

Test of $\theta_i = \theta_j$: Q(7) = 68.97, p = 0.00

Test of $\theta = 0$: z = 6.76, p = 0.00

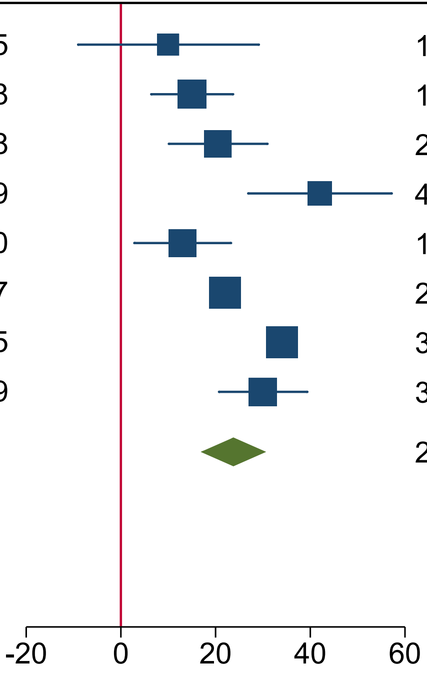

Random-effects REML model

figure S12: Forest plot on trials reporting exercise capacity (watt)

Study	Exercise N	Exercise Mean	Exercise SD	Control N	Control Mean	Control SD	Mean diff. with 95% CI	Weight (%)
Luk TH 2012	32	2.19	1.43	32	.27	1.12	1.92 [1.29, 2.55]	22.46
Vasiliauskas D 2007	83	8.2	2.7	71	6.6	1.8	1.60 [0.86, 2.34]	20.37
Tsai J 2004	52	9.8	2	50	9.2	1.8	0.60 [-0.14, 1.34]	20.35
IRCT2015092916532N3a	25	10.07	2.19	12	9.03	2.1	1.04 [-0.45, 2.53]	9.96
IRCT2015092916532N3b	25	10.81	1.76	13	9.03	2.1	1.78 [0.52, 3.04]	12.32
Kurzaj M 2019	32	8.46	2.34	30	8.17	2.01	0.29 [-0.80, 1.38]	14.53
**Overall**							1.24 [0.67, 1.81]	

Heterogeneity: $\tau^2 = 0.27$, $I^2 = 57.16\%$, $H^2 = 2.33$

Test of $\theta_i = \theta_j$: $Q(5) = 11.77$, $p = 0.04$

Test of $\theta = 0$: $z = 4.28$, $p = 0.00$

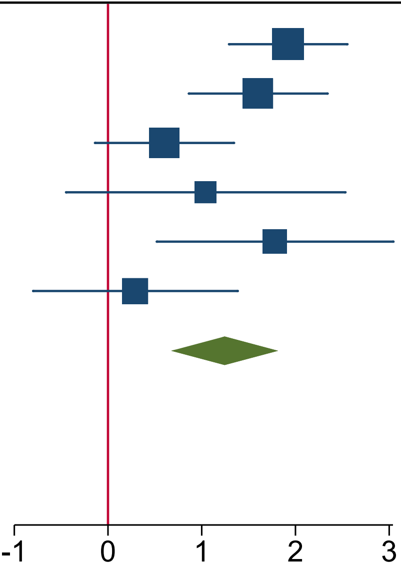

Random-effects REML model

figure S13: Forest plot on trials reporting exercise capacity (MET)

figure S14: Funnel plot on trials reporting body weight

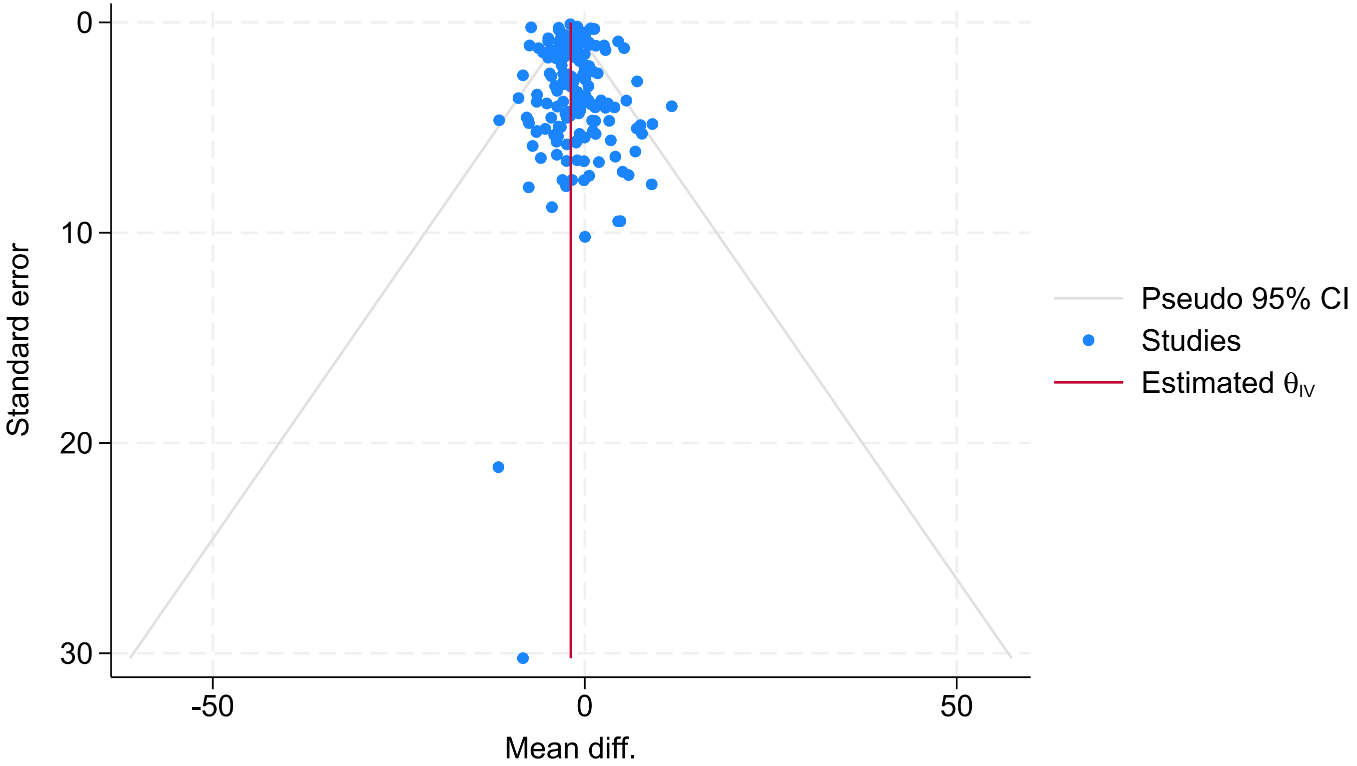

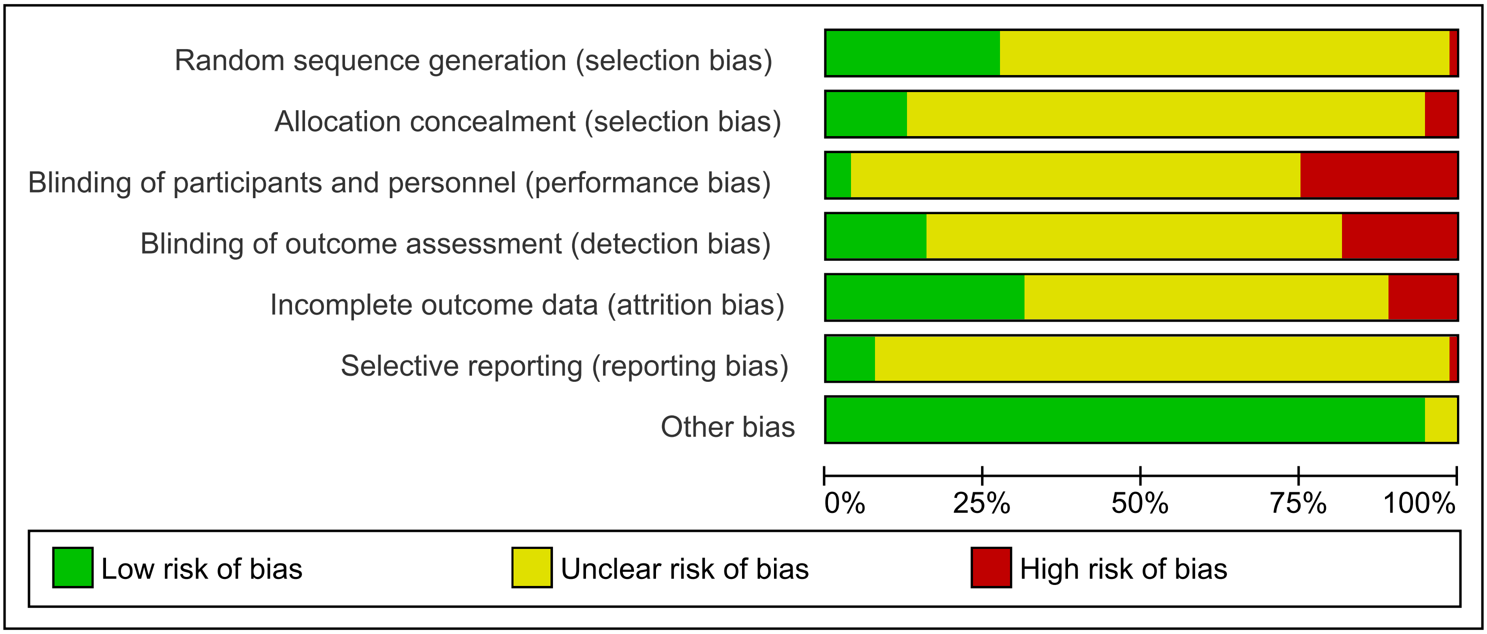

figure S15: Risk of bias graph for trials reporting body weight

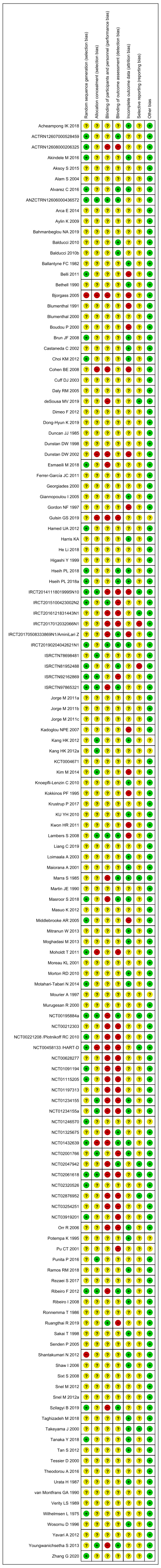

figure S16: Risk of bias summary for body weight
